# Desmopressin to reduce periprocedural bleeding and transfusion: a systematic review and meta-analysis

**DOI:** 10.1186/s13741-023-00358-4

**Published:** 2024-01-23

**Authors:** Carol Wang, Victoria Lebedeva, Jeffy Yang, Joshua Anih, Lily J. Park, Freeman Paczkowski, Pavel S. Roshanov

**Affiliations:** 1 Department of Medicine, Western University, London, ON Canada; 2 London Health Sciences Centre, London, ON Canada; 3 Schulich School of Medicine & Dentistry, Western University, London, ON Canada; 4 McMaster University, Hamilton, ON Canada; 5 Department of Surgery, Division of General Surgery, McMaster University, Hamilton, ON Canada; 6 Department of Epidemiology and Biostatistics, Western University, London, ON Canada; 7 Population Health Research Institute, Hamilton, ON Canada

**Keywords:** Desmopressin, Bleeding, Transfusion, Systematic review

## Abstract

We systematically reviewed the literature to investigate the effects of peri-procedural desmopressin in patients without known inherited bleeding disorders undergoing surgery or other invasive procedures. We included 63 randomized trials (4163 participants) published up to February 1, 2023. Seven trials were published after a 2017 Cochrane systematic review on this topic. There were 38 trials in cardiac surgery, 22 in noncardiac surgery, and 3 in non-surgical procedures. Meta-analyses demonstrated that desmopressin likely does not reduce the risk of receiving a red blood cell transfusion (25 trials, risk ratio [RR] 0.95, 95% confidence interval [CI] 0.86 to 1.05) and may not reduce the risk of reoperation due to bleeding (22 trials, RR 0.75, 95% CI 0.47 to 1.19) when compared to placebo or usual care. However, we demonstrated significant reductions in number of units of red blood cells transfused (25 trials, mean difference -0.55 units, 95% CI − 0.94 to − 0.15), total volume of blood loss (33 trials, standardized mean difference − 0.40 standard deviations; 95% CI − 0.56 to − 0.23), and the risk of bleeding events (2 trials, RR 0.45, 95% CI 0.24 to 0.84). The certainty of evidence of these findings was generally low. Desmopressin increased the risk of clinically significant hypotension that required intervention (19 trials, RR 2.15, 95% CI 1.36 to 3.41). Limited evidence suggests that tranexamic acid is more effective than desmopressin in reducing transfusion risk (3 trials, RR 2.38 favoring tranexamic acid, 95% CI 1.06 to 5.39) and total volume of blood loss (3 trials, mean difference 391.7 mL favoring tranexamic acid, 95% CI − 93.3 to 876.7 mL). No trials directly informed the safety and hemostatic efficacy of desmopressin in advanced kidney disease. In conclusion, desmopressin likely reduces periprocedural blood loss and the number of units of blood transfused in small trials with methodologic limitations. However, the risk of hypotension needs to be mitigated. Large trials should evaluate desmopressin alongside tranexamic acid and enroll patients with advanced kidney disease.

**Supplementary Information:**

The online version contains supplementary material available at 10.1186/s13741-023-00358-4.

## Background

Perioperative bleeding significantly increases the risks of perioperative morbidity and mortality;(Kamel et al. 2012; Devereaux et al. 2014; Poise Study Group, et al. 2008; Roshanov et al. 2021; Smilowitz et al. 2016) its association with mortality persists for weeks to months after postoperative discharge (Roshanov et al. 2024). Various therapeutic strategies, including antifibrinolytic agents, concentrated coagulation factors, and desmopressin, have been shown to reduce perioperative bleeding in surgical and trauma settings (Desai et al. 2018; Ghadimi et al. 2016). Tranexamic acid (TXA) has been studied extensively (Ker et al. 2012; CRASH-2 trial collaborators, et al. 2010; Devereaux et al. 2022; HALT-IT Trial Collaborators 2020) and is gaining widespread adoption. However, its effectiveness in reducing major perioperative bleeding is relatively modest, with reported absolute risk reductions between 2 and 3% (Devereaux et al. 2022). Consequently, the need to safely reduce perioperative bleeding and transfusion persists.

Desmopressin is a synthetic analog of vasopressin that acts on type 2 vasopressin receptors to promote hemostasis (Shah et al. 2020; Desborough et al. 2017; Ozgonenel et al. 2007). Although its exact hemostatic mechanism of action is not fully elucidated,(Coppola and Minno 2008) desmopressin is known to stimulate Weibel-Palade bodies of endothelial cells to release von Willebrand factor (vWF) and increase factor VIII levels, enhanced platelet aggregation and adherence, and reduce bleeding time (Leissinger et al. 2014; Mannucci et al. 1981; Rossaint et al. 2016).

This systematic review provides an update of a 2017 Cochrane Review regarding the hemostatic efficacy of periprocedural desmopressin (Desborough et al. 2017). Our rationale for updating this review includes (A) most of the outcomes pertaining to the therapeutic effects of desmopressin in this high-quality 2017 review had low or very low certainty and updating with recent studies may strengthen the certainty of conclusions drawn from the review. (B) of the 65 studies included in the previous review, 46 were published more than 20 years ago. (Desborough et al. 2017) With the adoption of restrictive transfusion strategies recommended by the American Association of Blood Banks (AABB) clinical practice guideline in 2012,(Carson et al. 2012) updating the review with newer studies may better represent recent clinical practice. (C) Previous reviews did not report the baseline kidney function of participants,(Desborough et al. 2017; Carless et al. 2004; Crescenzi et al. 2008; Fremes et al. 1994; Laupacis and Fergusson 1997; Levi et al. 1999) which is important to consider because individuals with kidney dysfunction may have a propensity for bleeding disorders due to uremic platelet dysfunction and because of concerns about hyponatremia (Acedillo et al. 2013).

## Methods

We report this review according to the Preferred Reporting Items for Systematic Reviews and Meta-Analyses (PRISMA) guidelines (Page et al. 2021). The protocol was written and registered before undertaking the review (PROSPERO #CRD42023396458).

### Study eligibility

We looked for randomized controlled trials (RCTs) that compared the effects of desmopressin administered intravenously or subcutaneously before, during, or immediately after a surgical or interventional procedure to placebo, usual care, or antifibrinolytic agents (i.e., TXA, $\in$-aminocaproic acid, or aprotinin). We included trials that enrolled children or adults without congenital bleeding disorders (i.e., von Willebrand disease). The outcomes of primary interest were (a) total perioperative volume of blood loss (measured in milliliters in adults and milliliters per kilogram in children), (b) number of participants who received red blood cell (RBC) transfusion (or, said another way, the risk of receiving any RBC transfusion), and (c) units of RBCs transfused. The transfusion thresholds and protocols were based on investigator definitions. Other outcomes included in this review were (a) hypotension, (b) nausea, (c) facial flushing, (d) seizures of any type, (e) all-cause mortality, (f) reoperation due to bleeding, (g) cardiovascular events (i.e., myocardial infarction and stroke), (h) venous thromboembolism, and (i) hyponatremia. All outcomes were recorded according to the definitions provided by individual study investigators and eligible if ascertained within 30 days of the procedure.

### Identification of studies

We used the search strategy outlined in the 2017 Cochrane review (Desborough et al. 2017) to identify relevant RCTs. Assisted by a library information specialist, we conducted searches in the following databases for trials published between January 1, 2017, and February 1, 2023: the Cochrane Central Register of Controlled Trials (CENTRAL), MEDLINE via OVID, PubMed, Embase, Cumulative Index to Nursing and Allied Health Literature (CINAHL), Transfusion Evidence Library, Web of Science Conference Proceedings Citation Index, Latin American and Caribbean Health Sciences Literature (LILACS), KoreaMed, PakMediNet, and the University of Hong Kong Clinical Trials Registry (Desborough et al. 2017). Complete search strategies can be found in the Additional file 1. All references were entered into Covidence (Veritas Health Innovation LTD), and duplicate study records were removed. Additionally, we manually searched the bibliographies of the relevant trials for eligible studies. We imposed no restrictions on language or publication status.

Two investigators independently screened the titles and abstracts of all identified studies against a pre-specified checklist (see Additional file 1). The full texts of potentially relevant studies were reviewed for eligibility. Disagreements between them were resolved through discussion and consensus, with consulting a third-party expert (PSR) when necessary. The studies identified from 2017 onwards were collated with the studies previously identified in the Cochrane review to generate the final set of included studies.

### Data extraction and management

Two independent reviewers performed data extraction in duplicate using a standard data extraction form that included: (a) study characteristics (i.e., publication year, study type), (b) participant characteristics (i.e., age, sex), (c) indication for desmopressin (i.e., surgical vs. interventional procedure), (d) intervention characteristics (i.e., route and dose of desmopressin administered), (e) details of the comparator (i.e., agent name, route of administration, dose), (f) transfusion protocol per individual study, and (g) efficacy and safety outcomes.

### Assessment of risk of bias

Two reviewers independently assessed the risk of bias in each study using the revised Cochrane Risk of Bias (RoB 2.0) tool (Higgins et al. 2023). An overall risk-of-bias judgement of ‘high’ or ‘low’ risk or ‘some concerns’ was established for each outcome in the included studies. Disagreements on risk-of-bias between the two reviewers were resolved by discussion. We updated the risk-of-bias evaluations of studies captured in the previous review by mapping the risk-of-bias assessments based on Cochrane RoB 1.0 to domains of the Cochrane RoB 2.0 for each included study. To validate this approach, a reviewer who was blinded to the results of the initial risk-of-bias evaluation applied the RoB 2.0 tool to two studies selected at random, and the mapping yielded similar results (Additional file 1: Table S1).

### Measures of treatment effect and data synthesis

We estimated heterogeneity in meta-analyses using the DerSimonian-Laird random effects model. For dichotomous outcomes, we calculated the pooled risk ratio (RR) and 95% confidence interval (CI) using the Mantel–Haenszel method. We presented continuous outcomes as means and standard deviations and quantified effects using the standardized mean difference (SMD) or mean difference (MD) and 95% CI with meta-analysis using the generic inverse variance method (Desborough et al. 2017). All quantitative data syntheses were performed in R version 4.3 (RStudio Team 2020). Where meta-analysis was not feasible, we provided a qualitative summary of the findings from eligible RCTs.

### Approach to missing data

We contacted study authors for relevant missing data where possible. We documented the number of participants randomized compared with the analyzed in each study. We analyzed patients with available data in the groups to which they were allocated (i.e., intention to treat).

### Assessment of heterogeneity

Heterogeneity assessment involved (a) visual inspection of the forest plots to assess the degree of overlap in CIs between individual studies; (b) Cochrane’s *Q* test, with a *p* value < 0.10 indicating statistical heterogeneity and rejecting the null hypothesis that the individual studies are homogeneous in their measured effects; and (c) interpretation of the *I*^2^ statistic, where moderate heterogeneity is defined as *I*^2^ of 50 to 80% and considerable heterogeneity as *I*^2^ > 80% (Desborough et al. 2017). If statistical heterogeneity was detected, we performed tests for subgroup differences to elucidate potential sources of heterogeneity, with a *p* value of < 0.10 in the test of subgroup differences indicating effect modification contributing to heterogeneity.

### Assessment of reporting biases

Publication bias was assessed by visual assessment of funnel plots.

### Subgroup analysis and investigation of heterogeneity

We prespecified several subgroups for evaluation with statistical tests of interaction: (a) comparator group receiving placebo or usual care versus active comparator such as TXA, (b) higher versus lower baseline kidney function, (c) subcutaneous versus intravenous administration, (d) type of procedural intervention (i.e., cardiac surgery versus non-cardiac surgery versus non-surgical procedures), (e) use versus non-use of antiplatelet agents at baseline; (f) use versus non-use of anticoagulants at baseline, and (g) adult versus pediatric participants.

### Sensitivity analyses

A prespecified sensitivity analysis limited inclusion to studies judged to be at low risk of bias. Post-hoc analyses of outcomes pertaining to blood transfusion limited inclusion to studies published since 2012.

### Assessment of certainty of the evidence

We used the Grading of Recommendations, Assessment, Development and Evaluation (GRADE) approach to assess the certainty of pooled effect estimates (Schünemann 2022).

### Trial sequential analysis

We applied trial sequential analysis (TSA) to assess whether, on statistical grounds, enough evidence had accumulated to obviate the need for additional trials. The information size was calculated based on a 15% reduction in relative risk for dichotomous outcomes and a 15% reduction in mean differences for continuous outcomes, in alignment with the previous review. We applied O’Brien Fleming sequential monitoring boundaries for efficacy and futility with 80% statistical power and *α* = 0.05 (Wetterslev et al. 2017; Thorlund et al. 2017).

## Results

### Included studies

**Fig. 1:**
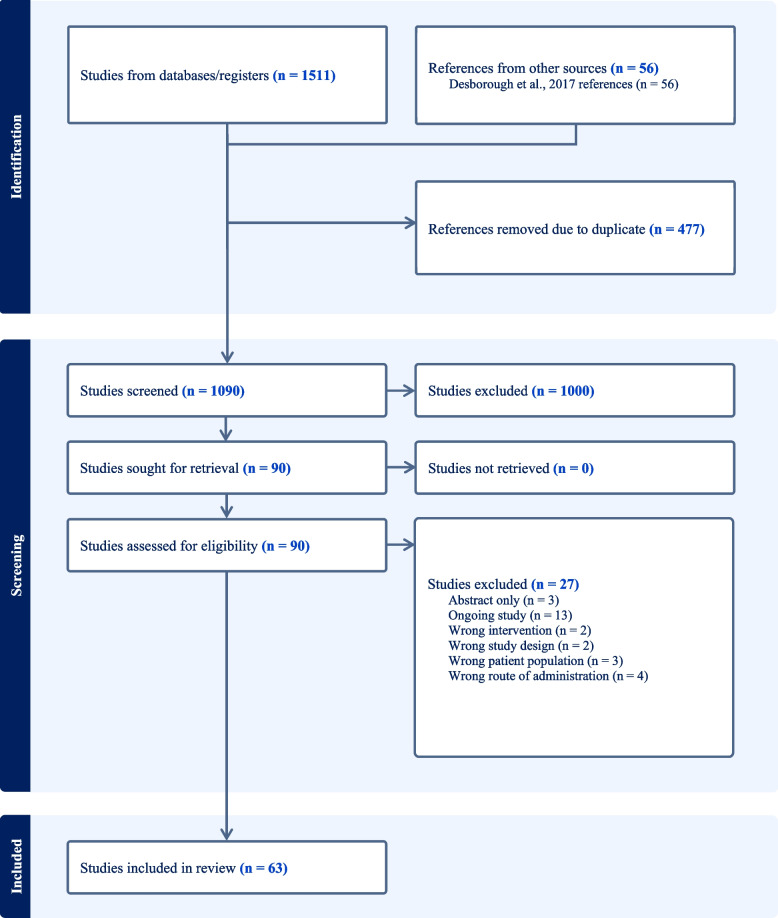
Study flow diagram (generated from Covidence)

Figure 1 summarizes the flow of records and Additional file 1: Table S2 summarizes the characteristics of included studies. A total of 63 trials, involving 4163 participants were included (Lee et al. 2010; Marczinski and Meer 2007; Zohar et al. 2001; Wong et al. 2003; Wingate et al. 1992; Temeck et al. 1994; Steinlechner et al. 2011; Spyt et al. 1990; Sheridan et al. 1994; Shao et al. 2015; Seear et al. 1989; Schött et al. 1995; Salzman et al. 1986; Salmenperä et al. 1991; Rocha et al. 1988; Reynolds et al. 1993; Reich et al. 1991; Pleym et al. 2004; Ozkisacik et al. 2001; Oliver et al. 2000; Mongan and Hosking 1992; Marquez et al. 1992; Manno et al. 2011; Letts et al. 1998; Lethagen et al. 1991; Leino et al. 2010; Kuitunen 1992; Kobrinsky et al. 1987; Karnezis et al. 1994; Jin and Ji 2015; Horrow et al. 1991; Hedderich et al. 1990; Hajjar et al. 1853; Hackmann et al. 1989; Guyuron et al. 1996; Guay et al. 1992; Gratz et al. 1992; Frankville et al. 1991; Flordal et al. 1992; Flordal et al. 1991; Ellis et al. 2001; Dilthey et al. 1993; Despotis et al. 1999; Prost et al. 1992; Clagett et al. 1995; Chuang et al. 1993; Casas et al. 1995; Brown et al. 1989; Bignami et al. 2016; Ansell et al. 1992; Andersson et al. 1990; Altun et al. 2017; Wang et al. 2020; Hajimohamadi et al. 2021; Jahangirifard et al. 2017; Desborough et al. 2022; Youssefy et al. 2022; (Vafaee M, Alizadeh A, Gholamian A: Evaluation of preoperative intravenous desmopressin on blood loss in major spine surgery, unpublished). Thirty-eight studies examined desmopressin in cardiac surgery,(Temeck et al. 1994; Steinlechner et al. 2011; Spyt et al. 1990; Sheridan et al. 1994; Seear et al. 1989; Salzman et al. 1986; Salmenperä et al. 1991; Rocha et al. 1988; Reynolds et al. 1993; Reich et al. 1991; Pleym et al. 2004; Ozkisacik et al. 2001; Oliver et al. 2000; Mongan and Hosking 1992; Marquez et al. 1992; Lethagen et al. 1991; Kuitunen 1992; Jin and Ji 2015; Horrow et al. 1991; Hedderich et al. 1990; Hajjar et al. 1853; Hackmann et al. 1989; Gratz et al. 1992; Frankville et al. 1991; Dilthey et al. 1993; Despotis et al. 1999; Prost et al. 1992; Chuang et al. 1993; Casas et al. 1995; Brown et al. 1989; Bignami et al. 2016; Ansell et al. 1992; Andersson et al. 1990; Altun et al. 2017; Jahangirifard et al. 2017) twenty-two in non-cardiac surgery,(Marczinski and Meer 2007; Zohar et al. 2001; Wong et al. 2003; Wingate et al. 1992; Shao et al. 2015; Schött et al. 1995; Letts et al. 1998; Leino et al. 2010; Kobrinsky et al. 1987; Karnezis et al. 1994; Guyuron et al. 1996; Guay et al. 1992; Flordal et al. 1992; Flordal et al. 1991; Ellis et al. 2001; Clagett et al. 1995; Wang et al. 2020; Hajimohamadi et al. 2021; Youssefy et al. 2022; (Vafaee M, Alizadeh A, Gholamian A: Evaluation of preoperative intravenous desmopressin on blood loss in major spine surgery, unpublished)), and three in non-surgical procedures.(Lee et al. 2010; Manno et al. 2011; Desborough et al. 2022) Seven (Altun et al. 2017; Wang et al. 2020; Hajimohamadi et al. 2021; Jahangirifard et al. 2017; Desborough et al. 2022; Youssefy et al. 2022; (Vafaee M, Alizadeh A, Gholamian A: Evaluation of preoperative intravenous desmopressin on blood loss in major spine surgery, unpublished)) of these were not included in the previous review: five full reports published between 2017 and 2022 (Altun et al. 2017; Wang et al. 2020; Hajimohamadi et al. 2021; Jahangirifard et al. 2017; Youssefy et al. 2022), one abstract supplemented by clinical trial registry information,(Desborough et al. 2022; Laing 2023) and one unpublished trial manuscript provided by the author (Vafaee M, Alizadeh A, Gholamian A: Evaluation of preoperative intravenous desmopressin on blood loss in major spine surgery, unpublished).

### Risk of bias

The risk-of-bias assessments for the seven recent studies (Altun et al. 2017; Wang et al. 2020; Hajimohamadi et al. 2021; Jahangirifard et al. 2017; Desborough et al. 2022; Youssefy et al. 2022; (Vafaee M, Alizadeh A, Gholamian A: Evaluation of preoperative intravenous desmopressin on blood loss in major spine surgery, unpublished)) are presented by outcome in Additional file 1: Figure S1. Our mapping of risk-of-bias assessments of studies in the previous review is presented in Additional file 1: Figure S2. Amongst the studies captured in the previous review, there was only one study judged to be at low risk of bias (Bignami et al. 2016). The majority of the included studies were at high risk of bias, especially with concerns arising from domains of deviation from the randomization process, intended intervention, and concerns for selection of reported result.

### Meta-analyses

Table 1 summarizes the main findings and GRADE assessment for each outcome and the results of subgroup analyses. The previous review divided some studies into separate comparisons (Desborough et al. 2017). For the most part, these comparisons were kept the same, with minor exceptions detailed in the Additional file 1.

**Table 1:** Summary of findings for comparison of desmopressin to various comparator groups examining the hemostatic outcomes and potential adverse effects of desmopressin

**Outcomes**	**No. of participants (studies)**	Relative effect (95% CI)*	**Absolute effect (95% CI)**	***p* value for the main effect**	***p* value for subgroup effect**	**Accrued IS/required IS; monitoring boundaries; interpretation**‡	**Quality of the evidence (GRADE)**
**Desmopressin vs placebo or usual care**							
** Need for red blood cell transfusion**¶	1944 (28)	RR 0.95 [0.86, 1.05]	**21 fewer per 1000** (58 fewer to 21 more)	0.306		1944/2360; futility boundary crossed; 15% RRR unlikely; inconclusive regarding smaller effects	⨁⨁⨁◯ Moderate^a,^^∏^
** Cardiac surgery**	1460 (19)	RR 0.94 [0.83, 1.06]			0.887		
** Non-cardiac surgery**	282 (7)	RR 1.01 [0.83, 1.24]					
** Interventional procedure**	202 (2)	RR 0.85 [0.23, 3.07]					
** Baseline antiplatelet use**	759 (9)	RR 0.95 [0.82, 1.09]			0.632		
** No baseline antiplatelet use**	626 (9)	RR 0.89 [0.73, 1.08]					
** ≥ 2012**	325 (4)	RR 0.84 [0.49, 1.43]			0.923		
** < 2012**	1619 (24)	RR 0.95 [0.85, 1.05]					
** Total volume of blood loss**¶	2037 (35)	–-	**0.40 SD lower** (0.56 lower to 0.23 lower)	< 0.001		2037/593; monitoring boundaries crossed; conclusive for 15% reduction	⨁⨁◯◯ Low^a,b,^^∏^
** Cardiac surgery**	1581 (26)	SMD − 0.39 [− 0.59, − 0.18]			0.768		
** Non-cardiac surgery**	456 (9)	SMD − 0.42 [− 0.64, − 0.21]					
** Baseline antiplatelet use**	500 (8)	SMD − 0.31 [− 0.61, − 0.02]			0.676		
** No baseline antiplatelet use**	572 (12)	SMD − 0.38 [− 0.59, − 0.17]					
** ≥ 2012**	317 (6)	SMD − 0.76 [− 1.19, − 0.32]			0.051		
** < 2012**	1720 (29)	SMD − 0.32 [− 0.49, − 0.16]					
** Adult**	1817 (31)	SMD − 0.43 [− 0.60, − 0.26]			0.262		
** Pediatric**	220 (4)	SMD − 0.12 [− 0.46, 0.22]					
** Units of red blood cells transfused**¶	1601 (26)	–-	**0.55 fewer units** (0.15 fewer to 0.94 fewer)	0.007		1601/1683; monitoring boundaries not crossed; inconclusive for 15% reduction	⨁⨁◯◯Low^a,b,^^∏^
** Cardiac surgery**	1025 (16)	MD − 0.71 [− 1.22, − 0.20]			0.306		
** Non-cardiac surgery**	576 (10)	MD − 0.29 [− 0.92, 0.35]					
** Baseline antiplatelet use**	305 (6)	MD − 0.45 [− 1.07, 0.16]			0.837		
** No baseline antiplatelet use**	488 (8)	MD − 0.39 [− 0.68, − 0.10]					
** ≥ 2012**	123 (3)	MD − 1.76 [− 2.74, − 0.77]			0.003		
** < 2012**	1478 (23)	MD − 0.34 [− 0.68, 0.00]					
** Adult**	1566 (25)	MD − 0.53 [− 0.94, − 0.12]			0.720		
** Pediatric**	35 (1)	MD − 0.90 [− 1.70, − 0.10]					
** Any bleeding**¶	202 (2)	RR 0.45 [0.24, 0.84]	**139 fewer per 1000** (192 fewer to 40 fewer)	0.012		202/3928; monitoring boundaries not crossed; inconclusive for 15% RRR	⨁⨁◯◯ Low^a,b,^^∏^
** Reoperation due to bleeding**	1831 (24)	RR 0.75 [0.47, 1.19]	**11 fewer per 1000** (23 fewer to 8 more)	0.223		1831/28364; monitoring boundaries not crossed; inconclusive for 15% RRR	⨁⨁◯◯ Low^a,b,^^∏^
** Cardiac surgery**	1591 (21)	RR 0.74 [0.46, 1.19]			0.966		
** Non-cardiac surgery**	30 (1)	RR 1.00 [0.02, 47.38]					
** Interventional procedure**	210 (2)	RR 1.01 [0.06, 15.89]					
** Baseline antiplatelet use**	722 (8)	RR 1.00 [0.47, 2.11]			0.816		
** No baseline antiplatelet use**	504 (8)	RR 0.79 [0.23, 2.73]					
** ≥ 2012**	183 (2)	RR 0.67 [0.30, 1.50]			0.460		
** < 2012**	1648 (22)	RR 0.80 [0.45, 1.43]					
** Adult**	1741 (22)	RR 0.73 [0.45, 1.16]			0.753		
** Pediatric**	90 (2)	RR 1.86 [0.16, 21.41]					
** Myocardial infarction**	1866 (28)	RR 1.22 [0.75, 1.98]	**5 more per 1000** (6 fewer to 22 more)	0.429		1866/57475; monitoring boundaries not crossed; inconclusive for 15% RRR	⨁⨁◯◯ Low^a,b,^^∏^
** Cardiac surgery**	1129 (17)	RR 1.40 [0.90, 2.47]			0.604		
** Non-cardiac surgery**	535 (9)	RR 0.76 [0.27, 2.19]					
** Interventional procedure**	202 (2)	RR 1.08 [0.75, 1.98]					
** Baseline antiplatelet use**	373 (6)	RR 1.13 [0.39, 3.27]			0.970		
** No baseline antiplatelet use**	796 (11)	RR 1.15 [0.60, 2.21]					
** ≥ 2012**	280 (4)	RR 1.03 [0.18, 5.82]			0.847		
** < 2012**	1586 (24)	RR 1.23 [0.74, 2.05]					
** Stroke**	1399 (20)	RR 1.31 [0.61, 2.84]	**1 more per 1000** (2 fewer to 8 more)	0.487		1399/644159; monitoring boundaries not crossed; inconclusive for 15% RRR	⨁⨁◯◯ Low^a,b,^^∏^
** Cardiac surgery**	831 (12)	RR 1.51 [0.60, 3.85]			0.868		
** Non-cardiac surgery**	406 (7)	RR 0.96 [0.22, 4.14]					
** Interventional procedure**	162 (1)	RR 1.02 [0.02, 51.03]					
** Baseline antiplatelet use**	223 (4)	RR 1.43 [0.28, 7.38]			0.785		
** No baseline antiplatelet use**	479 (5)	RR 1.03 [0.20, 5.41]					
** ≥ 2012**	240 (3)	RR 0.99 [0.10, 9.35]			0.791		
** < 2012**	1159 (17)	RR 1.37 [0.60, 3.10]					
** Hypotension**	1321 (20)	RR 2.15 [1.36, 3.41]	**29 more per 1000** (9 more to 60 more)	0.001		1321/49272; monitoring boundaries not crossed; inconclusive for 15% RRR	⨁⨁⨁◯ Moderate^a,^^∏^
** Cardiac surgery**	872 (14)	RR 2.39 [1.26, 4.53]			0.896		
** Non-cardiac surgery**	247 (4)	RR 1.91 [0.96, 3.80]					
** Interventional procedure**	202 (2)	RR 2.08 [0.18, 24.12]					
** Baseline antiplatelet use**	511 (8)	RR 2.95 [1.20, 7.28]			0.506		
** No baseline antiplatelet use**	581 (8)	RR 2.06 [1.18, 3.60]					
** ≥ 2012**	313 (4)	RR 1.56 [0.41, 5.95]			0.616		
** < 2012**	1008 (16)	RR 2.25 [1.37, 3.67]					
** Adult**	1231 (18)	RR 2.23 [1.40, 3.58]			0.443		
** Pediatric**	90 (2)	RR 0.92 [0.10, 8.50]					
** Venous thromboembolism**	1467 (21)	RR 0.81 [0.38, 1.76]	**2 fewer per 1000** (5 fewer to 6 more)	0.597		1467/166587; monitoring boundaries not crossed; inconclusive for 15% RRR	⨁⨁◯◯ Low^a,b,^^∏^
** Cardiac surgery**	804 (11)	RR 0.71 [0.25, 2.02]			0.869		
** Non-cardiac surgery**	461 (8)	RR 1.31 [0.32, 5.40]					
** Interventional procedure**	202 (2)	RR 1.02 [0.02, 51.03]					
** Baseline antiplatelet use**	311 (5)	RR 0.85 [0.18, 4.05]			0.949		
** No baseline antiplatelet use**	577 (7)	RR 0.91 [0.223, 3.61]					
** ≥ 2012**	232 (3)	RR 0.49 [0.07, 3.72]			0.599		
** < 2012**	1235 (18)	RR 0.88 [0.38, 2.04]					
** Hyponatremia (dichotomous)**	254 (3)	RR 2.02 [0.53, 7.72]	**16 more per 1000** (7 fewer to 105 more)	0.307		254/84520; monitoring boundaries not crossed; inconclusive for 15% RRR	⨁◯◯◯ Very low^a,b,^^∏^
** Post-procedural serum sodium**	103 (2)	–-	**0.01 mmol/L lower** (1.97 lower to 1.95 higher)	0.989		56/2103997; monitoring boundaries not crossed; inconclusive for 15% reduction	⨁◯◯◯ Very low^a,b,^^∏^
**Desmopressin vs tranexamic acid**							
** Need for red blood cell transfusion**¶	135 (3)	RR 2.38 [1.06, 5.39]	**330 more per 1000 with desmopressin** (14 more to 1000 more)	0.037		135/9949; monitoring boundaries not crossed; inconclusive for 15% RRR	⨁⨁◯◯ Low^a,b,^^∏^
** Total volume of blood loss**¶	143 (3)	–-	**391.7 mL more with desmopressin** (93.3 less to 876.7 more)	0.113		143/749; monitoring boundary crossed; conclusive for 15% harm increase	⨁◯◯◯ Very low^a,b,^^∏^
** Volume of red blood cell transfused**¶	68 (2)	–-	**1.25 units more with desmopressin** (0.03 less to 2.52 more)	0.055		68/155; monitoring boundaries crossed; conclusive for 15% harm increase	⨁◯◯◯ Very low^a,b,^^∏^
**Desmopressin vs Aprotinin**							
** Reoperation due to bleeding**	179 (2)	RR 1.36 [0.18, 10.29]	**5 more per 1000 with desmopressin** (11 fewer to 122 more)	0.765		179/94589; monitoring boundaries not crossed; inconclusive for 15% RRR	⨁◯◯◯ Very low^a,b,^^∏^
** Myocardial infarction**	179 (2)	RR 0.72 [0.05, 11.29]§	**2 fewer per 1000 with desmopressin** (6 fewer to 68 more)§	0.814		No events observed in control arm; unable to calculate	⨁◯◯◯ Very low^a,b,^^∏^
** Stroke**	179 (2)	RR 1.47 [0.13, 17.26]§	**3 more per 1000 with desmopressin** (6 fewer to 107 more)§	0.758		No events observed in control arm; unable to calculate	⨁◯◯◯ Very low^a,b,^^∏^
** Venous thromboembolism**	179 (2)	RR 0.39 [0.03, 4.62]	**8 fewer per 1000 with desmopressin** (13 fewer to 40 more)	0.458		179/61679; monitoring boundaries not crossed; inconclusive for 15% RRR	⨁◯◯◯ Very low^a,b,^^∏^

Abbreviations: *CI* confidence interval, *IS* information size, *RR* risk ratio, *RRR* relative risk reduction, *SMD* standardized mean difference, *GRADE* Grading of Recommendations, Assessment, Development, and Evaluations. The statistical analysis presented in this report was conducted with a predefined statistical power of 80% to detect the specified effects and associations

^*^In cases where subgroups are presented, this value represents the effect within the specified subgroup

^†^In cases where subgroups are presented, this value represents the interaction *p* value

^‡^The information size was calculated based on an assumed relative risk reduction of 15% when desmopressin is administered during the periprocedural period for dichotomous outcomes. For continuous outcomes, these calculations were guided by empirical evidence on mean differences

^§^The event rate in the aprotinin group was 0 across included studies for this outcome. To estimate the relative and absolute effect estimates, the event rate was assumed to be 0.5 in the aprotinin group

^¶^The timing of ascertainment of transfusion requirements and blood loss varied from during the procedure to 96 h after (Desborough et al. 2017). Total blood loss was estimated by different techniques, including suction volume and drainage tube outputs. The volume of RBC transfusion was reported in either units or milliliters (subsequently converted to units assuming 300 mL per unit)

^a^Downgrade one level for the risk of bias attributed to limitations in reporting of the randomization process and selection of the reported result

^b^Downgrade one level for an inconsistency arising from unexplained statistical heterogeneity between studies

^c^Downgrade two level due to imprecision arising from small sample size and confidence interval including no effect

^∏^Relevant funnel plot for this outcome is found in Additional file 1: Figures S12, S13, S14, S15, S16, S17, S18, S19, S20, S21, S22, S23, S24, S25, S26, S27, S28, and S29

### Risk of receiving a red blood cell transfusion

#### Comparison to placebo or usual care

**Fig. 2:**
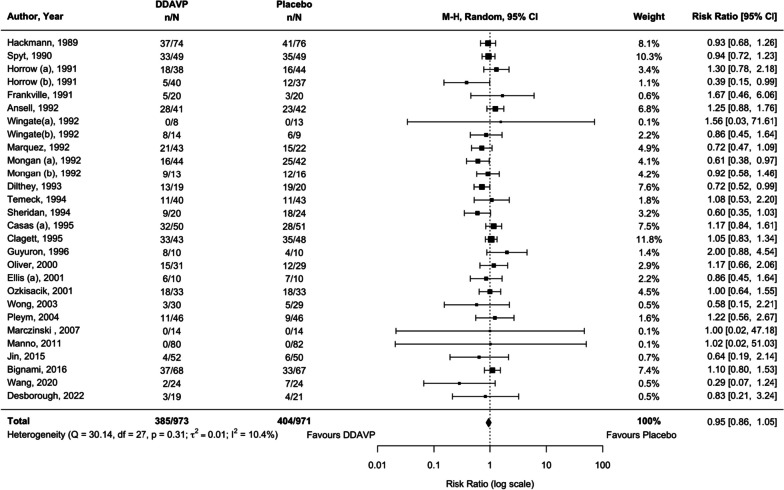
Comparison of desmopressin to placebo or usual care for the outcome of the number of participants who received a red cell transfusion among participants

Twenty-five studies (28 comparisons) (Marczinski and Meer 2007; Wong et al. 2003; Wingate et al. 1992; Temeck et al. 1994; Spyt et al. 1990; Sheridan et al. 1994; Pleym et al. 2004; Ozkisacik et al. 2001; Oliver et al. 2000; Mongan and Hosking 1992; Marquez et al. 1992; Manno et al. 2011; Jin and Ji 2015; Horrow et al. 1991; Hackmann et al. 1989; Guyuron et al. 1996; Frankville et al. 1991; Ellis et al. 2001; Dilthey et al. 1993; Clagett et al. 1995; Casas et al. 1995; Ansell et al. 1992; Wang et al. 2020; Desborough et al. 2022) examined desmopressin in relation to placebo or usual care (1944 participants). There was no evidence of an effect of desmopressin on receiving RBC transfusions (RR 0.95, 95% CI 0.86 to 1.05, moderate certainty, *I*^2^ = 10.4%, *p* = 0.306; Table 1). There was no significant statistical heterogeneity between the studies (Fig. 2). No statistical subgroup differences were detected in our pre-specified and post hoc subgroup analyses (Table 1). TSA suggested the addition of more evidence is unlikely to alter this finding (Table 1, Additional file 1: Figure S3).

#### Comparison to TXA

Three studies (Zohar et al. 2001; Horrow et al. 1991; Ellis et al. 2001) compared desmopressin to TXA (135 participants). Desmopressin was more effective at reducing exposure to RBC transfusion (RR 2.38, 95% CI 1.06 to 5.39, low certainty, *I*^2^ = 43.0%, *p* = 0.037; Table 1). TSA suggests that the evidence did not provide sufficient evidence to confidently conclude the therapeutic benefit of desmopressin (Table 1, Additional file 1: Figure S5).

### Total volume of blood loss

#### Comparison to placebo or usual care

**Fig. 3:**
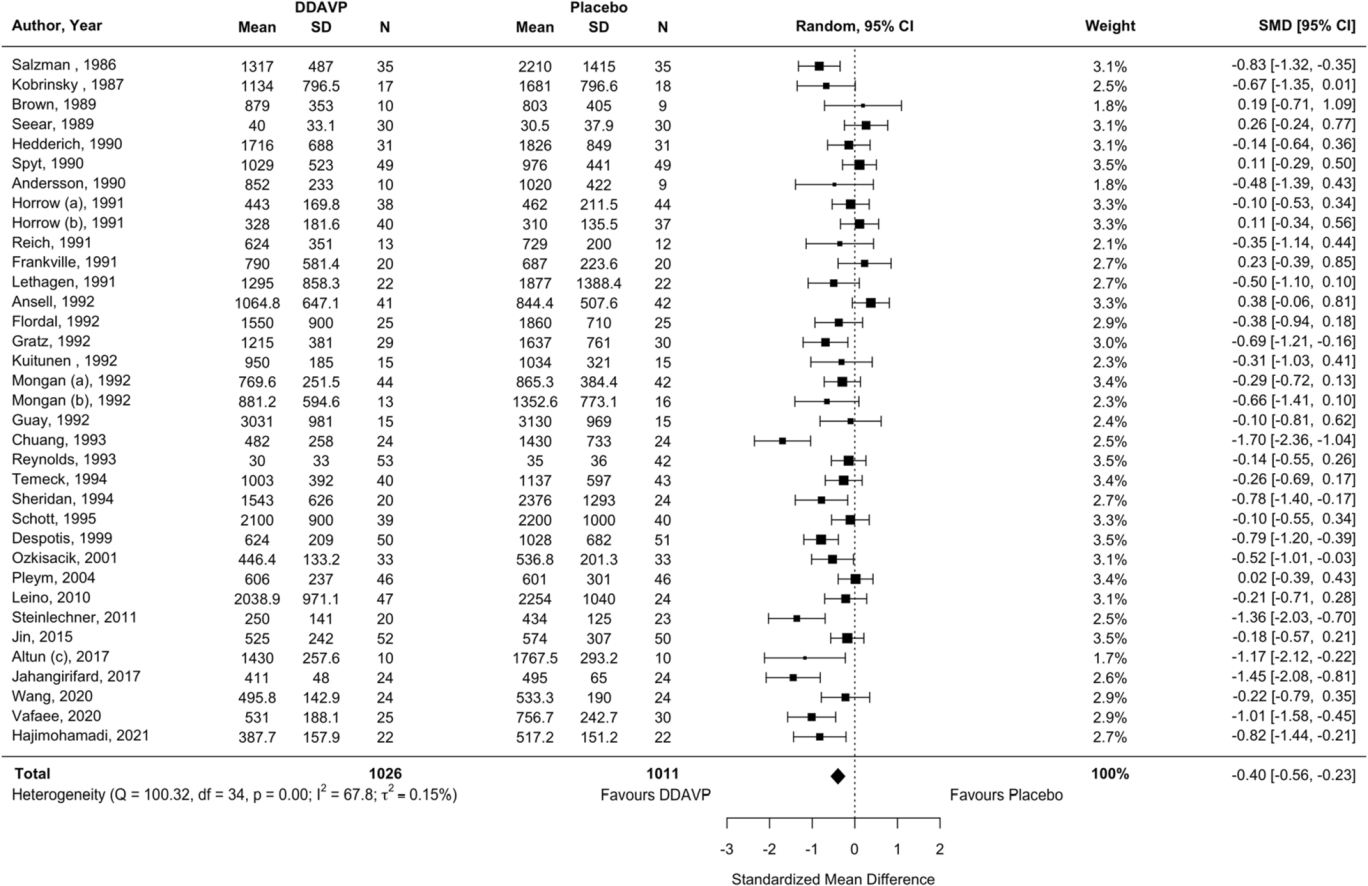
Comparison of desmopressin to placebo or usual care for the outcome of the total volume of blood loss

We analyzed 33 studies (35 comparisons) (Temeck et al. 1994; Steinlechner et al. 2011; Spyt et al. 1990; Sheridan et al. 1994; Seear et al. 1989; Schött et al. 1995; Salzman et al. 1986; Reynolds et al. 1993; Reich et al. 1991; Pleym et al. 2004; Ozkisacik et al. 2001; Mongan and Hosking 1992; Lethagen et al. 1991; Leino et al. 2010; Kuitunen 1992; Kobrinsky et al. 1987; Jin and Ji 2015; Horrow et al. 1991; Hedderich et al. 1990; Guay et al. 1992; Gratz et al. 1992; Frankville et al. 1991; Flordal et al. 1992; Despotis et al. 1999; Chuang et al. 1993; Brown et al. 1989; Ansell et al. 1992; Andersson et al. 1990; Altun et al. 2017; Wang et al. 2020; Hajimohamadi et al. 2021; Jahangirifard et al. 2017; (Vafaee M, Alizadeh A, Gholamian A: Evaluation of preoperative intravenous desmopressin on blood loss in major spine surgery, unpublished)) that reported on total blood loss (2037 participants). Although Rocha 1994 (Rocha et al. 1994) reported on the total volume of blood loss, its unit of analysis in ml/m^2^ precluded meta-analysis (Desborough et al. 2017). Additionally, the study by Altun and colleagues (Altun et al. 2017) was the full-text publication of an abstract (Hemsinli 2012) captured in the previous review. Compared with either placebo or usual care, desmopressin resulted in a small reduction in total volume of blood loss (SMD − 0.40, 95% CI − 0.56 to − 0.23, low certainty, *I*^2^ = 67.8%, *p* < 0.001; Fig. 3). TSA (94.8% of information size needed to detect or reject a 15% reduction) suggested additional evidence is unlikely to alter this conclusion (Table 1, Additional file 1: Figure S6). However, there was statistical heterogeneity between the studies. No subgroup differences were detected on evaluations stratified by type of intervention, baseline antiplatelet use, age of the participants, or publication relative to guideline update (Table 1).

#### Comparison to TXA

We have very low certainty evidence that participants who received desmopressin experienced a higher volume of total blood loss than those who received TXA (3 studies (Zohar et al. 2001; Horrow et al. 1991; Altun et al. 2017), *n* = 143 participants, MD 391.74, 95% CI − 93.25 to 876.74, *I*^2^ = 98.4%, *p* = 0.113; Table 1). TSA suggests it is unlikely that adding more evidence would alter this finding (Table 1, Additional file 1: Figure S7).

### Volume of red blood cells transfused

#### Comparison to placebo or usual care

**Fig. 4:**
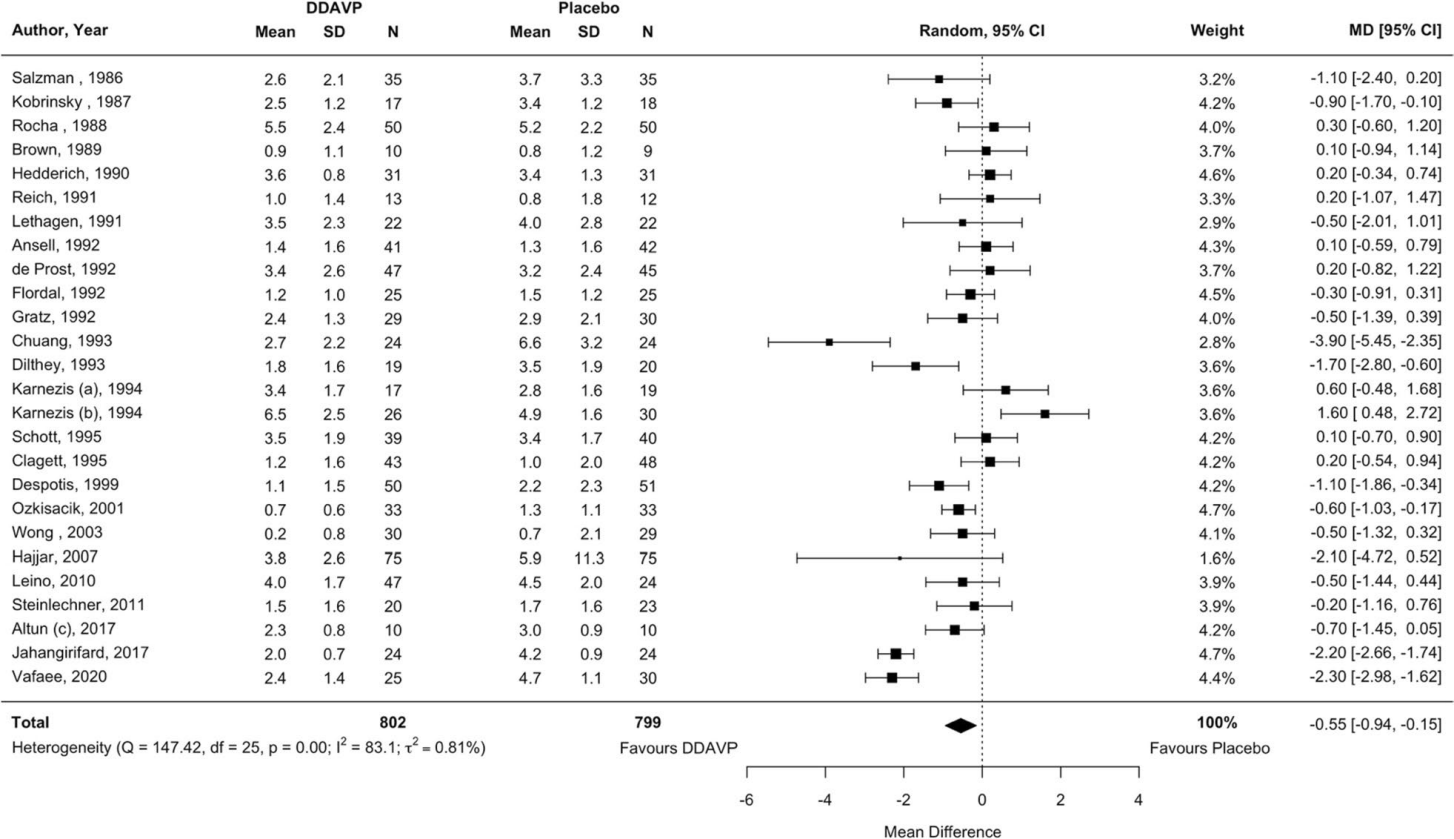
Comparison of desmopressin to placebo or usual care examining the outcome of units of red blood cell transfusion

Compared with placebo or usual care, desmopressin (Steinlechner et al. 2011; Schött et al. 1995; Salzman et al. 1986; Rocha et al. 1988; Reynolds et al. 1993; Reich et al. 1991; Ozkisacik et al. 2001; Lethagen et al. 1991; Leino et al. 2010; Kobrinsky et al. 1987; Karnezis et al. 1994; Hedderich et al. 1990; Hajjar et al. 1853; Gratz et al. 1992; Flordal et al. 1992; Dilthey et al. 1993; Despotis et al. 1999; Prost et al. 1992; Clagett et al. 1995; Chuang et al. 1993; Brown et al. 1989; Ansell et al. 1992; Altun et al. 2017; Jahangirifard et al. 2017; (Vafaee M, Alizadeh A, Gholamian A: Evaluation of preoperative intravenous desmopressin on blood loss in major spine surgery, unpublished)) attenuated blood transfusion requirements by 0.55 units compared with placebo (25 studies, 26 comparisons (Wong et al. 2003; Steinlechner et al. 2011; Schött et al. 1995; Salzman et al. 1986; Rocha et al. 1988; Reich et al. 1991; Ozkisacik et al. 2001; Lethagen et al. 1991; Leino et al. 2010; Kobrinsky et al. 1987; Karnezis et al. 1984; Hedderich et al. 1990; Hajjar et al. 1853; Gratz et al. 1992; Flordal et al. 1992; Dilthey et al. 1993; Despotis et al. 1999; Prost et al. 1992; Clagett et al. 1995; Chuang et al. 1993; Brown et al. 1989; Ansell et al. 1992; Altun et al. 2017; Jahangirifard et al. 2017; (Vafaee M, Alizadeh A, Gholamian A: Evaluation of preoperative intravenous desmopressin on blood loss in major spine surgery, unpublished)), *n* = 1601 participants, MD − 0.55 units, 95% CI − 0.94 to − 0.15 units, *I*^2^ = 83.1%, *p* = 0.007; Table 1 and Fig. 4). This analysis was conducted after the exclusion of a study,(Reynolds et al. 1993) which reported transfusion volume in ml/kg rather than units. Based on TSA, we do not have sufficient evidence to confidently conclude this hemostatic effect of desmopressin (Table 1, Additional file 1: Figure S8). We did not identify significant subgroup effects to explain the detected statistical heterogeneity (Table 1). Of note, the subgroup analysis suggested that individuals who underwent cardiac surgery, compared to non-cardiac surgery, experienced more benefit in reduction in transfusion volume with desmopressin. Both adult and pediatric participants who received desmopressin had attenuated transfusion volumes.

#### Comparison to TXA

There was insufficient evidence that the transfusion volumes differed amongst adults who received desmopressin compared with TXA based on our meta-analysis of two studies(Zohar et al. 2001; Altun et al. 2017) (MD 1.25, 95% CI − 0.03 to 2.52, *p* = 0.055, very low certainty, *I*^2^ = 91.6%). The number of studies precluded subgroup analyses. Additional studies are needed to inform the comparison of desmopressin to TXA with respect to transfusion requirements (Table 1, Additional file 1: Figure S9).

### Any bleeding

**Fig. 5:**
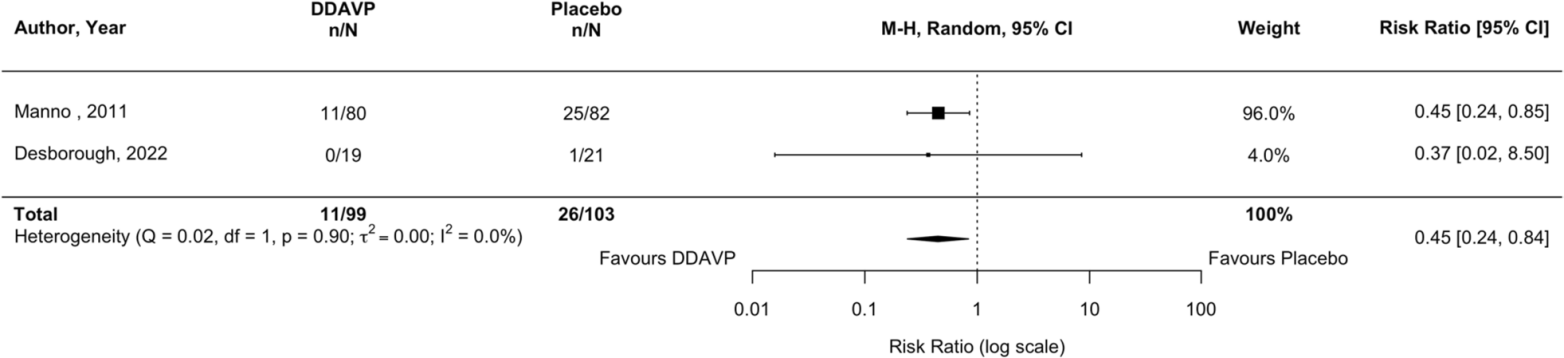
Comparison of desmopressin to placebo or usual care examining the outcome of any bleeding

Two studies (Manno et al. 2011; Desborough et al. 2022) reported on any bleeding (202 participants). Compared with placebo, desmopressin administration reduced the risk of any bleeding by 55% (RR 0.45, 95% CI, 0.24 to 0.84, *p* = 0.012, *I*^2^ = 0.0%; Fig. 5). The certainty of evidence pertaining to the effect estimate on any bleeding is low, owing to serious limitations of risk of bias and imprecision (Table 1). TSA suggests that more studies could potentially alter this finding (Table 1, Additional file 1: Figure S10).

### Reoperation due to bleeding

**Fig. 6:**
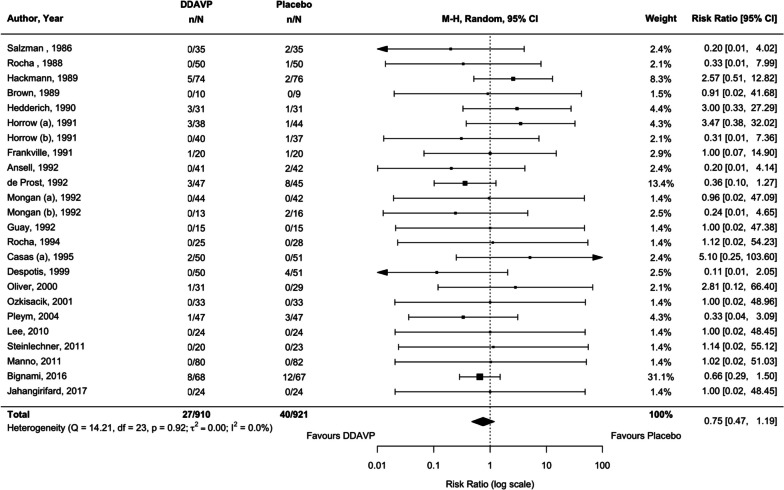
Comparison of desmopressin to placebo or usual care examining the outcome of reoperation due to bleeding

Our review identified 22 studies (24 comparisons, *n* = 1831) (Lee et al. 2010; Steinlechner et al. 2011; Salzman et al. 1986; Rocha et al. 1988, 1994; Pleym et al. 2004; Ozkisacik et al. 2001; Oliver et al. 2000; Mongan and Hosking 1992; Manno et al. 2011; Horrow et al. 1991; Hedderich et al. 1990; Hackmann et al. 1989; Guay et al. 1992; Frankville et al. 1991; Despotis et al. 1999; Prost et al. 1992; Casas et al. 1995; Brown et al. 1989; Bignami et al. 2016; Ansell et al. 1992; Jahangirifard et al. 2017) that compared the effect on reoperation due to bleeding of desmopressin to placebo or usual care and two studies (Casas et al. 1995; Rocha et al. 1994) that compared it to aprotinin. We found low certainty evidence that desmopressin (compared with placebo or usual care) may result in little to no difference in this outcome (RR 0.75, 95% CI 0.47 to 1.19, *p* = 0.223, *I*^2^ = 0.0%; Table 1, Fig. 6, Additional file 1: Figure S11). Given the width of the conference interval, there was insufficient evidence to meaningfully compare desmopressin and aprotinin in their effects on this outcome (RR 1.36, 95% CI 0.18 to 10.29).

### Non-hemostatic outcomes

**Fig. 7:**
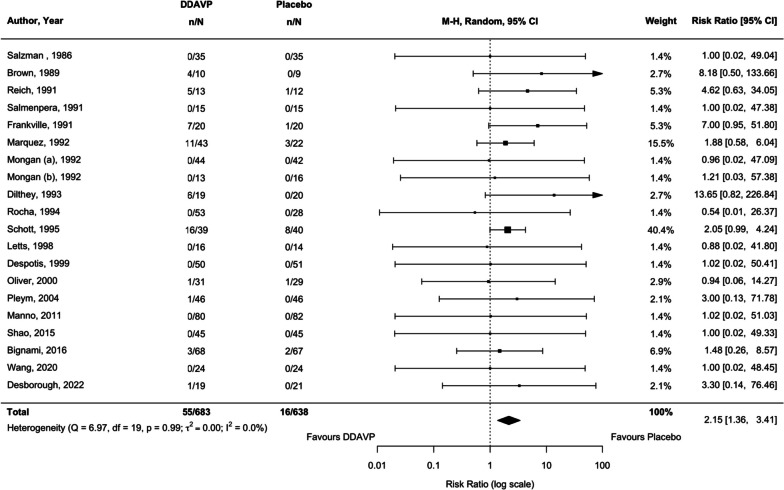
Comparison of desmopressin to placebo or usual care examining the outcome of clinically important hypotension

Based on 19 studies (20 comparisons) (Shao et al. 2015; Schött et al. 1995; Salzman et al. 1986; Salmenperä et al. 1991; Reich et al. 1991; Pleym et al. 2004; Oliver et al. 2000; Mongan and Hosking 1992; Marquez et al. 1992; Manno et al. 2011; Letts et al. 1998; Frankville et al. 1991; Dilthey et al. 1993; Despotis et al. 1999; Brown et al. 1989; Bignami et al. 2016; Wang et al. 2020; Desborough et al. 2022; Rocha et al. 1994), periprocedural desmopressin administration increased the risk of clinically important hypotension by 2.15-fold compared with placebo or usual care (RR 2.15, 95% CI 1.36 to 3.41, *p* = 0.001, moderate certainty, *I*^2^ = 0.0%; Fig. 7). We noted a trend towards a greater increase in risk of clinically important hypotension amongst adults than among children. Studies published in or after 2012 observed a greater increase in the incidence of hypotension with desmopressin administration than those published prior to 2012 (Table 1).

We found no significant differences in the risk of myocardial infarction, stroke, venous thromboembolism, and hyponatremia following desmopressin administration. We did not find significant differences in the thrombotic complications between desmopressin versus aprotinin, although this comparison is limited to very low-certainty evidence (Table 1).

Only one trial (Wang et al. 2020) assessed the effects of desmopressin on nausea, but none of the participants experienced the outcome. No participants experienced seizures in the single trial that examined this (Oliver et al. 2000). This study included pediatric participants ranging from < 2 to 18 years old in the following distributions: < 2 years (9.7%), 3 to < 10 years (35.5%), and 10 to < 18 years (32%) in the desmopressin group (*n* = 31). None of the participants in the desmopressin and placebo groups experienced seizures as a perioperative complication (Oliver et al. 2000). Finally, no studies were reported on facial flushing or peripheral arterial thrombosis.

### Baseline kidney function

Seven studies (Pleym et al. 2004; Manno et al. 2011; Leino et al. 2010; Clagett et al. 1995; Bignami et al. 2016; Altun et al. 2017; (Vafaee M, Alizadeh A, Gholamian A: Evaluation of preoperative intravenous desmopressin on blood loss in major spine surgery, unpublished)) reported summaries of baseline kidney function. Reporting was too heterogeneous for subgroup analysis with some studies reporting means and standard deviations of serum creatinine while others reported the number of participants with creatinine ≥ 2 g/dL (177 μmol/L) (Additional file 1: Table S3). Few patients, if any, would have had severe kidney disease based on these summaries.

## Discussion

Overall, our review suggests that—compared to placebo or usual care—desmopressin reduces periprocedural bleeding. Specifically, desmopressin reduced blood loss, number of units of red blood cells transfused, and the risk of investigator-defined bleeding. However, it did not mitigate the risk of receiving any blood transfusion. Desmopressin appears to have less potent hemostatic effects than TXA. Desmopressin led to a two-fold increase in the risk of clinically important hypotension, but did not precipitate hyponatremia and thromboembolic complications. Our conclusions regarding these safety events are drawn with caution due to their low event rates.

Our findings remain consistent with those of previous reviews(Desborough et al. 2017; Carless et al. 2004; Crescenzi et al. 2008; Laupacis and Fergusson 1997; Levi et al. 1999), including the 2017 Cochrane review, and were unaffected by practice recommendations that favor a restrictive transfusion strategy.

Patient blood management strategies, which encompass optimization of erythropoiesis, reduce periprocedural bleeding, and optimize patient’s tolerance of anemia, are endorsed by the World Health Organization (Desai et al. 2018; Maryuningsih et al. 2023; Zacharowski et al. 2022). Desmopressin is one of several strategies to mitigate the risk of periprocedural bleeding. For example, the preoperative administration of erythropoietin and iron to anemic patients undergoing non-cardiac surgery reduced transfusion requirements (RR 0.55, 95% CI 0.38 to 0.80) and increased hemoglobin concentration (MD 1.87 g/dL, 95% CI 1.26 to 2.49 g/dL) compared to placebo (Kaufner et al. 2020). Furthermore, TXA reduced the perioperative requirement for blood transfusions and mortality by 38% (RR 0.62, 95% CI 0.58 to 0.65) and 39% (RR 0.61, 95% CI 0.38 to 0.98), respectively, in patients undergoing both cardiac and non-cardiac surgeries (Ker et al. 2012). Our review suggested that while desmopressin is likely a less effective hemostatic agent compared with TXA, it may be an additional strategy to further reduce bleeding and transfusion.

Mechanistically, there may be additive and potentially synergistic therapeutic effects in combining TXA and desmopressin. Desmopressin promotes the release of tissue plasminogen activator, which activates fibrinolysis that TXA may then inhibit and lead to an even greater hemostatic effect than expected from the addition the each agent’s independent effects (Ozgonenel et al. 2007; Ozal et al. 2002). A small RCT of 100 patients who underwent coronary artery bypass surgery found that the coadministration of desmopressin and TXA reduced transfusion requirements when compared to the combination of desmopressin and protamine,(Ozal et al. 2002) but the potential synergy between desmopressin and TXA could not be evaluated in the absence of groups treated with TXA alone and desmopressin alone. These questions are best assessed in trials with a factorial design.

It is possible that desmopressin reduces transfusion costs in some patients. While desmopressin did not avert the need for blood transfusion in these trials, it reduced the units of blood transfused by 0.55 units (95% CI, 0.15 to 0.94 unit reduction). The cost of inpatient administration of a unit of red blood cells in Canada was estimated at $243.10 in 2017 (Lagerquist et al. 2017). Intravenous desmopressin solution costs between $51.75 and $88.05 CAD for 20 μg (personal communication with hospital pharmacy in Ontario in 2023). Blood products also bring a spectrum of transfusion side effects, including transfusion reactions, infections, lung injury, and others (Callum et al. 2022). However, desmopressin can lead to perioperative hypotension, which associates with organ injury (Walsh et al. 2013; Roshanov et al. 2019, 2017). Therefore, the use of desmopressin for reducing perioperative bleeding should be accompanied by strategies to reduce the risk of post-administration hypotension. Specifically, administering intravenous desmopressin over 10 to 30 min can minimize the risk of hypotension (Goad and Levesque 2020). The prothrombotic potential of desmopressin remains unclear (Desborough et al. 2017; Carless et al. 2004; Crescenzi et al. 2008; Levi et al. 1999). Levi and colleagues (Levi et al. 1999) reported an increased risk of perioperative myocardial infarction associated with desmopressin administration, but this finding was not replicated in our review. Finally, we did not observe an increased risk of hyponatremia following desmopressin administration, which can occur due to its antidiuretic properties and may be exacerbated by unrestricted fluid intake (Svensson et al. 2014).

There are several knowledge gaps. First, there was an overall dearth of reporting of adverse effects in clinical trials of desmopressin. Second, most included studies did not capture participants with baseline thrombocytopenia or coagulopathy (Desborough et al. 2017). Third, most trials examined desmopressin in adults undergoing cardiac surgery. Fourth, we found no direct RCT evidence to evaluate periprocedural in patients with advanced kidney disease (Mannucci et al. 1983). Baseline kidney function was rarely reported and many RCTs have excluded people with chronic kidney disease altogether (Desborough et al. 2017; Shao et al. 2015; Leino et al. 2010; Flordal et al. 1992; Ellis et al. 2001). Therefore, the efficacy and safety of perioperative desmopressin administration in patients with severe kidney dysfunction remain unknown. Future studies may explore desmopressin in non-cardiac surgery, non-surgical procedures, and in patients with thrombocytopenia, coagulopathy, or kidney disease. Lastly, there was a paucity of clinical trials that examined the efficacy and safety of desmopressin in children in our review but we excluded trials in patients with congenital bleeding disorders (which may more often include children). Safety data applicable to children may also be found in trials evaluating desmopressin for nocturnal enuresis (Glazener and Evans 2002).

The results of our review should be interpreted in light of its limitations. First, we observed that desmopressin reduced transfusion volume but did not affect the need for transfusion, despite its efficacy in reducing blood loss. It is possible that desmopressin may not lead to a sufficient reduction in bleeding to reduce the risk of receiving at least one unit of blood in patients who go on to receive blood. Additionally, we did not find significant differences in the need for at least one blood transfusion across different types of procedures, although there was a trend towards a more pronounced reduction in transfusion volume in cardiac surgery—where transfusion volumes are larger—compared to non-cardiac surgery. However, most studies did not specify their transfusion thresholds. Second, our review primarily focused on intravenous desmopressin with only one study involving subcutaneous administration; we excluded oral and intranasal formulations. Third, inferences remain uncertain and estimates of effect are imprecise despite the inclusion of recently completed trials. The TSA suggested that sufficient information sizes have been accrued in the existing literature to inform the effects of desmopressin on the risk of transfusion, the volume of red cell transfusion, and any bleeding. However, the quality of evidence was often downgraded due to concerns about the risk of bias in the randomization process, potential deviations from the intended intervention, and selective reporting of outcomes across the eligible studies. Finally, most studies were published over 10 years ago and do not reflect contemporary practices for minimizing periprocedural bleeding and avoiding transfusion. Therefore, there is room for investigating desmopressin in high-quality RCTs alongside other modern strategies to reduce bleeding and transfusion.

## Conclusion

Overall, desmopressin reduced periprocedural bleeding and the volume of blood transfused in small trials with methodologic limitations. Larger contemporary trials that evaluate desmopressin alongside TXA and enroll patients with advanced kidney disease would inform guidelines on perioperative blood management.

### Supplementary Information

**Additional file 1:** Search Strategies. Rationale for ascertainment of review outcomes within 30 days of surgical or non-surgical procedure. Title and Abstract Screening Pilot Form. Deviations from previous review. Methodology applied to map risk of bias assessments. **Table S1.** Mapping of risk of bias assessment domains between Cochrane Risk of Bias 1.0 and 2.0 tools. **Table S2.** Characteristics of the included studies investigating hemostatic efficacy of desmopressin in surgical and non-surgical procedures.*Table S3. Individual studies that reported the baseline kidney function of participants. **Figure S1.** Risk of bias assessments for new studies using Cochrane Risk of Bias 2.0 criteria. **Figure S2.** Risk of bias assessments for studies included in the previous review, mapped using Cochrane Risk of Bias 2.0. **Figure S3.** Risk of bias of 2 randomly selected studies using Cochrane Risk of Bias 2.0. **Figure S4**. Risk of bias of 2 randomly selected studies applying mapping of Cochrane Risk of Bias 2.0. **Figure S5.** Trial sequential analysis of desmopressin compared with placebo or usual care on the number of participants needing red blood cell transfusion. **Figure S6.** Trial sequential analysis of desmopressin compared with placebo or usual care on total volume of blood loss. **Figure S7.** Trial sequential analysis of desmopressin compared with tranexamic acid on total volume of blood loss. **Figure S8.** Trial sequential analysis of desmopressin compared with placebo or usual care on units of red blood cells transfused. **Figure S9.** Trial sequential analysis of desmopressin compared with tranexamic acid on units of red blood cells transfused. **Figure S10.** Trial sequential analysis of desmopressin compared with placebo or usual care on any bleeding. **Figure S11.** Trial sequential analysis of desmopressin compared with placebo or usual care on reoperation due to bleeding. **Figure S12.** Funnel plot of desmopressin to placebo or usual care for outcome of number of participants who received a red cell transfusion amongst participants. **Figure S13.** Funnel plot of desmopressin to placebo or usual care for outcome of total volume of blood loss. **Figure S14.** Funnel plot of desmopressin to placebo or usual care examining the outcome of units of red blood cell transfusion. **Figure S15.** Funnel plot of desmopressin to placebo or usual care examining the outcome of any bleeding. **Figure S16.** Funnel plot of desmopressin to placebo or usual care examining the outcome of reoperation due to bleeding. **Figure S17.** Funnel plot of desmopressin to placebo or usual care examining the outcome of myocardial infarction. **Figure S18.** Funnel plot of desmopressin to placebo or usual care examining the outcome of stroke. **Figure S19.** Funnel plot of desmopressin to placebo or usual care examining the outcome of clinically important hypotension. **Figure S20.** Funnel plot of desmopressin to placebo or usual care examining the outcome of venous thromboembolism. **Figure S21.** Funnel plot of desmopressin to placebo or usual care examining the outcome of hyponatremia (dichotomous). **Figure S22.** Funnel plot of desmopressin to placebo or usual care examining the outcome of post-procedural serum sodium. **Figure S23.** Funnel plot of desmopressin to tranexamic acid for outcome of number of participants who received a red cell transfusion amongst participants. **Figure S24.** Funnel plot of desmopressin to tranexamic acid for outcome of total volume of blood loss. **Figure S25.** Funnel plot of desmopressin to tranexamic acid examining the outcome of units of red blood cell transfusion. **Figure S26.** Funnel plot of desmopressin to aprotinin examining the outcome of reoperation due to bleeding. **Figure S27.** Funnel plot of desmopressin to aprotinin examining the outcome of myocardial infarction. **Figure S28.** Funnel plot of desmopressin to aprotinin examining the outcome of stroke. **Figure S29.** Funnel plot of desmopressin to aprotinin examining the outcome of venous thromboembolism. Summary of characteristics of 15 studies that meet eligibility criteria but were available in the form of trial registries or abstracts that did not provide relevant data.

## Data Availability

The data used and/or analyzed during the current study are available from the corresponding author on reasonable request.

## Abbreviations

AABB: American Association of Blood Banks
CENTRAL: Cochrane Central Register of Controlled Trials
CI: Confidence interval
CINAHL: Cumulative Index to Nursing and Allied Health Literature
eGFR: Estimated glomerular filtration rate
GRADE: Grading of Recommendations, Assessment, Development and Evaluation
LILACS: Latin American and Caribbean Health Sciences Literature
MD: Mean difference
PRISMA: Preferred Reporting Items for Systematic Reviews and Meta-Analyses
RBC: Red blood cell
RCT: Randomized controlled trials
RoB: Risk of bias
RR: Risk ratio
SD: Standard deviation
SMD: Standardized mean difference
TSA: Trial sequential analysis
TXA: Tranexamic acid
vWF: Von Willebrand factor
