# Supplementary material for: Desmopressin to reduce periprocedural bleeding and transfusion: a systematic review and meta-analysis
Source: Perioper Med (Lond). 2024 Jan 23;13:5. doi: 10.1186/s13741-023-00358-4 (PMC10804695; doi:10.1186/s13741-023-00358-4)
Supplement: Supplementary file 1 — Additional file 1: Search Strategies. Rationale for ascertainment of review outcomes within 30 days of surgical or non-surgical procedure. Title and Abstract Screening Pilot Form. Deviations from previous review. Methodology applied to map risk of bias assessments. Table S1. Mapping of risk of bias assessment domains between Cochrane Risk of Bias 1.0 and 2.0 tools. Table S2. Characteristics of the included studies investigating hemostatic efficacy of desmopressin in surgical and non-surgical procedures.*Table S3. Individual studies that reported the baseline kidney function of participants. Figure S1. Risk of bias assessments for new studies using Cochrane Risk of Bias 2.0 criteria. Figure S2. Risk of bias assessments for studies included in the previous review, mapped using Cochrane Risk of Bias 2.0. Figure S3. Risk of bias of 2 randomly selected studies using Cochrane Risk of Bias 2.0. Figure S4. Risk of bias of 2 randomly selected studies applying mapping of Cochrane Risk of Bias 2.0. Figure S5. Trial sequential analysis of desmopressin compared with placebo or usual care on the number of participants needing red blood cell transfusion. Figure S6. Trial sequential analysis of desmopressin compared with placebo or usual care on total volume of blood loss. Figure S7. Trial sequential analysis of desmopressin compared with tranexamic acid on total volume of blood loss. Figure S8. Trial sequential analysis of desmopressin compared with placebo or usual care on units of red blood cells transfused. Figure S9. Trial sequential analysis of desmopressin compared with tranexamic acid on units of red blood cells transfused. Figure S10. Trial sequential analysis of desmopressin compared with placebo or usual care on any bleeding. Figure S11. Trial sequential analysis of desmopressin compared with placebo or usual care on reoperation due to bleeding. Figure S12. Funnel plot of desmopressin to placebo or usual care for outcome of number of participants who received a red cell [file 13741_2023_358_MOESM1_ESM.docx]

**Additional file 1**

for

Desmopressin to reduce periprocedural bleeding and transfusion: a systematic review and meta-analysis.

Wang et al.

Table of Contents

[Search Strategies 4](#_Toc153303281)

[Rationale for ascertainment of review outcomes within 30 days of surgical or non-surgical procedure 12](#_Toc153303282)

[Title and Abstract Screening Pilot Form 13](#_Toc153303283)

[Deviations from previous review 14](#_Toc153303284)

[Methodology applied to map risk of bias assessments 16](#_Toc153303285)

[Additional Tables 17](#_Toc153303286)

[Table S1. Mapping of risk of bias assessment domains between Cochrane Risk of Bias 1.0 and 2.0 tools. 17](#_Toc153303287)

[Table S2. Characteristics of the included studies investigating hemostatic efficacy of desmopressin in surgical and non-surgical procedures.* 18](#_Toc153303288)

[Table S3. Individual studies that reported the baseline kidney function of participants. 39](#_Toc153303289)

[Additional Figures 40](#_Toc153303290)

[Figure S1. Risk of bias assessments for new studies using Cochrane Risk of Bias 2.0 criteria. 40](#_Toc153303291)

[Figure S2. Risk of bias assessments for studies included in the previous review, mapped using Cochrane Risk of Bias 2.0. 43](#_Toc153303292)

[Figure S3. Risk of bias of 2 randomly selected studies using Cochrane Risk of Bias 2.0. 44](#_Toc153303293)

[Figure S4. Risk of bias of 2 randomly selected studies applying mapping of Cochrane Risk of Bias 2.0. 45](#_Toc153303294)

[Figure S5. Trial sequential analysis of desmopressin compared with placebo or usual care on the number of participants needing red blood cell transfusion. 46](#_Toc153303295)

[Figure S6. Trial sequential analysis of desmopressin compared with placebo or usual care on total volume of blood loss. 47](#_Toc153303296)

[Figure S7. Trial sequential analysis of desmopressin compared with tranexamic acid on total volume of blood loss. 48](#_Toc153303297)

[Figure S8. Trial sequential analysis of desmopressin compared with placebo or usual care on units of red blood cells transfused. 49](#_Toc153303298)

[Figure S9. Trial sequential analysis of desmopressin compared with tranexamic acid on units of red blood cells transfused. 50](#_Toc153303299)

[Figure S10. Trial sequential analysis of desmopressin compared with placebo or usual care on any bleeding. 51](#_Toc153303300)

[Figure S11. Trial sequential analysis of desmopressin compared with placebo or usual care on reoperation due to bleeding. 52](#_Toc153303301)

[Figure S12. Funnel plot of desmopressin to placebo or usual care for outcome of number of participants who received a red cell transfusion amongst participants. 53](#_Toc153303302)

[Figure S13. Funnel plot of desmopressin to placebo or usual care for outcome of total volume of blood loss. 54](#_Toc153303303)

[Figure S14. Funnel plot of desmopressin to placebo or usual care examining the outcome of units of red blood cell transfusion. 55](#_Toc153303304)

[Figure S15. Funnel plot of desmopressin to placebo or usual care examining the outcome of any bleeding. 56](#_Toc153303305)

[Figure S16. Funnel plot of desmopressin to placebo or usual care examining the outcome of reoperation due to bleeding. 57](#_Toc153303306)

[Figure S17. Funnel plot of desmopressin to placebo or usual care examining the outcome of myocardial infarction. 58](#_Toc153303307)

[Figure S18. Funnel plot of desmopressin to placebo or usual care examining the outcome of stroke. 59](#_Toc153303308)

[Figure S19. Funnel plot of desmopressin to placebo or usual care examining the outcome of clinically important hypotension. 60](#_Toc153303309)

[Figure S20. Funnel plot of desmopressin to placebo or usual care examining the outcome of venous thromboembolism. 61](#_Toc153303310)

[Figure S21. Funnel plot of desmopressin to placebo or usual care examining the outcome of hyponatremia (dichotomous). 62](#_Toc153303311)

[Figure S22. Funnel plot of desmopressin to placebo or usual care examining the outcome of post-procedural serum sodium. 63](#_Toc153303312)

[Figure S23. Funnel plot of desmopressin to tranexamic acid for outcome of number of participants who received a red cell transfusion amongst participants. 64](#_Toc153303313)

[Figure S24. Funnel plot of desmopressin to tranexamic acid for outcome of total volume of blood loss. 65](#_Toc153303314)

[Figure S25. Funnel plot of desmopressin to tranexamic acid examining the outcome of units of red blood cell transfusion. 66](#_Toc153303315)

[Figure S26. Funnel plot of desmopressin to aprotinin examining the outcome of reoperation due to bleeding. 67](#_Toc153303316)

[Figure S27. Funnel plot of desmopressin to aprotinin examining the outcome of myocardial infarction. 68](#_Toc153303317)

[Figure S28. Funnel plot of desmopressin to aprotinin examining the outcome of stroke. 69](#_Toc153303318)

[Figure S29. Funnel plot of desmopressin to aprotinin examining the outcome of venous thromboembolism. 70](#_Toc153303319)

[Summary of characteristics of 15 studies that meet eligibility criteria but were available in the form of trial registries or abstracts that did not provide relevant data. 71](#_Toc153303320)

# Search Strategies

Discussed and reviewed with Jack Young, McMaster Librarian

**MEDLINE via OVID**: search was limited to January 1, 2017 to February 1, 2023.

Number of references found = 269

Covidence removed 3 duplicates

Ovid MEDLINE(R) and Epub Ahead of Print, In-Process, In-Data-Review & Other Non-Indexed Citations, Daily and Versions <1946 to January 31, 2023>

1 Deamino Arginine Vasopressin/ 4364

2 (desmopressin* or deamino* or desamino* or adiuretin* or stimate or desmotabs or D-void or octim or octostim or minurin* or minirin* or minrin or desurin or desmospray or defirin or concentraid or desmotab* or desmogalen or presinex or nocutil or noctisson).tw. 5975

3 (DDAVP or DDVAP).tw. 2415

4 1 or 2 or 3 7771

5 Meta-Analysis.pt. 174941

6 ((meta analy* or metaanaly*) and (trials or studies)).ab. 199521

7 (meta analy* or metaanaly* or evidence-based).ti. 205988

8 ((systematic* or evidence-based) adj2 (review* or overview*)).tw. 299912

9 (cochrane or embase or cinahl or cinhal or lilacs or citation index or psyclit or psychlit or psycinfo or psychinfo or "web of science" or scopus).ab. 264540

10 Cochrane Database of systematic reviews.jn. 16125

11 ((literature or systematic* or comprehensive* or electronic*) adj2 search*).ab. 146406

12 (additional adj (papers or articles or sources)).ab. 3226

13 (bibliograph* or handsearch* or hand search* or manual* search* or searched or reference list*).ab. 216289

14 (relevant adj (journals or articles)).ab. 16614

15 or/5-14 630299

16 Review.pt. 3116724

17 RANDOMIZED CONTROLLED TRIALS AS TOPIC/ 160174

18 selection criteria.ab. or critical appraisal.ti. 38950

19 (data adj (extraction or analys*)).ab. 128535

20 RANDOMIZED CONTROLLED TRIALS/ 160174

21 or/17-20 307048

22 16 and 21 95872

23 15 or 22 671017

24 randomized controlled trial.pt. 585694

25 controlled clinical trial.pt. 95174

26 randomi*.tw. 785423

27 placebo.ab. 235722

28 clinical trials as topic.sh. 200844

29 rando mLy.ab. 1

30 groups.ab. 2477029

31 trial.tw. 737448

32 or/24-31 3630600

33 23 or 32 4084518

34 (ANIMALS/ or exp ANIMAL EXPERIMENTATION/ or exp MODELS, ANIMAL/) not HUMANS/ 5057782

35 (Comment or Editorial).pt. 1430895

36 34 or 35 6439513

37 33 not 36 3547394

38 4 and 37 1130

39 ("2017*" or "2018*" or "2019*" or "202*").dt,ez,da. 9363266

40 38 and 39 269

**CENTRAL**, **the Cochrane Library**: search limited to January 1, 2017 to February 1, 2023

366 trials identified (974 identified with initial search strategy prior to date limitations), 72 duplicates removed, 294 remaining for title/abstract screening

ID Search Hits

#1 desmopressin* or deamino* or desamino* or adiuretin* or stimate or desmotabs or "D-void" or octim or octostim or minurin* or minirin*

or minrin or desurin or desmospray or defirin or concentraid or desmotab* or desmogalen or presinex or nocutil or noctisson 1020

#2 DDAVP or DDVAP 333

#3 MeSH descriptor: [Deamino Arginine Vasopressin] explode all trees 445

#4 #1 or #2 or #3 1083

**EMBASE via OVID**: search limited to January 1, 2017 to February 1, 2023

489 citations identified (1779 prior to date limitations), 133 duplicates removed, 356 studies remaining for title/abstract screening

Embase <1974 to 2023 January 31>

1 desmopressin acetate/ or desmopressin/ or desmopressin diacetate/ or "argipressin[1 deamino]"/ 14729

2 (desmopressin* or deamino* or desamino* or adiuretin* or stimate or desmotabs or D-void or octim or octostim or minurin* or minirin* or minrin or desurin or desmospray or defirin or concentraid or desmotab* or desmogalen or presinex or nocutil or noctisson).tw. 8417

3 (DDAVP or DDVAP).tw. 3716

4 1 or 2 or 3 16953

5 Meta Analysis/ 275889

6 Systematic Review/ 406353

7 (meta analy* or metaanalys*).tw. 335028

8 (systematic adj2 (review* or overview* or search*)).tw. 375511

9 (literature adj2 (review* or overview* or search*)).ti,ab. 352613

10 (cochrane or embase or cinahl or cinhal or lilacs or BIDS or science citation index or psyclit or psychlit or psycinfo or psychinfo or cancerlit).ti,ab. 285487

11 (electronic* adj (sources or resources or databases)).ab. 42591

12 reference lists.ab. 23292

13 (bibliograph* or handsearch* or hand search* or manual* search*).ab. 47451

14 (hand-search* or handsearch*).ab. 13341

15 (additional adj (papers or articles or sources)).ab. 3931

16 (relevant adj (journals or articles)).ab. 20570

17 (search term* or published articles or search strateg*).ab. 61661

18 or/5-17 979664

19 data extraction.ab. 37944

20 selection criteria.ab. 43846

21 or/19-20 79182

22 review.pt. 3007429

23 21 and 22 36548

24 editorial.pt. 753285

25 18 or 23 983329

26 25 not 24 978535

27 Controlled Clinical Trial/ or Phase 3 Clinical Trial/ or Phase 4 Clinical Trial/ 526954

28 Randomized Controlled Trial/ 759524

29 Randomization/ 97678

30 Single Blind Procedure/ 49801

31 Double Blind Procedure/ 204788

32 Crossover Procedure/ 73212

33 Placebo/ 395618

34 (randomized or randomised).tw. 1108132

35 RCT.tw. 51208

36 (random* adj5 (allocat* or assign* or divid* or receiv*)).tw. 433013

37 single blind*.tw. 30659

38 double blind*.tw. 239946

39 ((treble or triple) adj blind*).tw. 1751

40 (phase III or phase three or "phase 3").ti,ab. 125954

41 (crossover* or cross over* or cross-over* or placebo*).tw. 448027

42 Prospective Study/ 834977

43 or/27-42 2728503

44 Case Study/ 94978

45 case report*.tw. 609320

46 (note or editorial).pt. 1668747

47 or/44-46 2360799

48 43 not 47 2657108

49 26 or 48 3408371

50 limit 49 to embase 2079713

51 4 and 50 1779

52 limit 51 to dc=20170101-20230201 489

**PUBMED:** limited search to January 1, 2017 to February 1, 2023

81 results, 44 duplicates removed, 37 remaining for title/abstract screening

Search: **((desmopressin* OR deamino* OR desamino* OR adiuretin* OR stimate OR desmotabs OR "D-void" OR octim OR octostim OR minurin* OR minirin* OR minrin OR desurin OR desmospray OR defirin OR concentraid OR desmotab* OR desmogalen OR presinex OR nocutil OR noctisson OR DDAVP OR DDVAP) AND (random* OR blind* OR "control group" OR placebo* OR controlled OR groups OR trial* OR "systematic review" OR "meta-analysis" OR metaanalysis OR "literature search" OR medline OR cochrane OR embase)) AND (publisher[sb] OR inprocess[sb] OR pubmednotmedline[sb]) AND ("2017/01/01"[CRDT] : "3000"[CRDT] OR "2017/01/01"[EDAT] : "3000"[EDAT] OR "2017/01/01"[MHDA] : "3000"[MHDA])**

**CINAHL (ESBSCOHost): limited search to January 1, 2017 to February 1, 2023**

**69 results, 45 duplicates removed, 24 remaining studies for title/abstract screening**

| S46 | S44 AND S45 |
| --- | --- |
| S45 | EM 20170101-20230201 OR ZD "in process" |
| S44 | S26 AND S43 |
| S43 | S27 or S28 or S29 or S30 or S31 or S32 or S33 or S34 or S35 or S36 or S37 or S38 or S39 or S40 or S41 or S42 |
| S42 | MH QUANTITATIVE STUDIES |
| S41 | TI placebo* OR AB placebo* |
| S40 | TI (cochrane OR embase OR cinahl OR cinhal OR lilacs OR BIDS OR science AND citation AND index OR cancerlit) OR AB (cochrane OR embase OR cinahl OR cinhal OR lilacs OR BIDS OR science AND citation AND index OR cancerlit) |
| S39 | TI ("literature review" OR "literature overview" OR "literature search*") OR AB ("literature review" OR "literature overview" OR "literature search*") |
| S38 | TI ("meta analys*" OR metaanalys* OR "systematic review" OR "systematic overview" OR "systematic search*") OR AB ("meta analys*" OR metaanalys* OR "systematic review" OR "systematic overview" OR "systematic search*") |
| S37 | MH SYSTEMATIC REVIEW |
| S36 | MH META ANALYSIS |
| S35 | MH PLACEBOS |
| S34 | ( TI (random* N2 (assign* or allocat*)) ) OR ( AB (random* N2 (assign* or allocat*)) ) |
| S33 | TI ((phase three) or (phase III) or (phase three)) or AB ((phase three) or (phase III) or (phase three)) |
| S32 | MH RANDOM ASSIGNMENT |
| S31 | TI randomi* OR AB randomi* |
| S30 | TI ((singl* blind*) OR (doubl* blind*) OR (trebl* blind*) OR (tripl* blind*) OR (singl* mask*) OR (doubl* mask*) OR (tripl* mask*)) OR AB ((singl* blind*) OR (doubl* blind*) OR (trebl* blind*) OR (tripl* blind*) OR (singl* mask*) OR (doubl* mask*) OR (tripl* mask*)) |
| S29 | TI ((controlled trial*) or (clinical trial*)) OR AB ((controlled trial*) or (clinical trial*)) |
| S28 | PT Clinical Trial |
| S27 | MH Clinical Trials+ |
| S26 | S24 OR S25 |
| S25 | TI ( desmopressin* or deamino* or desamino* or adiuretin* or stimate or desmotabs or D-void or octim or octostim or minurin* or minirin* or minrin or desurin or desmospray or defirin or concentraid or desmotab* or desmogalen or presinex or nocutil or noctisson or DDVAP or DDAVP ) OR AB ( desmopressin* or deamino* or desamino* or adiuretin* or stimate or desmotabs or D-void or octim or octostim or minurin* or minirin* or minrin or desurin or desmospray or defirin or concentraid or desmotab* or desmogalen or presinex or nocutil or noctisson or DDVAP or DDAVP ) |
| S24 | MH "Desmopressin" |
| S23 | S21 AND S22 |
| S22 | EM 20170101-20230201 OR ZD "in process" |
| S21 | S3 AND S20 |
| S20 | S4 or S5 or S6 or S7 or S8 or S9 or S10 or S11 or S12 or S13 or S14 or S15 or S16 or S17 or S18 or S19 |
| S19 | MH QUANTITATIVE STUDIES |
| S18 | TI placebo* OR AB placebo* |
| S17 | TI (cochrane OR embase OR cinahl OR cinhal OR lilacs OR BIDS OR science AND citation AND index OR cancerlit) OR AB (cochrane OR embase OR cinahl OR cinhal OR lilacs OR BIDS OR science AND citation AND index OR cancerlit) |
| S16 | TI ("literature review" OR "literature overview" OR "literature search*") OR AB ("literature review" OR "literature overview" OR "literature search*") |
| S15 | TI ("meta analys*" OR metaanalys* OR "systematic review" OR "systematic overview" OR "systematic search*") OR AB ("meta analys*" OR metaanalys* OR "systematic review" OR "systematic overview" OR "systematic search*") |
| S14 | MH SYSTEMATIC REVIEW |
| S13 | MH META ANALYSIS |
| S12 | MH PLACEBOS |
| S11 | ( TI (random* N2 (assign* or allocat*)) ) OR ( AB (random* N2 (assign* or allocat*)) ) |
| S10 | TI ((phase three) or (phase III) or (phase three)) or AB ((phase three) or (phase III) or (phase three)) |
| S9 | MH RANDOM ASSIGNMENT |
| S8 | TI randomi* OR AB randomi* |
| S7 | TI ((singl* blind*) OR (doubl* blind*) OR (trebl* blind*) OR (tripl* blind*) OR (singl* mask*) OR (doubl* mask*) OR (tripl* mask*)) OR AB ((singl* blind*) OR (doubl* blind*) OR (trebl* blind*) OR (tripl* blind*) OR (singl* mask*) OR (doubl* mask*) OR (tripl* mask*)) |
| S6 | TI ((controlled trial*) or (clinical trial*)) OR AB ((controlled trial*) or (clinical trial*)) |
| S5 | PT Clinical Trial |
| S4 | MH Clinical Trials+ |
| S3 | S1 OR S2 |
| S2 | TI ( desmopressin* or deamino* or desamino* or adiuretin* or stimate or desmotabs or D-void or octim or octostim or minurin* or minirin* or minrin or desurin or desmospray or defirin or concentraid or desmotab* or desmogalen or presinex or nocutil or noctisson or DDVAP or DDAVP ) OR AB ( desmopressin* or deamino* or desamino* or adiuretin* or stimate or desmotabs or D-void or octim or octostim or minurin* or minirin* or minrin or desurin or desmospray or defirin or concentraid or desmotab* or desmogalen or presinex or nocutil or noctisson or DDVAP or DDAVP ) |
| S1 | MH "Desmopressin" |

**Transfusion Evidence Library**

Clinical specialty: Surgery

Limitation (could only select date limitations to publication year, reviewed with Jack Young librarian): 2017-2023 = 13 studies – 11 duplicates removed, 2 remaining studies for title/abstract screening

Search: desmopressin OR deamino OR desamino OR adiuretin OR stimate OR desmotabs OR D-void OR octim OR octostim OR minurin OR minirin OR minrin OR desurin OR desmospray OR defirin OR concentraid OR desmotab OR desmogalen OR presinex OR nocutil OR noctisson OR DDAVP or DDVAP

Date restrictions: ‘Published between’ 2017 to 2023

**Web of Science CPCI-S**

Limitations: date limited to January 1, 2017 to Feb 1, 2023 = 212 results – 137 duplicates removed, 75 studies remaining for title/abstract screening

Search**: ((LD=(2017-01-01/2023-02-01)) AND ALL=(desmopressin OR deamino OR desamino OR adiuretin OR stimate OR desmotabs OR D-void OR octim OR octostim OR minurin OR minirin OR minrin OR desurin OR desmospray OR defirin OR concentraid OR desmotab OR desmogalen OR presinex OR nocutil OR noctisson OR DDAVP or DDVAP)) AND ALL=(((randomi* OR rando mLy OR "random assignment" OR "random allocation" OR blind* OR "control group*" OR "controlled trial" OR "controlled study")))**

**LILACS**

Limitations (could only limit by publication year range): 2017-2023 – 4 results, 2 duplicates removed, 2 remaining for title/abstract screening

tw:((desmopressin* OR deamino* OR desamino* OR adiuretin* OR stimate OR desmotabs OR "D-void" OR octim OR octostim OR minurin* OR minirin* OR minrin OR desurin OR desmospray OR defirin OR concentraid OR desmotab* OR desmogalen OR presinex OR nocutil OR noctisson OR ddavp OR ddvap) ) AND (instance:"regional") AND ( db:("LILACS") AND type_of_study:("clinical_trials"))

**KoreaMed**

Limitations (could only limit by publication year range): 2017-2023

7 studies, 2 duplicates removed, 5 remaining for title/abstract screening

Search: (("Deamino Arginine Vasopressin"[ALL])) OR ("desmopressin"[ALL]) AND ("Randomized Controlled Trial"[ALL])

- Search strategy was slightly modified with Jack Young librarian, as attempts to replicate the search strategy from Desborough et al, 2017 did not yield relevant results

**PakMediNet**

Limitation (by publication year): 2017 to 2023

1 study (initially 6 studies, but others published between 2002 to 2014) – no duplicates, 1 remaining for title/abstract screening

Search: Desmopressin OR DDAVP

**HKU Clinical Trials Registry**

Search Terms: Desmopressin

Study Type: Interventional

Date of study registration: January 1, 2017 to February 1, 2023 – no studies yielded (total 3 studies found when date limits removed, ranged between 2009 to 2011)

*A search in IndMed could not be completed due to lack of access. We did not perform literature searches in Clinicaltrials.gov and WHO ICTRP because their contents are captured in the CENTRAL search.*

# Rationale for ascertainment of review outcomes within 30 days of surgical or non-surgical procedure

Intravenous and subcutaneous DDAVP reach peak activity at 30-60 minutes and 60-90 minutes post-administration, respectively.[1] The hemostatic effects of DDAVP range from 6 to 8 hours.[1] Thus, we expect its therapeutic effects on procedural bleeding risks to be most notable during and in the immediate post-operative period of the procedure. Majority of randomized controlled trials performed their bleeding outcome assessments within 48 hours of the procedure.[2] Our evaluation of outcomes during or within 30 days of the procedure ensures the effects of DDAVP on bleeding risks are fully captured. In addition, examination of the 40,004 adults undergoing non-cardiac surgery enrolled in the VISION study found perioperative complications of major bleeding (adjusted HR 2.6, 95% CI 2.2-3.1), MINS (adjusted HR 2.2, 95% CI 1.9-2.6), stroke (adjusted HR 3.7, 95% CI 2.5-5.7), and venous thromboembolism (adjusted HR 2.2, 95% CI 1.3-3.7) were independently associated with 30 day all-cause mortality.[3] Our evaluation of outcomes during or within 30 days of the procedure also aligns with that of previous systematic review.[2]

References:

1. Leissinger C, Carcao M, Gill JC, Journeycake J, Singleton T, Valentino L. Desmopressin (DDAVP) in the management of patients with congenital bleeding disorders. *Haemophilia : the official journal of the World Federation of Hemophilia* 2014; **20**: 158–67.

2. Desborough MJ, Oakland K, Brierley C et al. Desmopressin use for minimising perioperative blood transfusion. *Cochrane Database of Systematic Reviews* 2017; **2017**.

3. Vascular Events in Noncardiac Surgery Patients Cohort Evaluation (VISION) Study Investigators, Spence J, LeManach Y et al. Association between complications and death within 30 days after noncardiac surgery. *CMAJ : Canadian Medical Association journal = journal de l’Association medicale canadienne* 2019; **191**: E830–7.

# Title and Abstract Screening Pilot Form

*Criteria for title and abstract screening:*

Please follow this sequence of decision making when evaluating the eligibility of a title/abstract:

1. Is the article about humans?

- If no, exclude.
- If yes or unclear, go to next question.

2. Does the article describe patients undergoing surgery or interventional procedure (e.g., kidney biopsy, liver biopsy, endoscopy)?

- If no, exclude.
- If yes or unclear, go to next question.

3. Does the article compare desmopressin to any of: placebo, or to active comparator (e.g., another drug), or no other treatment?

- If no, exclude.
- If yes or unclear, go to next question.

4. Is this study a randomized controlled trial?

- If no, exclude.
- If yes or unclear, include for full text review.

5. Is the article about adults or children with inherited bleeding disorders such as hemophilia or von Willebrand disease?

- If yes, exclude.
- If no or unclear, go to next question.

6. If remain uncertain of whether to include or exclude the article at this stage, then include for full text review.

# Deviations from previous review

Unlike the previous review, we pooled studies of adult and pediatric participants in our meta-analyses. Subsequently, the subgroup effect of participant age on the outcomes of interest were examined as interaction terms. Although there is clinical heterogeneity pertaining to baseline bleeding risks across different procedures,[1] the relative effects of DDAVP on hemostatic outcomes were made comparable by examining the relative risks of dichotomous outcomes and standardized mean differences of continuous outcomes. Deviations in participant grouping in three of the included studies are summarized below:

Marquez 1992: This was a 3 parallel arm randomized controlled trial that included 3 cohorts of n=22 individuals who received 2 doses of desmopressin, n=21 individuals who received 1 dose of desmopressin and n=22 individuals who received placebo (normal saline). Desborough and colleagues reported only the comparison of single-dose DDAVP with placebo in their analysis.[1] In this review, we combined the two cohorts of participants who received desmopressin (2 doses and 1 dose) into a single intervention arm (n=43).

Leino 2010: This was a 3 parallel arm randomized controlled trial that included 3 cohorts of: n=24 individuals who received DDAVP 0.2mcg/kg, n=23 individuals who received DDAVP 0.4mcg/kg and n=24 individuals who received placebo (normal saline). Desborough et al[1] review reported only on the comparison of individuals who received the higher dose of 0.4mcg/kg with placebo in their analysis.[1] In this review, we combined the two cohorts of participants who received desmopressin (0.2mcg/kg and 0.4mcg/kg) into a single intervention arm (n=47).

Rocha 1994: This was a 4 parallel arm randomized controlled trial that included 4 cohorts of: n=25 individuals who received 1 dose of DDAVP, n=28 individuals who received 2 doses of DDAVP, n=28 individuals who received aprotinin, and n=28 individuals who received the standard usual care. Amongst the two cohorts of participants who received DDAVP, Desborough et al[1] review reported on the single dose DDAVP cohort. In this review, we combined the two DDAVP arms (2 doses and 1 dose) into a single intervention arm (n=53).

Reference:

1. Desborough MJ, Oakland K, Brierley C et al. Desmopressin use for minimising perioperative blood transfusion. *Cochrane Database of Systematic Reviews* 2017; **2017**.

# Methodology applied to map risk of bias assessments

The risk of bias of included studies in the previous review were evaluated using Cochrane Risk of Bias 1.0. In our update, we mapped the risk of bias assessments to the Cochrane Risk of Bias 2.0 (Additional Table S1). We also randomly selected 2 included studies to compare the risk of bias assessments per mapping technique compared with application of the Cochrane Risk of Bias 2.0. The two approaches generated comparable results for risk of bias evaluations (Additional Figures S1-S2).

# Additional Tables

## Table S1. Mapping of risk of bias assessment domains between Cochrane Risk of Bias 1.0 and 2.0 tools.

| Cochrane Risk of Bias 1.0 | Cochrane Risk of Bias 2.0 |
| --- | --- |
| Sequence generation | Randomization process |
| Allocation concealment |  |
| Blinding participants and personnel | Deviation from intended intervention |
| Blinding outcome assessors | Measurement of outcomes |
| Incomplete outcome data | Missing outcomes data |
| Selective outcome reporting | Selection of the reported result |

## Table S2. Characteristics of the included studies investigating hemostatic efficacy of desmopressin in surgical and non-surgical procedures.*

| Author  (Year of publication, County of study, Type of study) | Inclusion criteria | Exclusion criteria | Number of participants randomized (number of participants analyzed) | Mean age (years) | Females (%) | Type of procedure | Route and dose of desmopressin | Duration and Timing of desmopressin administration | Comparator | Study Outcomes relevant to current review | Length of follow up for outcome assessment (hours) |
| --- | --- | --- | --- | --- | --- | --- | --- | --- | --- | --- | --- |
| Altun  (2017)  Turkey  Single center, 4 arm parallel group RCT | Undergoing emergency CABG | ‘Chronic renal insufficiency, hepatic dysfunction, hematological disorders, drug addiction that might affect the hematological system, requirements for non-coronary cardiac surgery, or use of intra-aortic balloon pumps’ | 54 (54) | 63.9 | 13.0 | Cardiac | Intravenous, 0.3mcg/kg | 20 minutes, after heparin reversal | Usual care, tranexamic acid | Total volume of blood loss (total drainage from thoracic and mediastinal drains), volume of red cells transfused (transfusion protocol not specified) | Up to 24 hours of intensive care unit admission following surgery |
| Andersson (1990)  Sweden  Single center, 2 arm parallel group RCT | ‘CABG with 3 (or more) veins or internal mammary arterial bypass grafts’ | ‘Previous cardiac surgery; previous coagulation disorders; coumarin anticoagulants, heparin, or acetylsalicylic acid within 7 days before surgery’ | 100 (19) | 59.4 | 15.8 | Cardiac | Intravenous, 0.3mcg/kg | 15 minutes, administered 15 minutes after heparin reversal | Placebo (normal saline) | Total blood loss (measured by drain output) | During the first 10 hours postoperatively |
| Ansell  (1992)  USA  Multi-center, 2 arm parallel group RCT | ’Age 18 to 75 years; elective cardiac valve operations with, or without, coronary artery bypass’ | ‘Recent myocardial infarction (timing not specified); unstable angina; deep vein thrombosis or pulmonary embolism; history of bleeding diathesis or platelet defect; unstable haemodynamic status; pertinent drug allergy; pregnancy’ | 92 (83) | 61.0 | 45.8 | Cardiac | Intravenous, 0.3mcg/kg | 15 minutes, administered immediately after heparin reversal | Placebo (normal saline) | Total blood loss (measured by drain output), volume of red cell transfusions, number of participants receiving any red cell transfusion, reoperation due to bleeding, all-cause mortality, thromboembolic events | Up to 72 hours after operation |
| Bignami (2016)  Italy  Multi-center, 2 arm parallel group RCT | ‘Age ≥ 18 years; elective cardiac surgery; diffuse intraoperative bleeding without a surgical source or excessive postoperative bleeding from chest tubes defined as 100 mL over 30 minutes, or 2 mL/kg/h for at least 2 hours’ | ‘Lack of informed consent; myocardial infarction within previous 7 days’ | 135 (135) | 63.0 | 25.9 | Cardiac | Intravenous, 0.3mcg/kg | 20 minutes, administered in the event of excessive bleeding | Placebo (normal saline) | Total number of participants receiving a red cell transfusion, total volume of red cells transfused, total blood loss (measured by drain output; reported as median and interquartile range, so not included in meta-analysis), all-cause mortality, thrombotic events, clinically significant hypotension | For 48 hours after surgery or until chest tubes were removed, whichever came first |
| Brown  (1989)  USA  Single center, 2 arm parallel group RCT | ‘Elective CABG surgery’ | None reported | 20 (19) | 62.1 | 26.3 | Cardiac | Intravenous, 0.3mcg/kg | 10 minutes, administered immediately after heparin reversal | Placebo (normal saline) | Volume of red cells transfused, total blood loss, reoperation due to bleeding, thromboembolic events, clinically significant hypotension | Up to 48 hours postoperatively |
| Casas  (1995)  Spain  Single center, 3 arm parallel group RCT | ‘Age ≥ 18 years; CABG, valve replacement, annuloplasty, combined valve replacement and CABG, or closure of atrial septal defect’ | ‘Emergency operations; history of a bleeding disorder; allergy or previous exposure to aprotinin’ | 149 (140) | 56.3 | 38.6 | Cardiac | Intravenous, 0.3mcg/kg | 20 to 30 minutes, administered immediately after heparin reversal | Placebo (normal saline), aprotinin | Blood loss up to 24 hours postoperatively (measured by weighing surgical sponges and volume in suction reservoir and drain output; reported as mL/m^2^ body surface area, so not included in meta-analysis), total volume of red cells transfused (not reported in a way that allowed inclusion in this review), number of participants receiving any red cell transfusion, reoperation due to bleeding, thromboembolic events | Up to 24 hours after operation |
| Chuang (1993)  Taiwan  Single center, 2 arm parallel group RCT | ‘Adults undergoing cardiac surgery with cardiopulmonary bypass’ | None reported | 48 (48) | Not reported | Not reported | Cardiac | Intravenous, 0.3mcg/kg | Not reported, administered 1 hour after heparin reversal | Placebo (no details reported) | Blood loss up to 24 hours postoperative (measurement method not reported), volume of red cells transfused | Up to 24 hours post bypass |
| Clagett  (1995)  USA  Single center, 2 arm parallel group RCT | ‘Elective infrarenal aortic aneurysm repair or aortofemoral bypass for occlusive disease’ | ‘Aspiring within 7 days of operation; acquired or congenital haemorrhagic diathesis; emergency operation; creatinine ≥ 3mg/dL; thoracoabdominal reconstruction; aortorenal or visceral bypass’ | 91 (91) | 63.0 | 0.0 | Vascular | Intravenous, 20mcg | 15 minutes, administered immediately after intravenous heparinisation and just before aortic cross-clamp application | Placebo (normal saline) | Total blood loss (reported but unclear if this included time before desmopressin administration, so not included in review; measured by weighing surgical sponges, volume in suction reservoir, and estimates from surgical nurses and anaesthetists), total volume of red cells transfused, number of participants receiving any transfusion, all-cause mortality, thromboembolic events | Up to 72 hours in the postoperative period |
| de Prost (1992)  France  Single center, 2 arm parallel group RCT | ‘Open heart surgery; significant postoperative blood loss (> 75mL/m^2^/h) at any time during the first 6 hours post surgery; prolonged bleeding time (> 10 minutes)’ | ‘<15 years of age; massive mediastinal haemorrhage requiring reoperation’ | 92 (92; 81 for bleeding outcomes) | 57.6 | 25.0 | Cardiac | Intravenous, 0.3mcg/kg | 30 minutes, administered at any time from the end of the operation to 6 hours postoperatively | Placebo (normal saline) | Blood loss after 24 hours (measured by volume of suction drainage; reported in mL/m^2^, so not included in meta-analysis), volume of red cells transfused after 24 hours, reoperation due to bleeding | Patients were monitored for 24 hours after the administration of desmopressin |
| Desborough (2022)  United Kingdom  Multi-centered (3), 2 arm parallel group RCT | ‘Admission to intensive care unit, platelet count of less than 100 × 10^9^/L and were undergoing an invasive procedure, which the treating physician considered to have a bleeding risk’ | ‘Hemorrhagic shock, intervention took place outside research team’s working hours, others not specified’ | 43 (40) | Median age: 58 | 41.9 | Non-surgical procedure | Intravenous, 0.3mcg/kg | 20 minutes, timing of administration not specified | Placebo (normal saline) | Number of participants who received red blood cell transfusion (transfusion protocol not specified) | Up to 24 hours following procedure |
| Despotis (1999)  USA  Single center, 2 arm parallel group RCT | ‘Elective cardiac surgery involving cardiopulmonary bypass; abnormal platelet function after cardiopulmonary bypass (defined as hemoSTATUS <60% in channel 5)’ | ‘Urgent procedures; pre-existing disorders of haemostasis; treatment with antifibrinolytic or antiplatelet agents within 2 days of surgery; intraoperative microvascular bleeding requiring blood component transfusion’ | 101 (101) | 64.8 | 36.6 | Cardiac | Intravenous, 0.4mcg/kg | 30 minutes, timing of administration unclear | Placebo (normal saline) | Total blood loss (measured by drain output), volume of red cells transfused, reoperation due to bleeding, all-cause mortality, thromboembolic events, clinically important hypotension | In the first 24 hours after surgery |
| Dilthey  (1993)  Germany  Single center, 2 arm parallel group RCT | ‘Elective first-time myocardial revascularisation; aspirin within previous 5 days; male’ | ‘Preoperative haemoglobin < 135 g/L; preoperative prolongation of PT or aPTT; any anticoagulant treatment other than aspirin; intraoperative use of aprotinin’ | 40 (39) | 57.1 | 0.0 | Cardiac | Intravenous, 0.3mcg/kg | 15 minutes, administered 5 minutes after heparin reversal | Placebo (normal saline) | Total volume of red cells transfused (reported as median and range, so not included in meta-analysis), number of participants receiving a red cell transfusion, total blood loss (measured by drain output; reported as median and range, so not included in meta-analysis), clinically significant hypotension | Up to 7 days postoperatively |
| Ellis  (2001)  Israel  Single center, open label, 3 arm parallel group RCT | ‘ASA scale 1-3; undergoing elective total knee replacement’ | ‘New York Heart Association 3 or 4 classified heart failure; chronic renal failure; liver cirrhosis; bleeding disorders; current anticoagulant therapy’ | 30 (30) | 71.7 | 70.0 | Orthopedic | Intravenous, 0.3mcg/kg | 30 minutes, administered 30 minutes before tourniquet removed | Standard care, tranexamic acid | Total volume of red cells transfused (reported as mean only, so not included in meta-analysis), number of participants receiving a red cell transfusion | Up to 35 days after surgery |
| Flordal  (1991)  Sweden  Single center, 2 arm parallel group RCT | ‘Undergoing total hip replacement’ | ‘Prostaglandin synthesis inhibitors’ | 12 (12) | Not reported | Not reported | Orthopedic | Intravenous, 0.3mcg/kg | 20 to 30 minutes, administered at the start of surgery and again 6 hours postoperatively | Placebo (normal saline) | Total blood loss (measurement method not reported; reported as mean only, so not included in meta-analysis), thromboembolic events | Up to 24 hours after surgery |
| Flordal  (1992)  Sweden  Single center, 2 arm parallel group RCT | ‘Elective total hip replacement’ | ‘> 80 years old; severe vascular, hepatic, or renal disease; prostaglandin synthesis inhibitors’ | 50 (50) | 66.0 | 52.0 | Orthopedic | Intravenous, 0.3mcg/kg | 20 to 30 minutes, administered at the start of surgery and again 6 hours postoperatively | Placebo (normal saline) | Intraoperative and total blood loss (measured by estimating blood in surgical swabs, paper drapes, and folds; volume in suction reservoir; and change in hemoglobin preoperatively and postoperatively compared with estimated total blood volume), volume of red cells transfused, thromboembolic events | Up to 24 hours postoperatively |
| Frankville (1991)  USA  Single center, 2 arm parallel group RCT | ‘Elective primary CABG’ | ‘Warfarin or heparin within 24 hours of surgery; documented coagulopathies or platelet disorders; allergy to desmopressin; renal failure; stroke or venous thromboembolism within 3 months’ | 40 (40) | 59.8 | 15.0 | Cardiac | Intravenous, 0.3mcg/kg | 15 minutes, administered 5 minutes after heparin reversal | Placebo (normal saline) | Clinically significant hypotension, total blood loss (measured by drain output), number of participants receiving a red cell transfusion, volume of red cells transfused, reoperation due to bleeding | Up to 24 hours postoperatively, or until the tubes were removed |
| Gratz  (1992)  USA  Single center, 2 arm parallel group RCT | ‘Elective CABG operations; aspiring within 7 days of surgery’ | ‘Valvular heart disease; need for intra-aortic balloon pump; re-doing CABG’ | 65 (59) | 62.3 | 28.8 | Cardiac | Intravenous, 0.3mcg/kg | 30 minutes, administered immediately after heparin reversal | Placebo (normal saline) | Total blood loss (measured by weighing surgical sponges, volume in cell saver, and suction drainage), volume of red cells transfused, number of participants receiving a red cell transfusion, all-cause mortality, thromboembolic events | For the first 24 hours postoperatively |
| Guay  (1992)  Canada  Single center, 2 arm parallel group RCT | ‘ASA 1-2; idiopathic scoliosis; undergoing scheduled spinal fusion surgery’ | ‘Different surgical technique used; history of bleeding diathesis; ingestion of drugs known to interfere with haemostasis; abnormal bleeding time (> 9 minutes); aPTT > 36 seconds; PT > 25 seconds; TT > 16 seconds; platelet count < 150 x 10^9^/L’ | 31 (30) | 14.3 | 93.3 | Orthopedic | Intravenous, 10mcg/m^2^ body surface area | 20 minutes, administered at time of first skin incision | Placebo (normal saline) | Total blood loss (measurement method not reported), volume of red cells transfused (no report of total volume transfused), reoperation due to bleeding | The postoperative period up to 24 hours |
| Guyuron (1996)  USA  Single center, 2 arm parallel group RCT | ‘Bimaxillary osteotomy; normal preoperative PT and aPTT; no history of bleeding disorder or easy bruising’ | None reported | 20 (20) | Not reported | 75.0 | Maxillofacial | Intravenous, 20mcg | 30 minutes, administered 30 minutes preoperatively | Placebo (normal saline) | Blood loss up to 24 hours postoperative (measured by estimating blood loss in surgical sponges and suction drainage), total volume of red cells transfused, number of participants receiving a red cell transfusion | Up to 24 hours postoperatively |
| Hackmann (1989)  Canada  Single center, 2 arm parallel group RCT | ‘> 18 years old; elective cardiac surgery involving cardiopulmonary bypass’ | ‘Pregnancy; known bleeding disorder such as haemophilia, von Willebrand disease, or immune thrombocytopenic purpura; abnormal coagulation (PT, aPTT or TT); platelet count < 100 x 10^9^/L; clotting parameters that had not returned to normal after cessation of anticoagulant drugs’ | 164 (150) | Not reported | Not reported | Cardiac | Intravenous, 0.3mcg/kg | 15 minutes, administered immediately after heparin reversal | Placebo (normal saline) | Perioperative and total blood loss (measured by weighing surgical sponges, estimating blood on surgical drapes, measuring suction bottles and drain output), volume of red cells transfused, number of participants receiving a red cell transfusion, reoperation due to bleeding, all-cause mortality | Up to 24 hours after the completion of study-drug infusion |
| Hajimohamadi  (2021)  Iran  Single center, 2 arm parallel group RCT | ‘Chronic rhinosinusitis with or without polyposis between 15 and 60 years' old who were entrants for FESS surgery’ | ‘Underlying bleeding disorders, history of cardiovascular disease, hypertension and diabetics; Patients who needed revision FESS surgery. Patients under 15 or over 60 years’ | 44 (44) | 38.7 | 29.5 | Ear, nose and throat (functional endoscopic sinus surgery) | Intravenous, 0.2mcg/kg | Not reported, administered 1 minute before surgery +/- 30 minutes after surgery | Placebo (normal saline) | Total volume of blood loss (volume of blood suctioned minus serum used for irrigation, number of bloody gauzes) | Up to 12 hours following surgery |
| Hajjar  (2017)  Brazil  2 arm parallel group RCT (unclear is single or multicenter) | ‘Cardiac surgery requiring cardiopulmonary bypass’ | Not reported | Total number of participants randomized not reported (150) | Not reported | Not reported | Cardiac | Intravenous, 0.3mcg/kg | 15 minutes, administered immediately after the end of surgery | Placebo (normal saline) | Blood loss up to 72 hours (method not reported; reported as mL/m^2,^ so not included in meta-analysis), volume of red cells transfused (reported in mL and converted to units by assuming 1 unit to be equivalent to 300 mL), thromboembolic events (prespecified but not reported) | In the first 72 hours after surgery |
| Hedderich (1990)  Canada  2 arm parallel group RCT (unclear is single or multicenter) | ‘Uncomplicated CABG’ | Not reported | 62 (59 to 62) | 60.0 | 24.2 | Cardiac | Intravenous, 0.3mcg/kg | 15 minutes, administered immediately after heparin reversal | Placebo (normal saline) | Total blood loss (measured by weighing surgical sponges, suction drainage, and drain output), volume of red cells transfused total, reoperation due to bleeding, all-cause mortality, thromboembolic events | Up to 24 hours postoperatively |
| Horrow (1991a)  USA  Single center, 4 arm parallel group RCT | ‘Elective cardiac surgery’ | ‘Warfarin or oestrogens within 7 days of surgery; active haematuria; serum creatinine ≥ 2 mg/dL; personal or family history of abnormal bleeding; intra-aortic balloon counterpulsation’ | 84 (82) | 63.5 | Not reported | Cardiac | Intravenous, 0.3mcg/kg | 20 minutes, administered after heparin reversal | Placebo (normal saline) | Blood loss (measured by drain output), number of participants receiving a red cell transfusion, reoperation due to bleeding, thromboembolic events | Up to 5 days of operation |
| Horrow (1991b)  USA  Single center, 4 arm parallel group RCT | ‘Elective cardiac surgery’ | ‘Warfarin or oestrogens within 7 days of surgery; active haematuria; serum creatinine ≥ 2 mg/dL; personal or family history of abnormal bleeding; intra-aortic balloon counterpulsation’ | 79 (77) | 63.9 | Not reported | Cardiac | Intravenous, 0.3mcg/kg | 20 minutes, administered after heparin reversal. Tranexamic acid 10mg/kg loading dose after induction of anesthesia and before first skin incision over 30 minutes, then 1 mg/kg/h for 10 hours | Tranexamic acid and placebo (normal saline) | Blood loss (measured by drain output), number of participants receiving a red cell transfusion, reoperation due to bleeding, thromboembolic events | Up to 5 days of operation |
| Horrow (1991c)  USA  Single center, 4 arm parallel group RCT | ‘Elective cardiac surgery’ | ‘Warfarin or oestrogens within 7 days of surgery; active haematuria; serum creatinine ≥ 2 mg/dL; personal or family history of abnormal bleeding; intra-aortic balloon counterpulsation’ | 77 (75) | 64.0 | Not reported | Cardiac | Intravenous, 0.3mcg/kg | 20 minutes, administered after heparin reversal | Tranexamic acid) | Blood loss (measured by drain output), number of participants receiving a red cell transfusion, reoperation due to bleeding, thromboembolic events | Up to 5 days of operation |
| Jahangirifard (2017)  Iran  Single center, 2 arm parallel group RCT | ‘Scheduled to undergo heart transplantation’ | ‘20< age >70 years, history of previous surgery on the chest, bleeding haemophilia problems, patients with pre-operative platelet coagulation disorders, any problem where desmopressin is contraindicated, history of deep vein thrombosis, haematologic disease, carotid plaque that leads to obvious and chronic obstructive pulmonary disease’ | 48 (48) | 47 | 68.8 | Cardiac | Intravenous, 0.3mcg/kg | 10 minutes, administered 30 minutes before surgery | Placebo (normal saline) | Total volume of blood loss (chest tube drainage), volume of red blood cell transfused (transfusion protocol not specified) | Up to 48hours following surgery |
| Jin  (2015)  China  Single center, 2 arm parallel group RCT | ‘Undergoing elective valvular surgery; ASA classification 2-3; no coronary heart disease or decompensated heart failure; no blood disease (no further information given); normal preoperative coagulation tests and platelet count; no anticoagulant or hemostasis treatment for 1 week before surgery’ | ‘Emergency or repeat surgery’ | 102 (102) | 51.0 | 58.8 | Cardiac | Intravenous, 0.3mcg/kg | 10 minutes, administered 30 minutes before cardiac re-warming | Placebo (normal saline) | Total blood loss (method for measurement not reported), number of participants receiving a red cell transfusion, all-cause mortality, thromboembolic events | Up to 24 hours after surgery |
| Karnezis (1994a)  USA  Two separate single centers, 2 arm parallel group RCTs | ‘Primary total knee replacement’ | ‘History of operative intervention involving hip or knee; coagulation disorder; coronary artery disease; warfarin or hearing within 7 days of procedure; bilateral or revision procedures’ | 36 (36) | 65.5 | 55.6 | Orthopedic | Intravenous, 0.3mcg/kg | 20 minutes, administered 30 minutes before complete closure of the wound | Placebo (normal saline) | Blood loss (reported graphically and not possible to extract these data accurately for meta-analysis; measured by drain output), volume of red cells transfused, all-cause mortality, thromboembolic events | Up to 48 hours postoperatively |
| Karnezis (1994b)  USA  Two separate single centers, 2 arm parallel group RCTs | ‘Primary total hip replacement’ | ‘History of operative intervention involving hip or knee; coagulation disorder; coronary artery disease; warfarin or hearing within 7 days of procedure; bilateral or revision procedures’ | 56 (56) | 66.1 | 53.6 | Orthopedic | Intravenous, 0.3mcg/kg | 20 minutes, administered 30 minutes before complete closure of the wound | Placebo (normal saline) | Blood loss (reported graphically and not possible to extract these data accurately for meta-analysis; measured by drain output), volume of red cells transfused, all-cause mortality, thromboembolic events | Up to 48 hours postoperatively |
| Kobrinsky (1987)  Canada  Single center, 2 arm parallel group RCT | ‘Scheduled spinal fusion with Harrington rod instrumentation’ | ‘Bleeding diathesis; aspirin within 14 days; bleeding time > 9 minutes or preoperative screen’ | 35 (35) | 15.1 | 57.1 | Orthopedic | Intravenous, 10mcg/m^2^ body surface area | 20 minutes, administered immediately after induction of anaesthesia | Placebo (no details reported) | Total blood loss (measured by weighing surgical sponges and suction drainage), volume of red cells transfused intraoperatively | Up to 14 days after surgery |
| Kuitunen (1992)  Finland  Single center, 2 arm parallel group RCT | ‘Elective primary CABG’ | ‘Previous cardiac surgery; coagulation disorder; coumarin anticoagulant, heparin, or acetylsalicylic acid within 5 days of surgery’ | 33 (30) | 58.0 | 6.7 | Cardiac | Intravenous, 0.3mcg/kg | 15 minutes, administered immediately after sternal closure | Placebo (normal saline) | Total volume of red cells transfused (reported as mean and range, so not included in meta-analysis), total blood loss (measured by drain output), all-cause mortality | Up to 16 hours after treatment |
| Lee  (2010) | ‘Uremic patients not on dialysis; undergoing dialysis catheter insertion; prolonged closure time on platelet function analyser’ | ‘Chronic liver disease; infectious diseases (not specified); drugs that interfere with platelet function within 10 days of entering the study’ | 48 (48) | Median desmopressin arm: 60; placebo arm: 57 | 35.4 | Dialysis catheter insertion | Intravenous, 0.3mcg/kg | 30 minutes, timing of infusion unknown | Placebo (normal saline) | Reoperation due to bleeding, number of participants with any bleeding | Unknown |
| Leino  (2010)  Finland  Single centre, 3 arm parallel group RCT | ‘Seropositive rheumatoid arthritis; scheduled for total hip arthroplasty under spinal  Anaesthesia’ | ‘Revision arthroplasty; contraindications for spinal anaesthesia; hepatic malfunction  assessed by "thorough anamnesis"; renal malfunction assessed by serum creatinine; "anamnestic" or  diagnosed coagulation disorder; warfarin treatment; any treatment other than acetylsalicylic acid or  NSAIDs affecting thrombocyte function or other components of coagulation; later excluded 4 participants who experienced intraoperative surgical problems with ensuing blood loss over 400 mL’ | 75 (71) | 61 | 40.8 | Orthopedic | Intravenous, 0.2 to 0.4µg/kg | 30 minutes, at start of surgery | Placebo (normal saline) | Total blood loss (measured by estimating blood loss from surgical swabs and suction drainage), volume of red cells transfused intraoperatively and total volume of red cells transfused (red cells transfused if haemoglobin < 90 g/L), intraoperative blood loss, thromboembolic events | Up to 96 hours from the arrival to the surgical ward |
| Lethagen  (1991)  Sweden  Single centre, 2 arm parallel group RCT | ‘Elective surgery aortoiliac graft surgery for aortoiliac occlusive disease or  Aneurysms’ | ‘History of increased bleeding tendency; prolonged preoperative bleeding time;  acetylsalicylic acid within 10 days before surgery’ | 50 (50) | Not reported | 26.0 | Cardiac | Intravenous, 0.3µg/kg | 10 minutes, immediately before the start of the operation | Placebo (normal saline) | Blood loss (measured by estimating blood loss in surgical swabs, suction bottles, and drain output), volume of red cells transfused (transfused if haematocrit < 30%), thromboembolic events | Up to 48 hours following surgery |
| Letts  (1998)  Canada  2 arm parallel group RCT (unclear whether single centre or multi-centre trial) | ‘Paediatric patients undergoing spine fusion for neuromuscular scoliosis’ | Not reported | 30 (30) | 13.6 | 40.0 | Orthopedic | Route of administration not reported, 10μg/m2 body surface area | Duration not reported, immediately after induction of anaesthesia | Placebo (normal saline) | Blood loss (measured by estimating blood loss in surgical sponges  and suction drainage), volume of red cells transfused (transfusion protocol not reported), clinically significant hypotension | Up to 1 hour following administration of intervention |
| Manno  (2011)  Italy  Single centre, 2 arm parallel group RCT | ‘Undergoing percutaneous ultrasound-guided biopsy of the native kidney in the Bari  renal unit; aged 16 to 80 years; blood pressure 140/90 mmHg with or without antihypertensive therapy;  serum creatinine level < 1.5 mg/dL and/or estimated glomerular filtration rate (GFR) < 60 mL/min/1.73  m2 (calculated by the Modification of Diet in Renal Disease (MDRD) study equation); normal coagulation parameters (bleeding time evaluated by the Simplate method, with values for prothrombin time,  partial thromboplastin time, platelets, and fibrinogen in the reference range)’ | ‘Solitary kidney; kidney cancer; hydro-/pyonephrosis; significantly decreased kidney  size on ultrasound image; severe obesity (body mass index 30 kg/m2); acute kidney injury’ | 162 (162) | 40.6 | 45.7 | Renal biopsy | Subcutaneous, 0.3µg/kg | 1 hour before the biopsy | Placebo (normal saline) | Number of participants with any bleeding (number of participants with a  haematoma ≥ 20 mm diameter or haematuria), Number of participants requiring a red cell transfusion intraoperatively and in total (transfusion protocol not reported), Thromboembolic events, Clinically important hypotension | Up to 72 hours following biopsy |
| Marczinski  (2007)  Netherlands  Single centre, 2 arm parallel group RCT | ‘> 18 years old; taking a serotonergic antidepressant (fluvoxamine, fluoxetine, paroxetine, sertraline, venlafaxine, lomipramine, citalopram) for at least 2 weeks; undergoing orthopaedic,  abdominal, or breast surgery’ | ‘No informed consent; primary haemostasis disorder; hyponatraemia (sodium  (serum) < 130 mmol/L); laparoscopic surgery; use of vitamin K antagonists, aspirin, iron supplements,  methotrexate, or heparin; acute coronary syndrome (unstable angina or myocardial infarction); spinal  anaesthesia during surgery’ | 28 (28) | 51.6 | 92.6 | Abdominal, breast, or orthopedic surgery | Route of administration not reported, 15μg if body weight < 50 kg; 30μg if body weight 50 kg to 100 kg; and 45μg  if body weight > 100 kg | Not reported | Placebo (normal saline) | Blood loss (measured by estimating blood loss in surgical gauze and  drain output), number of participants receiving a red cell transfusion intraoperatively (transfusion protocol not reported) | Up to 48 hours after the start of surgery |
| Marquez  (1992)  USA  Single centre, 3 arm parallel group RCT | ‘CABG without prior cardiac surgery; no aspirin, NSAIDs, coumarin, or heparin administration (10 days before surgery)’ | ‘Mediastinal exploration for surgical bleeding or haemodynamic instability pre-CPB  or post-CPB, but before DDAVP administration’ | 70 (65) | 61.7 | Not reported | Cardiac | Intravenous, 0.3µg/kg | Duration not reported, immediately after heparin reversal and again 12 hours postoperatively for the first intervention group | Placebo (normal saline) | Total blood loss (measured by estimating blood loss in surgical sponges and suction  drainage), total volume of red cells transfused, number of participants receiving a red cell transfusion (red cells transfused if haemoglobin < 100 g/L), thromboembolic events, clinically important hypotension | Up to 36 hours following surgery |
| Mongan  (1992a)  USA  Single centre, 2 arm parallel group RCT  Reported outcomes of participants with thromboelastography maximum amplitude >50mm | ‘Elective primary CABG’ | ‘Preoperative anticoagulation/aspirin within 1 week of surgery; re-exploration due  to surgical bleeding; postoperative evidence of fibrinolysis’ | 86 (86) | 61.2 | 11.6 | Cardiac | Intravenous, 0.3µg/kg | 15 minutes, after heparin reversal and before chest closure | Placebo (normal saline) | Blood loss (measured by drain output), volume of red cell transfusion, number of participants receiving a red cell transfusion (transfused if haematocrit < 24%), all-cause mortality, thromboembolic events, clinically important hypotension | Up to 24 hours following admission to the ICU |
| Mongan  (1992b)  USA  Single centre, 2 arm parallel group RCT  Reported outcomes of participants with thromboelastography maximum amplitude <50mm | ‘Elective primary CABG’ | ‘Preoperative anticoagulation/aspirin within 1 week of surgery; re-exploration due  to surgical bleeding; postoperative evidence of fibrinolysis’ | 3 participants were excluded between Mongan 1992a and 1992b before administration of DDAVP or placebo (29) | 62.7 | 31.0 | Cardiac | Intravenous, 0.3µg/kg | 15 minutes, after heparin reversal and before chest closure | Placebo (normal saline) | Blood loss (measured by drain output), volume of red cell transfusion, number of participants receiving a red cell transfusion (transfused if haematocrit < 24%), all-cause mortality, thromboembolic events, clinically important hypotension | Up to 24 hours following admission to the ICU |
| Oliver  (2000)  USA  Single centre, 2 arm parallel group RCT | ‘< 40 years old; undergoing complex congenital heart operation requiring cardiopulmonary bypass’ | ‘Operation expected to have minimal blood loss; pre-existing bleeding disorder’ | 60 (60) | 15.9 | 56.7 | Paediatric cardiac | Intravenous, 0.3µg/kg | 20 to 30 minutes, 10  minutes after heparin reversal and after aPTT returned to within 10% of normal | Placebo (normal saline) | Blood loss  (measured by volume of suction drainage), volume of red cell transfusion, number of participants receiving a red cell transfusion (transfusion protocol not reported), reoperation due to bleeding, all-cause mortality, clinically significant hypotension | Up to 43 days following admission to ICU |
| Ozkisacik  (2001)  Turkey  Single centre, 2 arm parallel group RCT | ‘Undergoing elective CABG’ | ‘Emergency surgery; haemostatic defect; hypertension; diabetes; renal failure’ | 66 (66) | 58.6 | 28.8 | Cardiac | Intravenous, 0.3µg/kg | 20 minutes, after heparin reversal | Placebo (normal saline) | Postoperative blood loss (measured by drain output), volume of red cells transfused, number of participants receiving a red cell transfusion (transfused if haematocrit < 28%), reoperation due to bleeding | Up to 24 hours after the surgery |
| Pleym  (2004)  Norway  2 arm parallel group RCT (unclear whether single centre or multi-centre trial) | ‘Stable angina pectoris; elective first-time CABG; taking aspirin’ | ‘Treatment with heparin or low molecular weight heparin, oral anticoagulants,  NSAIDs or other platelet inhibitors’ | 100 (92) | 63.8 | 17.4 | Cardiac | Intravenous, 0.3µg/kg | 10 minutes, immediately after heparin reversal | Placebo (normal saline) | Blood loss (measured by drain output), number of participants receiving a red cell transfusion (transfusion if haematocrit < 25%), reoperation due to bleeding, all-cause mortality, thromboembolic events, clinically important hypotension | Mean of 6.7 days after surgery |
| Reich  (1991)  USA  Single centre, 2 arm parallel group RCT | ‘Adults scheduled to undergo elective myocardial revascularisation or single-valve  replacement surgery; left ventricular ejection fraction > 0.40; normal preoperative coagulation profile  (prothrombin time, partial thromboplastin time, platelet count, fibrinogen, template bleeding time)’ | ‘Haemodynamic instability; preoperative heparin therapy within 48 hours; refusal of  blood products (Jehovah’s Witnesses)’ | 27 (27) | 58 | 22.2 | Cardiac | Intravenous, 0.3µg/kg | 10 minutes, 15 minutes  after heparin reversal | Placebo (normal saline) | Clinically significant hypotension, blood loss (measured by drain output), volume of red cells transfused (transfusion protocol not reported) | Up to 24 hours after infusion |
| Reynolds  (1993)  USA  Single centre, 2 arm parallel group RCT | ‘Paediatric patients ranging in age from 1 day to 16 years of age; scheduled for cardiac operations’ | Not reported | 112 (95) | 26 months | 43.2 | Paediatric cardiac | Intravenous, 0.3µg/kg | 15 minutes, 5 minutes after heparin reversal | Placebo (normal saline) | Blood loss (measured by estimating blood loss in  surgical sponges, volume of suction drainage and drain output), volume of red cells transfused (transfusion protocol not reported) | Up to 24 hours after infusion |
| Rocha  (1988)  Spain  Single centre, 2 arm parallel group RCT | ‘> 18 years old; valvular heart disease or atrial septal defect’ | ‘Emergency surgery; known haemostatic defect; uncontrolled hypertension; renal  insufficiency; patients undergoing CABG’ | 100 (100) | 54 | 56.0 | Cardiac | Intravenous, 0.3µg/kg | 15 minutes, immediately after heparin reversal | Placebo (normal saline) | Blood loss (measured by estimating blood loss in surgical sponges and drain output), volume of red cells transfused (transfusion protocol not reported), reoperation due to bleeding, all-cause mortality, thromboembolic events up to 3 days postoperatively | Up to 72 hours after surgery |
| Salmenpera  (1991)  Finland  Single centre, 2 arm parallel group RCT | ‘First-time CABG procedure; bleeding history unremarkable; normal preoperative  blood coagulation tests’ | ‘People who had received acetylsalicylic acid or heparin within 5 days’ | 30 (30) | 58 | 6.7 | Cardiac | Side-port of pulmonary artery catheter introducer, 0.3µg/kg | 15 minutes, immediately after sternal closure | Placebo (normal saline) | Clinically significant hypotension, blood loss (method for measurement not reported), thromboembolic events | Not reported |
| Salzman  (1986)  USA  Single centre, 2 arm parallel group RCT | ‘Undergoing CABG with valvular heart disease, atrial septal defects, or undergoing  repeat grafting operations for chronically occluded CABGs’ | ‘Undergoing primary uncomplicated CABG’ | 72 (70) | 31-40 years: 5  41-50 years: 4  51-60 years: 18  61-70 years: 19  71-80 years: 20  > 80 years: 4 | 40.0 | Cardiac | Intravenous, 0.3µg/kg | 15 minutes, immediately after heparin reversal | Placebo (normal saline) | Blood loss (measured by estimating blood in surgical sponges and suction drainage), total volume of red cells transfused (transfusion protocol not reported), reoperation due to bleeding, all-cause mortality, thromboembolic events, clinically significant hypotension | Up to 24 hours after surgery |
| Schott  (1995)  Sweden  Single centre, 2 arm parallel group RCT | ‘Normal haemostasis; scheduled for elective primary total hip replacement’ | ‘Secondary procedure; antiplatelet drug within 10 days of surgery; iron-deficient  anaemia; diabetes mellitus; rheumatoid disease; any disease requiring steroid treatment; abnormal  preoperative coagulation status; abnormal bleeding time’ | 80 (79) | 69 | 55.7 | Orthopedic | Intravenous, 0.3µg/kg | 15 minutes, post induction of spinal  anaesthesia and again 6 hours after first dose | Placebo (normal saline) | Blood loss (measured by estimating blood loss in  surgical swabs and drapes; volume in suction bottle; and drain output), volume of red cells transfused perioperatively (transfused if haematocrit < 27%), all-cause mortality, thromboembolic events, clinically important hypotension | Up to 24 hours after first infusion |
| Seear  (1989)  Canada  Single centre, 2 arm parallel group RCT | ‘Paediatric patients undergoing surgery with cardiac bypass’ | Not reported | 60 (60) | 55.0 months | 41.7 | Paediatric cardiac | Intravenous, 0.3µg/kg | 15 minutes, on conclusion of cardiopulmonary bypass | Placebo (normal saline) | Blood loss (measured by estimating blood loss in surgical sponges  and drain output), all-cause mortality | Up to 24 hours after infusion |
| Shao  (2015)  China  Single centre, 2 arm parallel group RCT | ‘Age 18 to 65 years; first-time candidates for 2-side endoscopic sinus surgery; ASA  grade 1-2’ | ‘History of bleeding disorders; medications that may affect surgical haemostasis; secondary surgery; poorly controlled hypertension; cerebrovascular disease; significant coronary  artery disease or arrhythmias; compromised renal or hepatic function; pregnancy’ | 90 (90) | 43.3 | 44.4 | Endoscopic sinus | Intravenous, 0.3µg/kg | 20 minutes, post anaesthetic induction  and preoperatively | Placebo (normal saline) | Blood loss (quality of operative field determined by operating surgeon), thromboembolic events, clinically significant hypotension | Not reported |
| Sheridan  (1994)  Canada  Single centre, 2 arm parallel group RCT | ‘Male; < 70 years old; taking aspirin within previous 7 days; undergoing CABG’ | ‘Abnormal haematological profile; history of bleeding; repeat coronary bypass surgery; recent heparin intake’ | 44 (44) | 59.3 | 0 | Cardiac | Intravenous, 10μg/m2 body surface area | 20 minutes, after cardiopulmonary bypass | Placebo (normal saline) | Blood loss (measured by volume of suction drainage and estimated  cardiopulmonary bypass residual volume), number of participants receiving a red cell transfusion (transfusion protocol not reported), all-cause mortality, thromboembolic events | Up to 24 hours after surgery |
| Spyt  (1990)  UK  Single centre, 2 arm parallel group RCT | ‘Elective CABG with cardiopulmonary bypass grafting; men age 30 to 70 years and  women who were postmenopausal and < 70 years old’ | ‘Reoperation or emergency surgery; heparin or warfarin within 72 hours of surgery;  thrombolytic therapy within 7 days before surgery; no informed consent’ | 100 (98) | 56.9 | 18.4 | Cardiac | Intravenous, 0.3µg/kg | 30 minutes, after heparin reversal | Placebo (normal saline) | Blood loss (measured by estimating blood loss from surgical swabs,  suction drainage, and drain output), volume of red cells transfused (red cells transfused if haematocrit < 30%), number of participants receiving a red cell transfusion | Up to 48 hours |
| Steinlechner  (2011)  Austria  Single centre, 2 arm parallel group RCT | ‘Elective bioprosthetic AVR because of severe aortic valve stenosis (defined as a  mean gradient of 50 mmHg or an indexed effective orifice area of 0.5 cm2/m2 body surface area);  platelet dysfunction (collagen/adenosine diphosphate closure time on platelet function analyser-100 >  170 seconds)’ | ‘Left ventricular ejection fraction < 0.40; body mass index > 40 kg/m2; serum creatinine > 1.5 mg/dL; known hypersensitivity towards DDAVP; active endocarditis; multi-valvular disease;  antiplatelet therapy within 10 days before surgery; any relevant coronary artery disease; inability to  give informed consent; mechanical valves’ | 50 (43) | 72 | 65.1 | Cardiac | Intravenous, 0.3µg/kg | 30 minutes, after induction of anaesthesia | Placebo (normal saline) | Blood loss (method for measurement not reported), volume of red cells transfused (red cells transfused when haemoglobin <70g/L in the operating room, or <80g/L on the ward), reoperation for bleeding, all-cause mortality, thromboembolic events | Up to 24 hours after surgery |
| Temeck  (1994)  USA  Single centre, 2 arm parallel group RCT | ‘Undergoing primary cardiac surgery’ | Not reported | 83 (83) | Not reported | Not reported | Cardiac | Intravenous, 0.3µg/kg | 15 minutes, after heparin | Placebo (normal saline) | Blood loss (measured by drain output), number of participants receiving a red cell transfusion (transfusion protocol not reported) | Up to 24 hours after surgery |
| Vafaee  (2020)  Iran  Single center, 2 arm parallel group RCT | ‘Degenerative lumbosacral disease; informed consent to enter the research project’ | ‘Acute heart disease; polydipsia; severe heart failure; hyponatremia; moderate and severe renal failure; hypertension; coagulation problems’ | 55 (55) | 50.6 | 67.3 | Orthopedics | Intravenous, 0.3mcg/kg | 10 minutes, prior to surgery | Placebo (normal saline) | Total volume of blood loss (intraoperative blood loss and Hemobag), volume of red blood cells transfused (per specified calculation) | Up to 24hour after transfer to the ward or after surgery |
| Wang  (2020)  China  Multi-center (3), 2 arm parallel group RCT | ‘Patients who agreed to participate in the study and signed the informed consent form; 2) aged 18-75 years; and 3) patients with 400-1000 mL of intraoperative blood loss who underwent gastrointestinal surgery’ | ‘Secondary surgery; pregnant and lactating patients; platelet count <5×109/L; hematologic disease; international normalized ratio >2.0; unstable angina pectoris, and myocardial infarction within six months and had a history of congenital heart disease, pulmonary heart disease, and cardiac insufficiency; hypertension (blood pressure >180/110 mmHg); hemorrhagic stroke; type 2B von Willebrand disease; serum bilirubin, alanine aminotransferase, aspartate aminotransferase, urea nitrogen, and serum creatinine that were 1.5 times higher than normal; fasting blood glucose >15 mmol/L; serum sodium <120 g/L; serum albumin <25 g/L; hyperadrenocorticism, hyperthyroidism, and hypothyroidism; idiopathic edema; bladder outlet obstruction, or urine flow <5 mL/s; central or renal diabetes insipidus; history of blood transfusion within one month; bladder cancer or prostate cancer; allergic to DDAVP; treated with DEMOPRESSIN within three months; history of drug and alcohol dependence or abuse; participated in other clinical trials within three months; refused to participate or did not sign the informed consent form’ | 59 (48) | 64.2 | 37.5 | Gastrointestinal | Intravenous, 0.3mcg/kg | 30 minutes, administered once per day after surgery | Placebo (normal saline) | Total volume of blood loss (abdominal drainage), number of participants who received red blood cell transfusion (transfusion protocol not specified) | Up to 24hours following surgery |
| Wingate  (1992a)  USA  Single centre, 2 arm parallel group RCT | ‘Spinal cord injury requiring flap reconstruction of pelvic pressure sores’ | Not reported | 23 (23) | Not reported | Not reported | Plastic | Intravenous, 0.3µg/kg | 20 minutes, after induction before operation | Placebo (normal saline) | Blood loss (method for measurement not reported), volume of red cells transfused (transfusion protocol not reported), number of participants receiving a red cell transfusion | Up to 24 hours after surgery |
| Wingate  (1992b)  USA  Single centre, 2 arm parallel group RCT | ‘Spinal cord injury requiring flap reconstruction of pelvic pressure sores’ | Not reported | 21 (21) | Not reported | Not reported | Plastic | Intravenous, 0.3µg/kg | 20 minutes, after induction before operation | Placebo (normal saline) | Blood loss (method for measurement not reported), volume of red cells transfused (transfusion protocol not reported), number of participants receiving a red cell transfusion | Up to 24 hours after surgery |
| Wong  (2003)  Hong Kong  Single centre, 2 arm parallel group RCT | ‘Adults scheduled for hepatectomy’ | ‘Coronary artery disease; congenital or acquired coagulation disorders other than  liver cirrhosis; blood sodium level < 130 mmol/L; NSAID or aspirin ingestion within 7 days of scheduled  surgery; history of thrombovascular disorders or pulmonary thromboembolism’ | 60 (59) | 51.2 | 38.3 | Hepatic | Intravenous, 0.3µg/kg | 20 minutes, after induction of anaesthesia | Placebo (normal saline) | Blood loss (measured by estimating blood loss in surgical swabs and drain output), total volume of red cells transfused, number of participants receiving a red cell transfusion (red cell transfusion if haematocrit <30%) | Up to 1 hour after infusion |
| Youssefy (2022)  Iran  Single center, 2 arm parallel group RCT | ‘Candidates for primary rhinoplasty surgery based on cosmetic appearance (not revision surgery)’ | ‘Underlying renal disease, hepatic disease, heart disease, neurologic problems of any kind, bleeding problems, coagulopathies, certain medications (OCP, warfarin, meds that alter hemostasis), patients requiring rib cartilage for rhinoseptoplasty’ | 70 (70) | 30.2 | 52.9 | Plastic | Intravenous, 0.1 to 0.3mcg/kg | Not reported, administered 30 minutes prior to surgery | Placebo (normal saline) | Total volume of blood loss (volume of blood suctioned minus serum used for irrigation, number of bloody gauzes) | End of or immediately after surgery |
| Zohar  (2001)  Israel  Single centre, 2 arm parallel group RCT | ‘ASA physical status 1-3; undergoing elective total knee replacement’ | ‘Severe ischaemic heart disease (New York Heart Association grade III or IV); chronic  renal failure; liver cirrhosis; bleeding disorders; anticoagulant therapy’ | 40 (40) | 72 | 72.5 | Orthopedic | Intravenous, 0.3µg/kg | Duration of desmopressin is not reported, 30 minutes before deflation  of tourniquet. Followed by a constant infusion of intravenous  saline until 12 hours after surgery | Tranexamic acid (15mg/kg), 30 minutes before deflation of tourniquet (speed of administration not reported). Followed by a constant infusion of tranexamic acid 10mg/kg until 12 hours  after surgery | Total volume of red cells transfused, blood loss total (measured by drain output), number of participants receiving a red cell transfusion (red cells transfused if haematocrit <27%), thromboembolism | Up to 3 months after hospital discharge |

**Footnote**: Abbreviations: RCT, randomized controlled trial; CABG, coronary artery bypass graft; aPTT, activated partial thromboplastin time; PT, prothrombin time; TT, thrombin time.

*Information pertaining to the studies published prior to 2017 were adopted from the previous Cochrane Review.^1^

Reference:

1. Desborough MJ, Oakland K, Brierley C et al. Desmopressin use for minimising perioperative blood transfusion. *Cochrane Database of Systematic Reviews* 2017; **2017**.

## Table S3. Individual studies that reported the baseline kidney function of participants.

| Author (Year) | Reported baseline (preoperative) kidney function (creatinine in mg/dL) of participants |
| --- | --- |
| Altun (2017) | Mean (SD) per study cohort:  a) Tranexamic acid: 1.4 (1.6)  b) Tranexamic acid + desmopressin : 1.0 (0.1)  c) Desmopressin: 0.9 (0.2)  d) Control: 1.0 (0.1) |
| Pleym (2004) | Mean (SD) per study cohort:  a) Desmopressin: 1.1 (0.2)  b) Placebo: 1.1 (0.1) |
| Leino (2010) | Mean (SD) per study cohort:  a) Desmopressin 0.4mcg/kg: 0.9 (0.2)  b) Desmopressin 0.2mcg/kg: 1.0 (0.2)  c) Placebo: 0.9 (0.2) |
| Bignami (2016) | Median (Interquartile range) per study cohort:  a) Desmopressin : 0.90 (0.79-1.08)  b) Placebo: 0.96 (0.82-1.1)  Number of patients with preoperative creatinine >1.5mg/dL by study cohort:  a) Desmopressin: 7 (11%)  b) Placebo: 5 (7.6%) |
| Clagett (1995) | Number (%) of patients with creatinine >/=2mg/dL by study cohort:  a) Desmopressin: 5 (12%)  b) Placebo: 8 (19%) |
| Manno (2011) | Mean (SD) per study cohort:  a) Desmopressin: 1.0 (0.3)  b) Placebo: 1.0 (0.2)  Estimated glomerular filtration (mL/min) per study cohort:  a) Desmopressin : 94.2 (22.8)  b) Placebo: 89.4 (21.3) |
| Vafaee (2020) | Mean (SD) per study cohort:  a) Desmopressin: 0.90 (0.19)  b) Placebo: 0.95 (0.24) |

# Additional Figures

## Figure S1. Risk of bias assessments for new studies using Cochrane Risk of Bias 2.0 criteria.

Desmopressin compared with placebo or usual care

*Need for red blood cell transfusion*

*Total blood loss*

*Transfusion volume*

*Any bleeding*


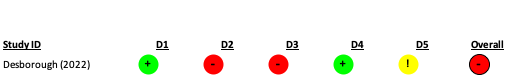


*Reoperation due to bleeding*


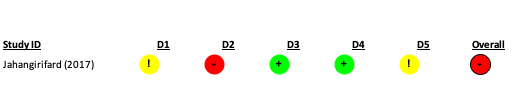


*Myocardial infarction*


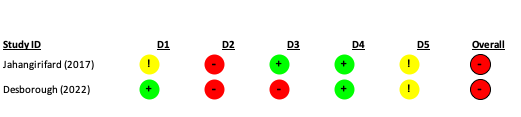


*Stroke*


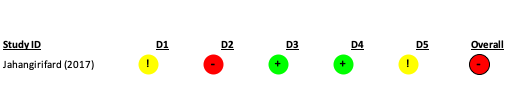


*Venous thromboembolism*


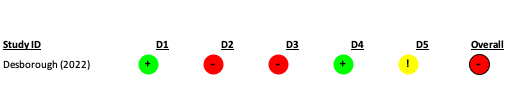


*Clinically important hypotension*


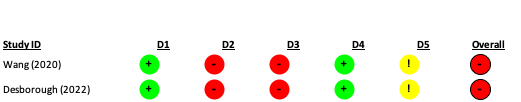


*Hyponatremia*


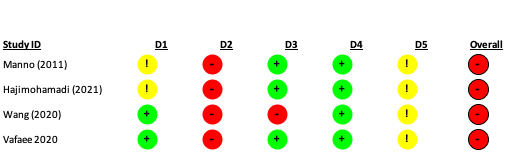


*Nausea*


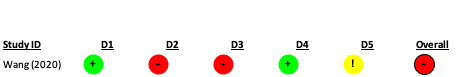


Desmopressin compared with tranexamic acid

*Total volume of blood loss*


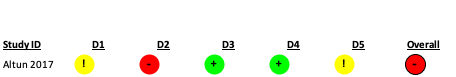


## Figure S2. Risk of bias assessments for studies included in the previous review, mapped using Cochrane Risk of Bias 2.0.


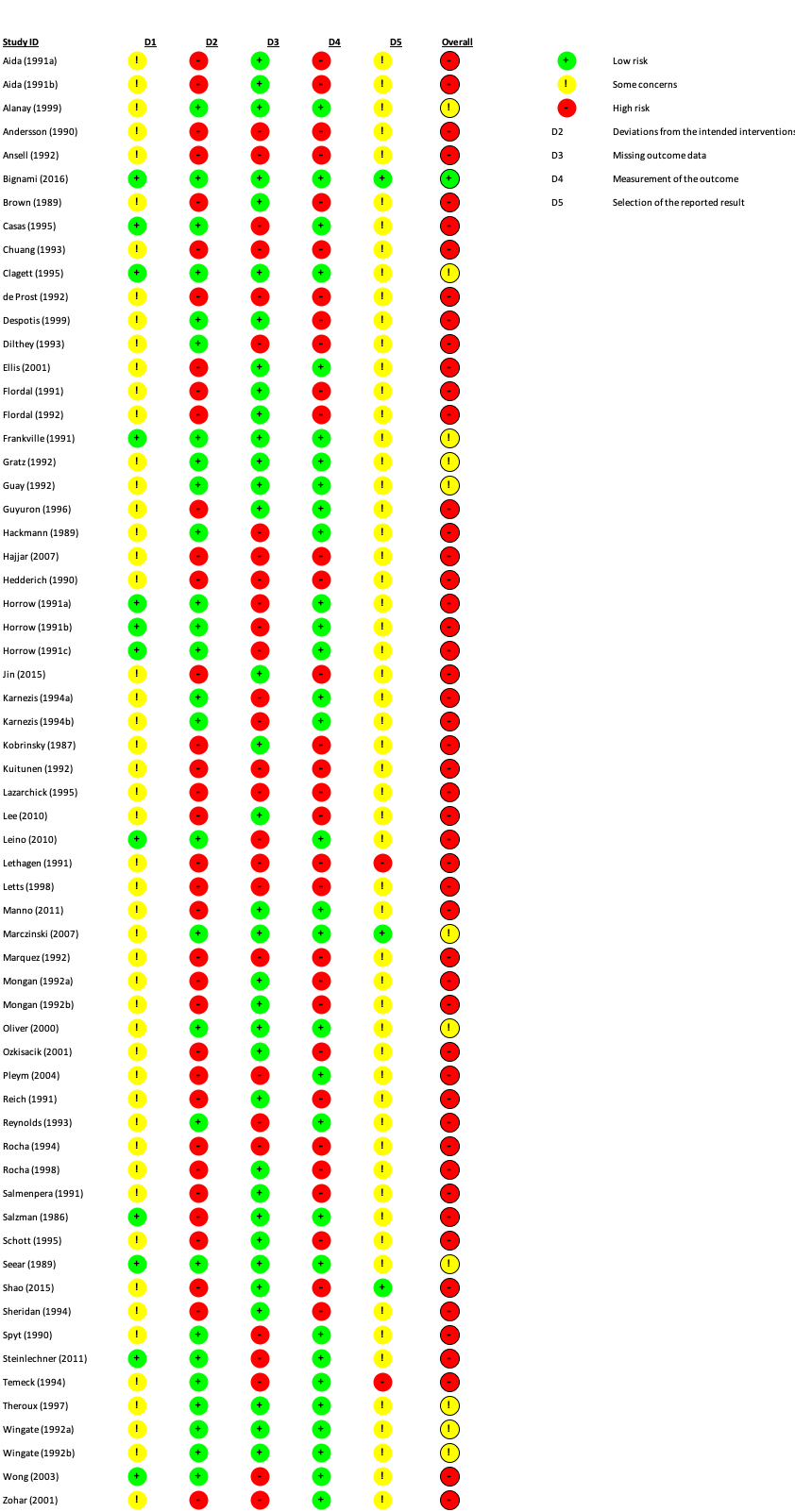


## Figure S3. Risk of bias of 2 randomly selected studies using Cochrane Risk of Bias 2.0.

## Figure S4. Risk of bias of 2 randomly selected studies applying mapping of Cochrane Risk of Bias 2.0.

## Figure S5. Trial sequential analysis of desmopressin compared with placebo or usual care on the number of participants needing red blood cell transfusion.


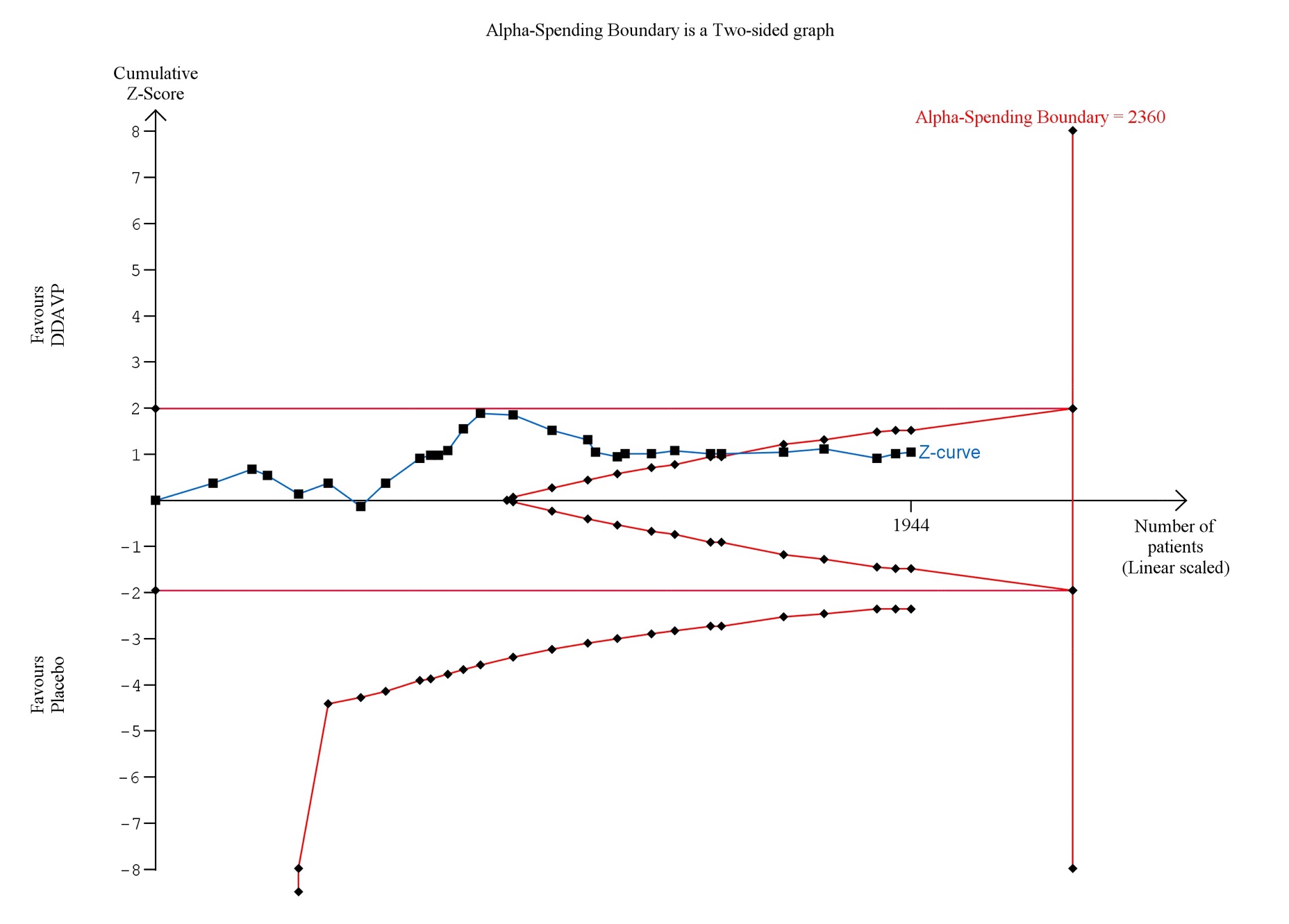


## Figure S6. Trial sequential analysis of desmopressin compared with placebo or usual care on total volume of blood loss.


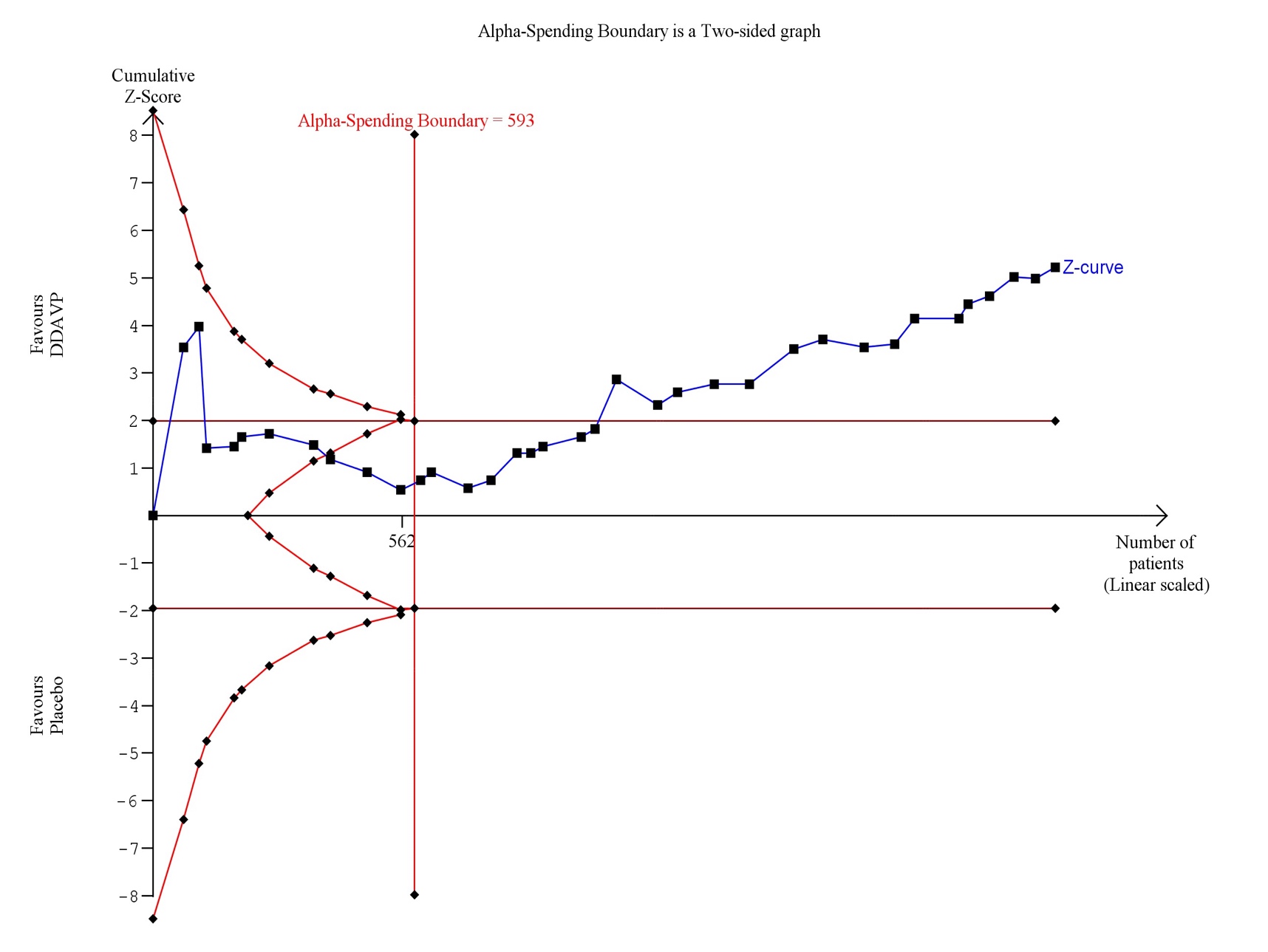


## Figure S7. Trial sequential analysis of desmopressin compared with tranexamic acid on total volume of blood loss.


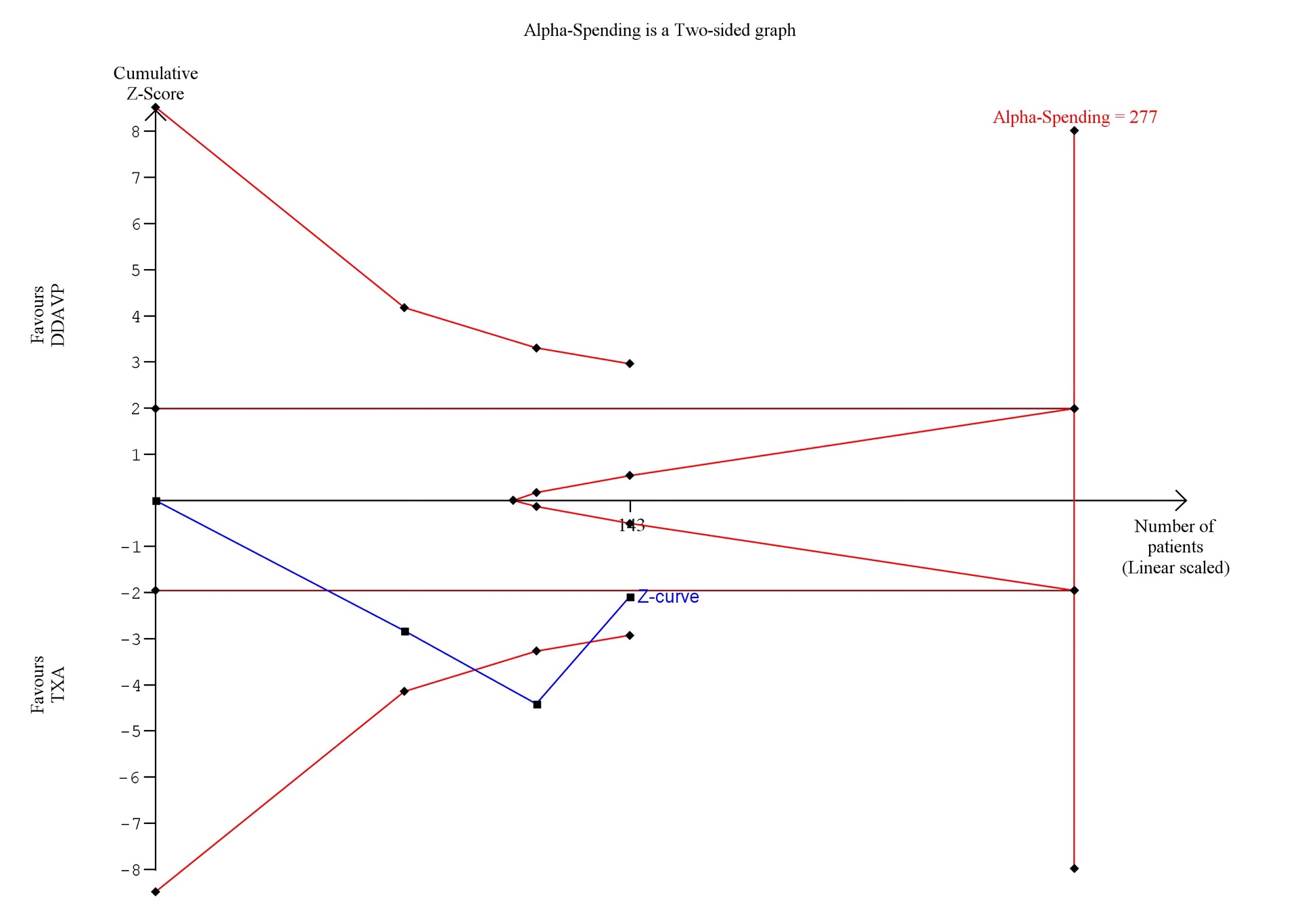


## Figure S8. Trial sequential analysis of desmopressin compared with placebo or usual care on units of red blood cells transfused.


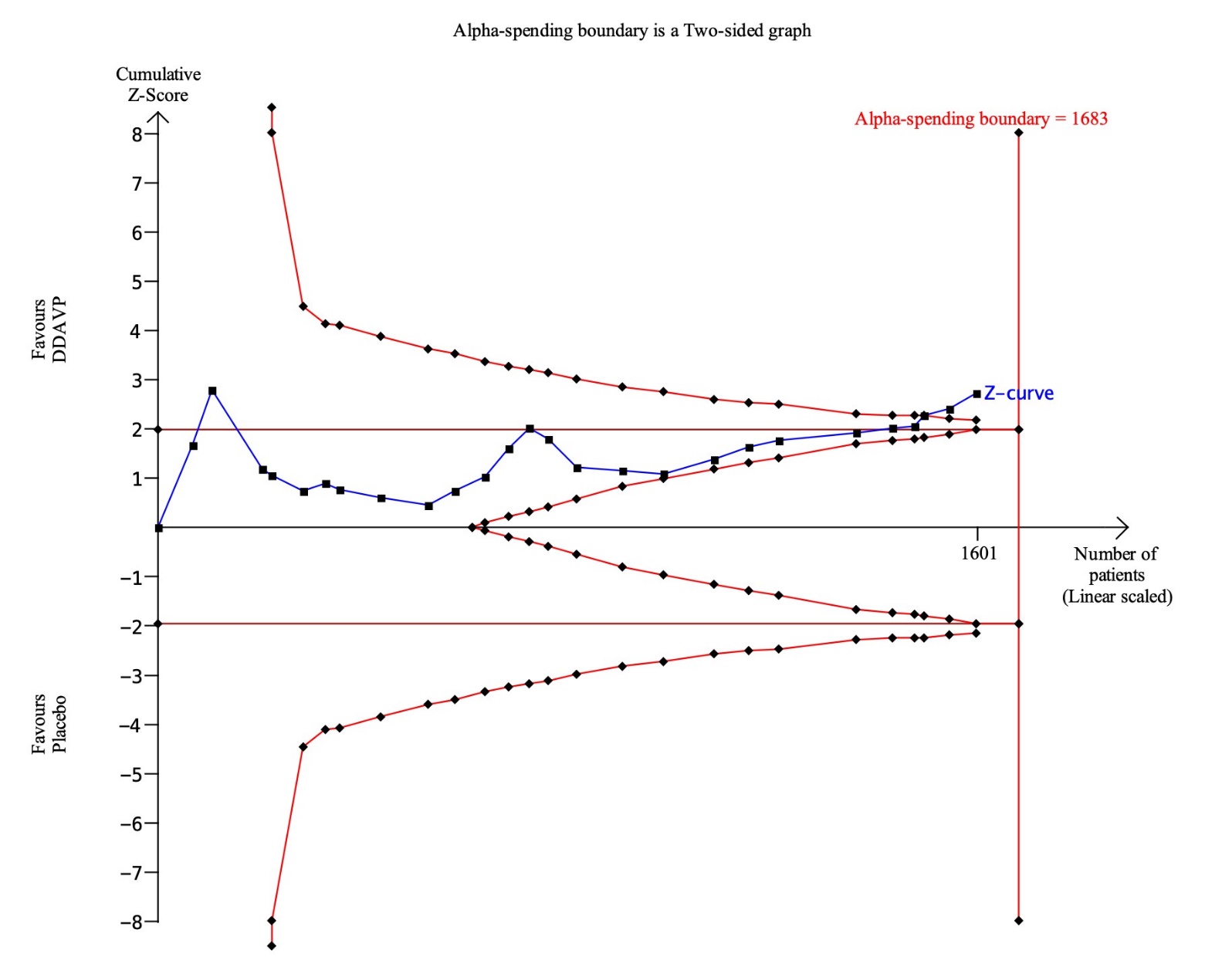


## Figure S9. Trial sequential analysis of desmopressin compared with tranexamic acid on units of red blood cells transfused.


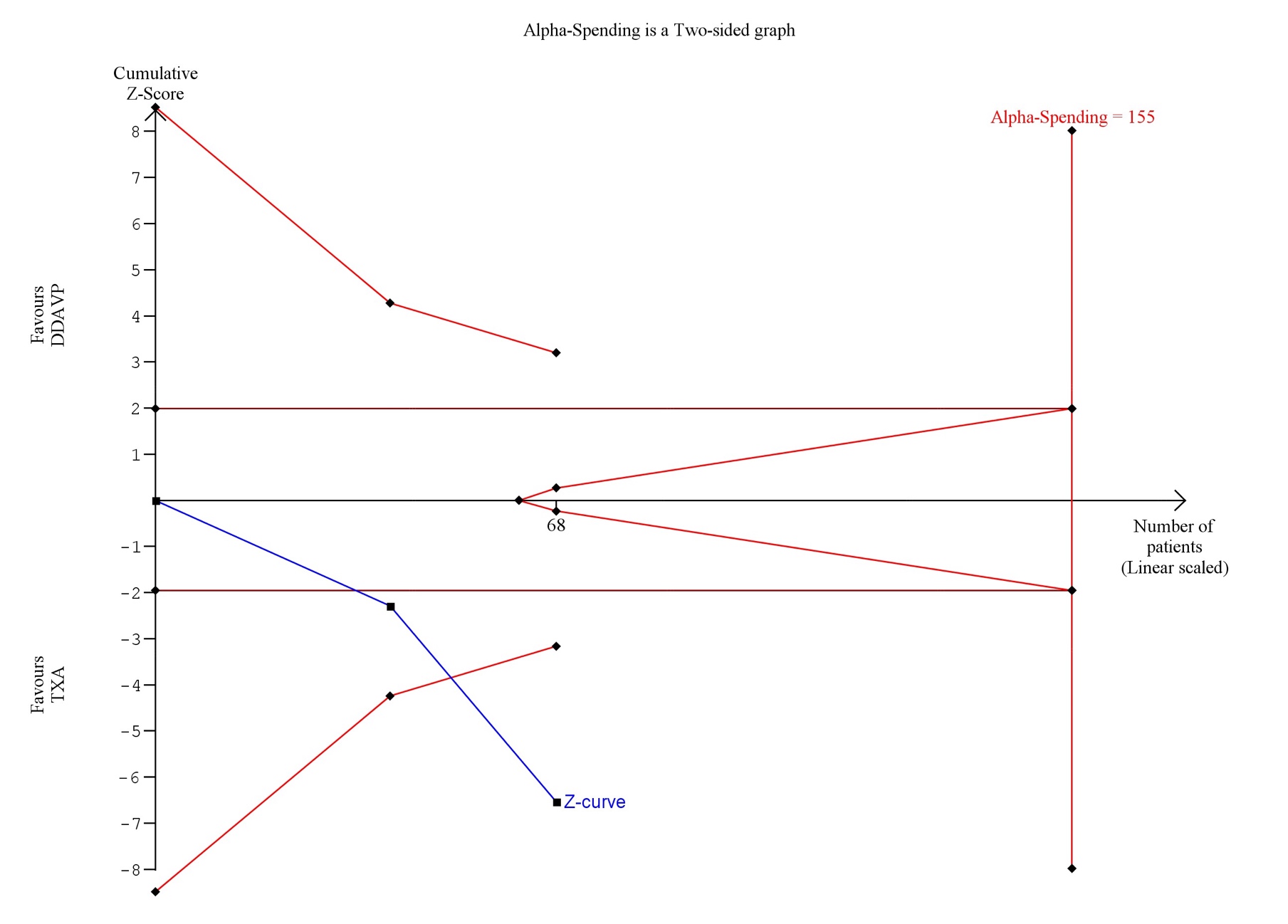


## Figure S10. Trial sequential analysis of desmopressin compared with placebo or usual care on any bleeding.


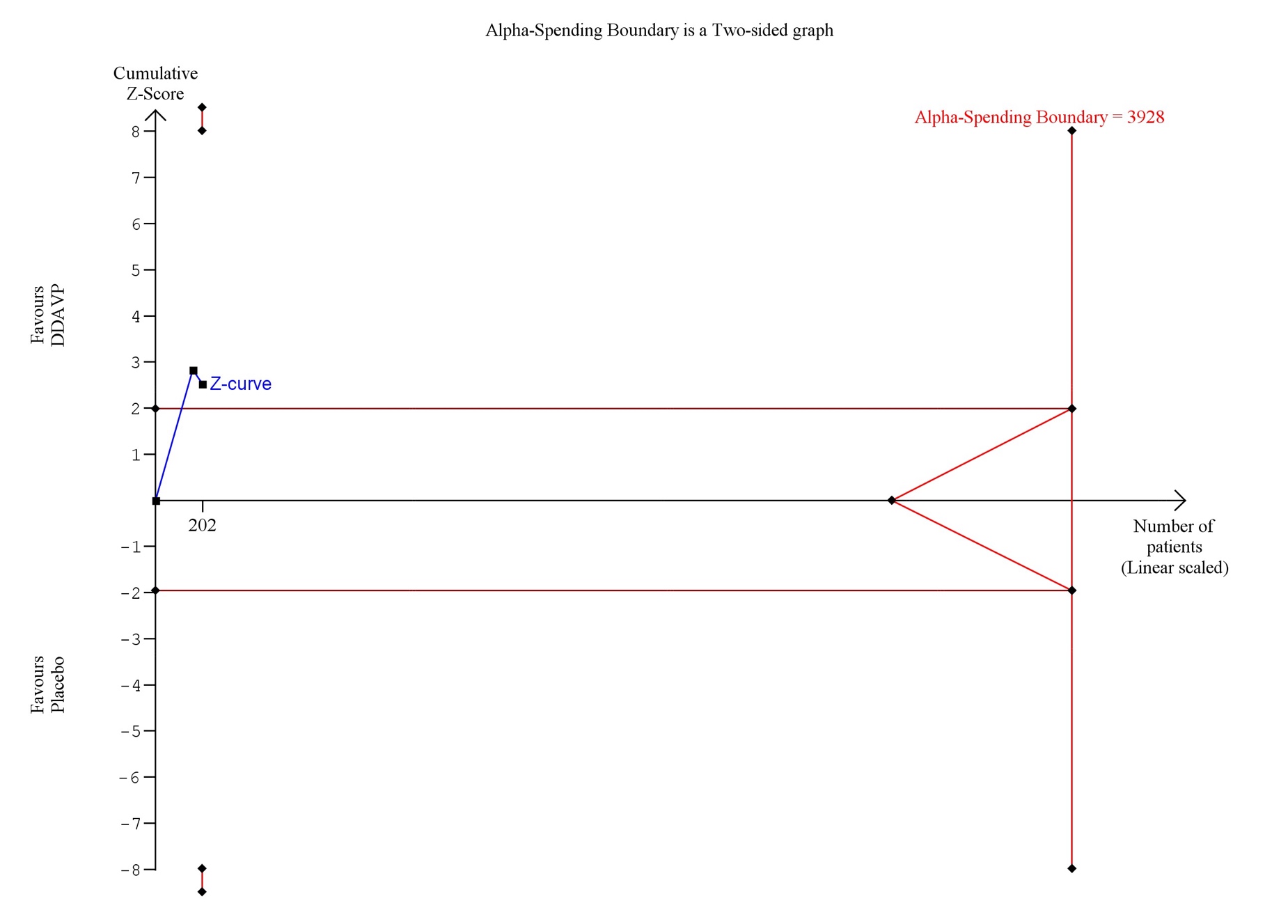


## Figure S11. Trial sequential analysis of desmopressin compared with placebo or usual care on reoperation due to bleeding.


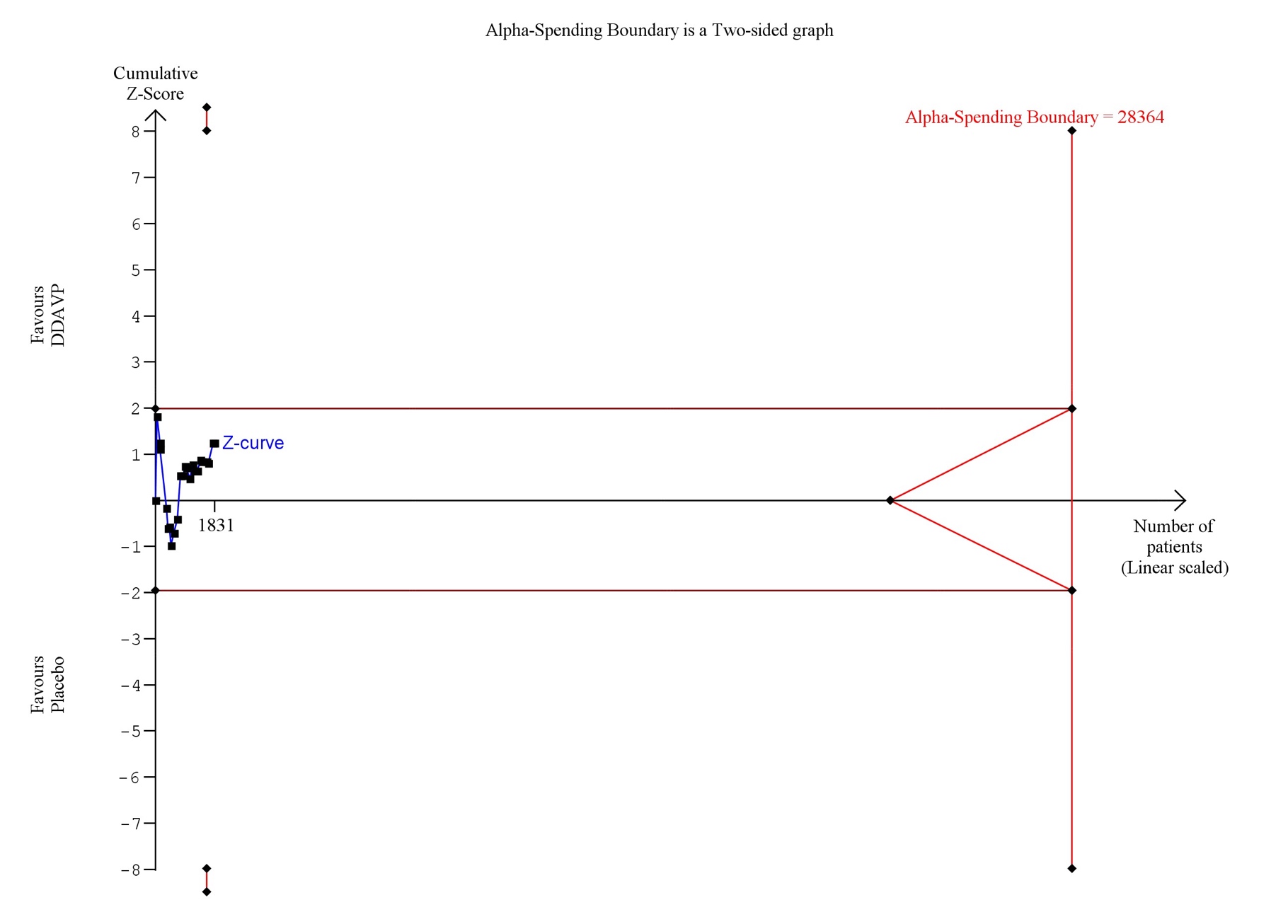


Figure S12. Funnel plot of desmopressin to placebo or usual care for outcome of number of participants who received a red cell transfusion amongst participants.


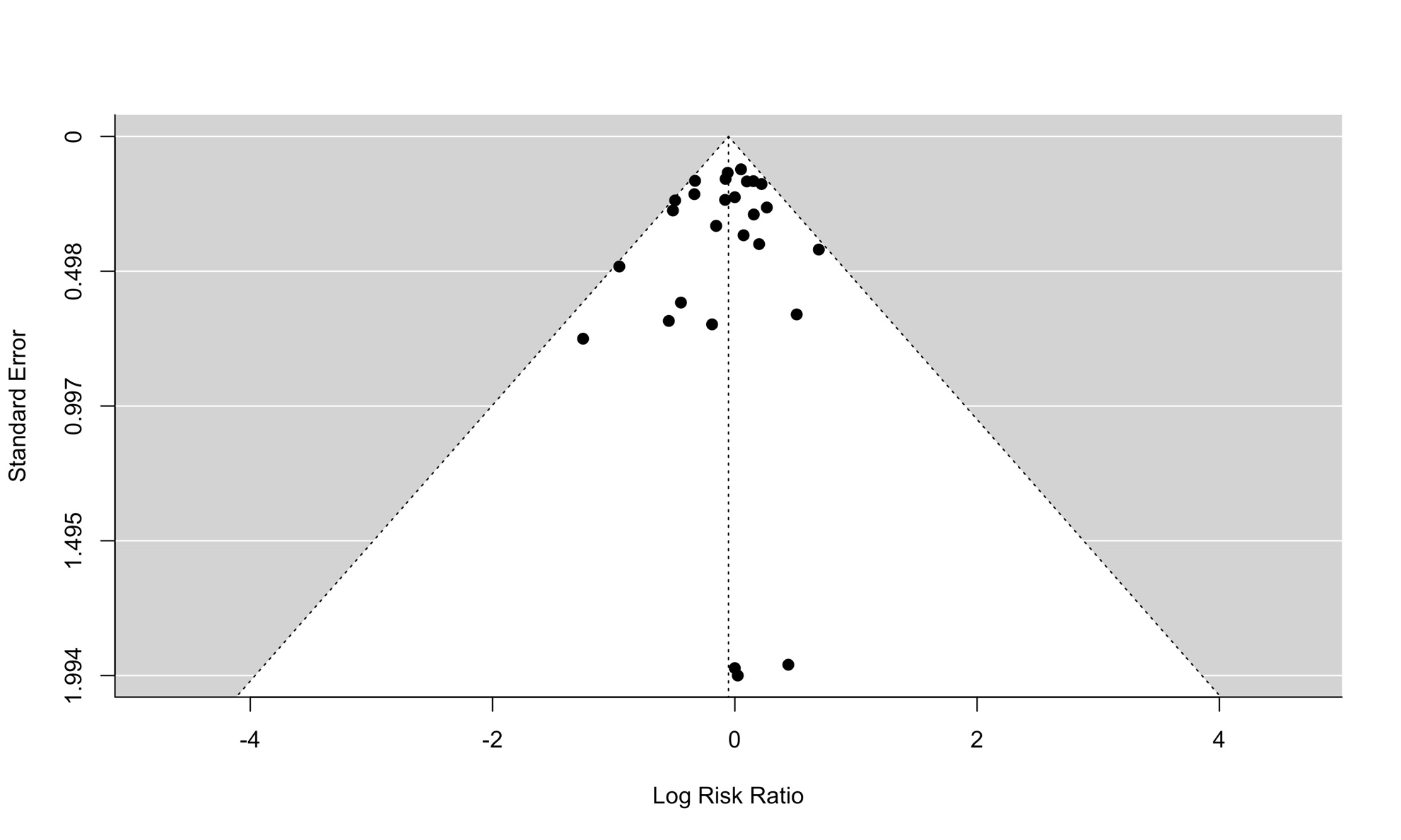


## Figure S13. Funnel plot of desmopressin to placebo or usual care for outcome of total volume of blood loss.


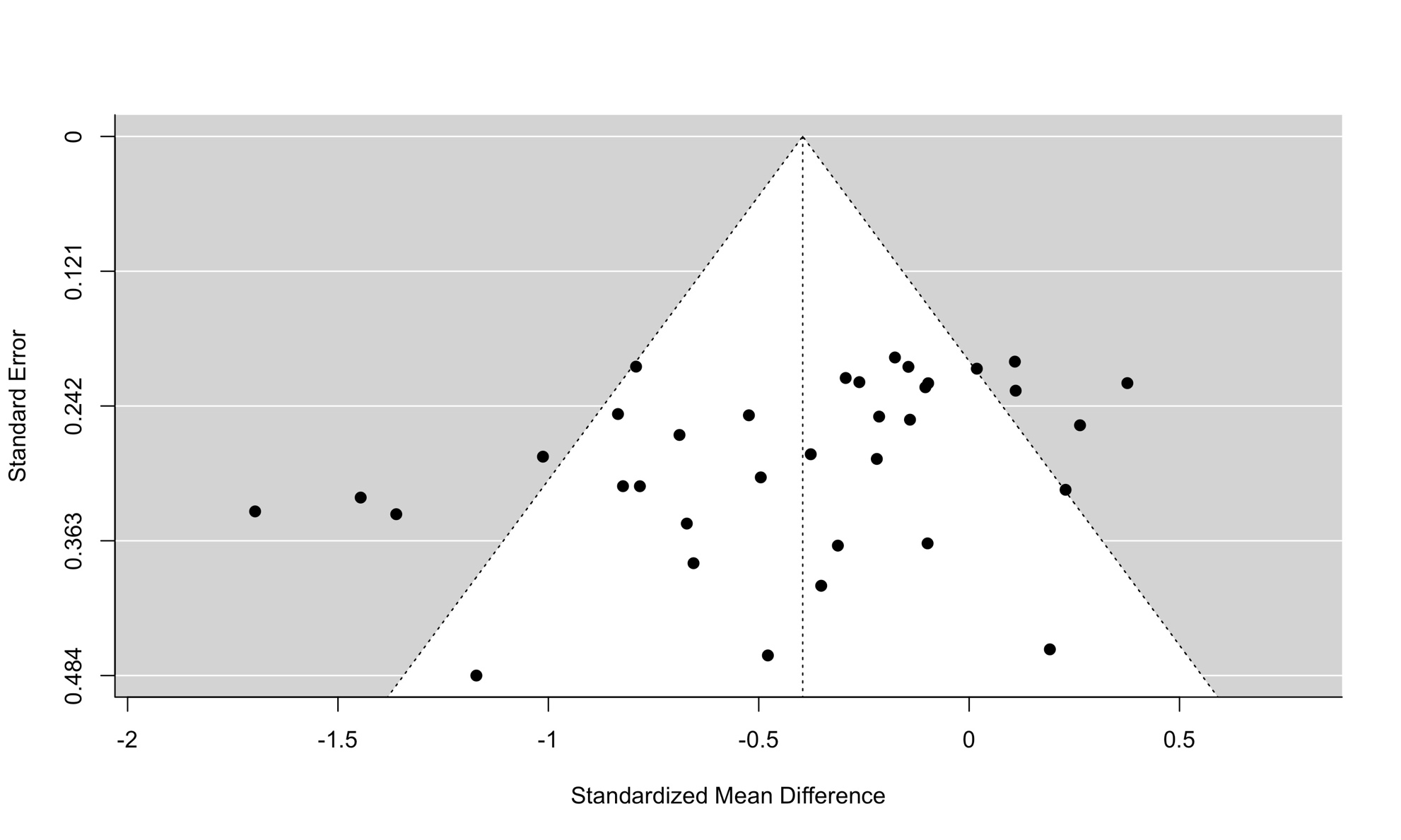


## Figure S14. Funnel plot of desmopressin to placebo or usual care examining the outcome of units of red blood cell transfusion.


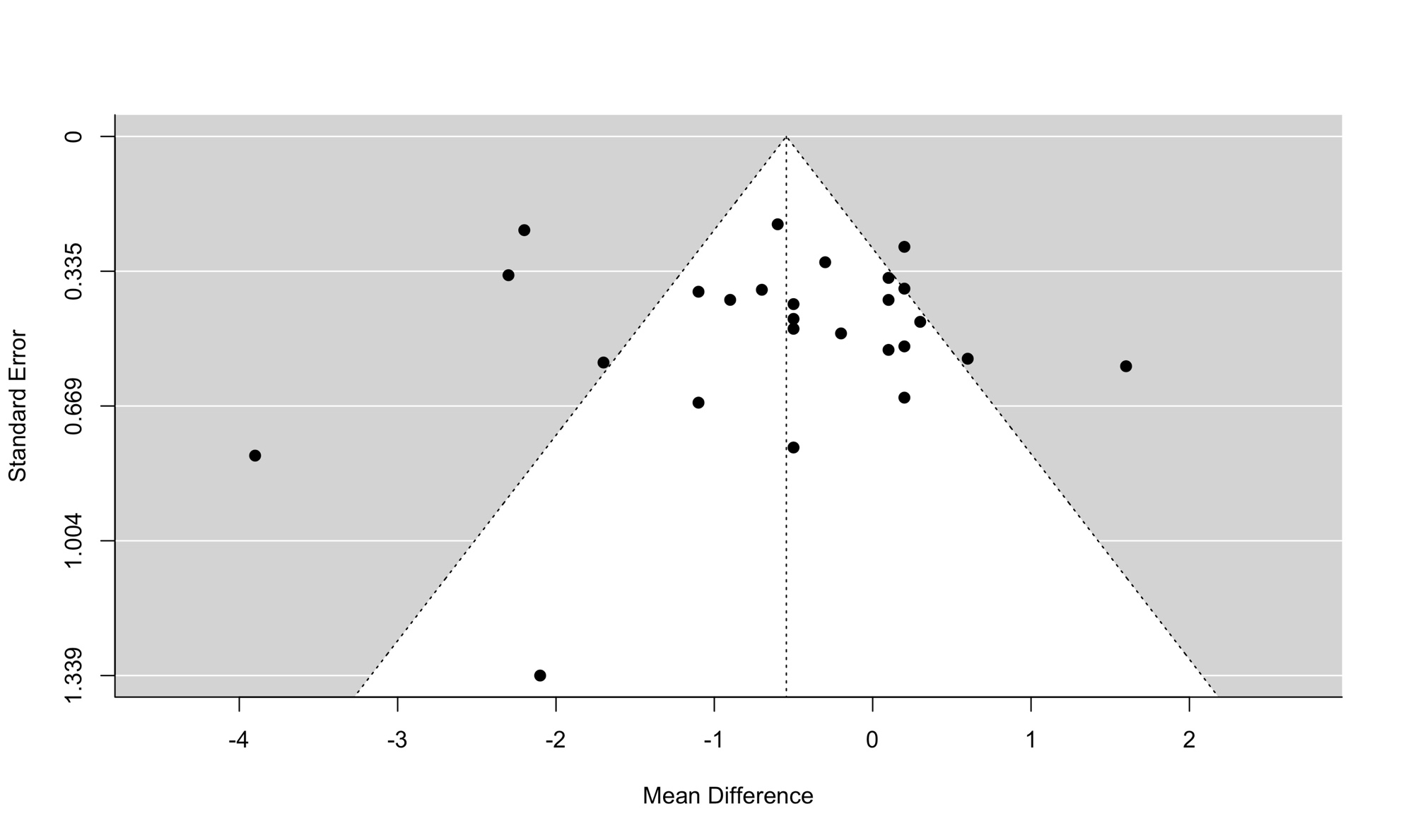


## Figure S15. Funnel plot of desmopressin to placebo or usual care examining the outcome of any bleeding.


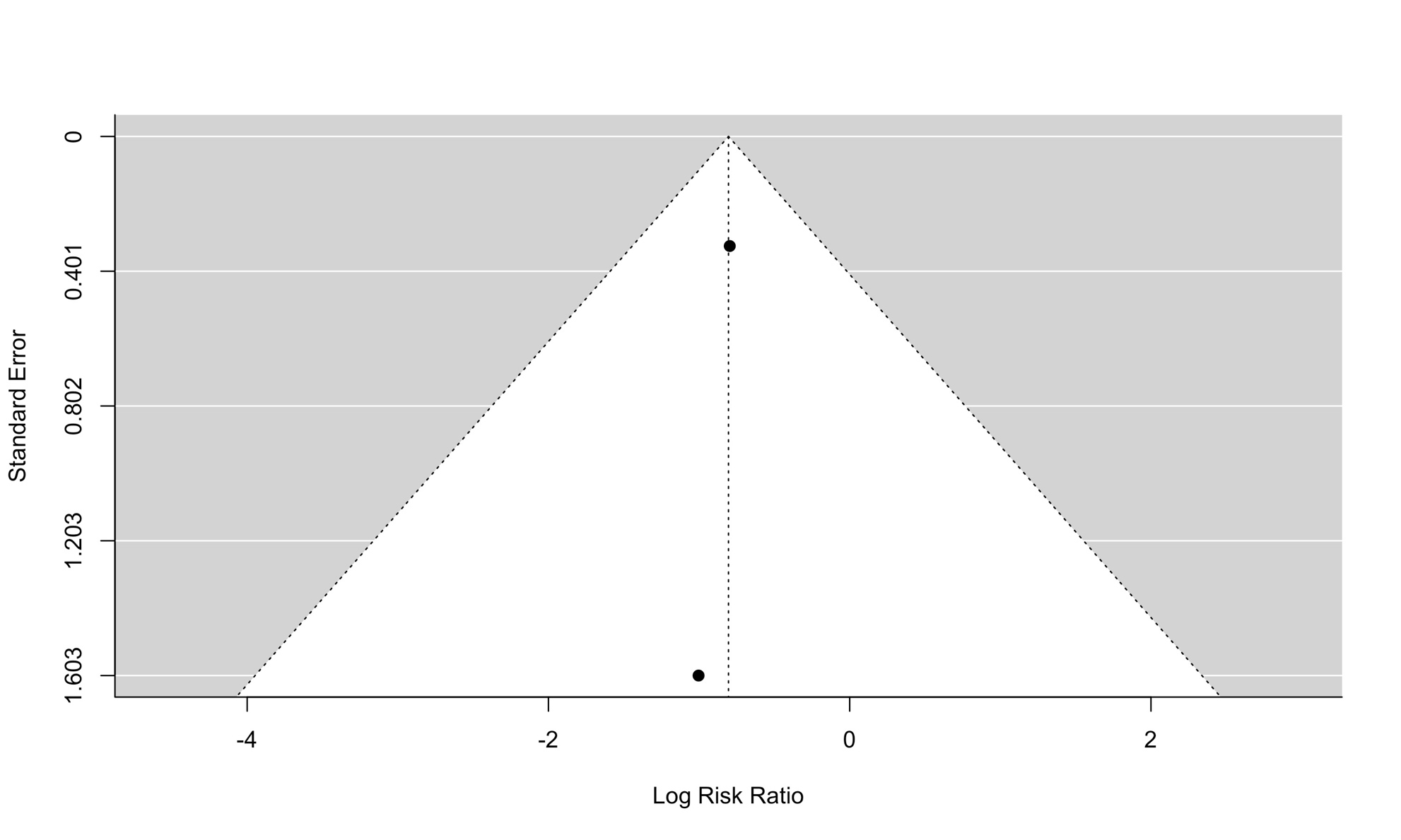


## Figure S16. Funnel plot of desmopressin to placebo or usual care examining the outcome of reoperation due to bleeding.


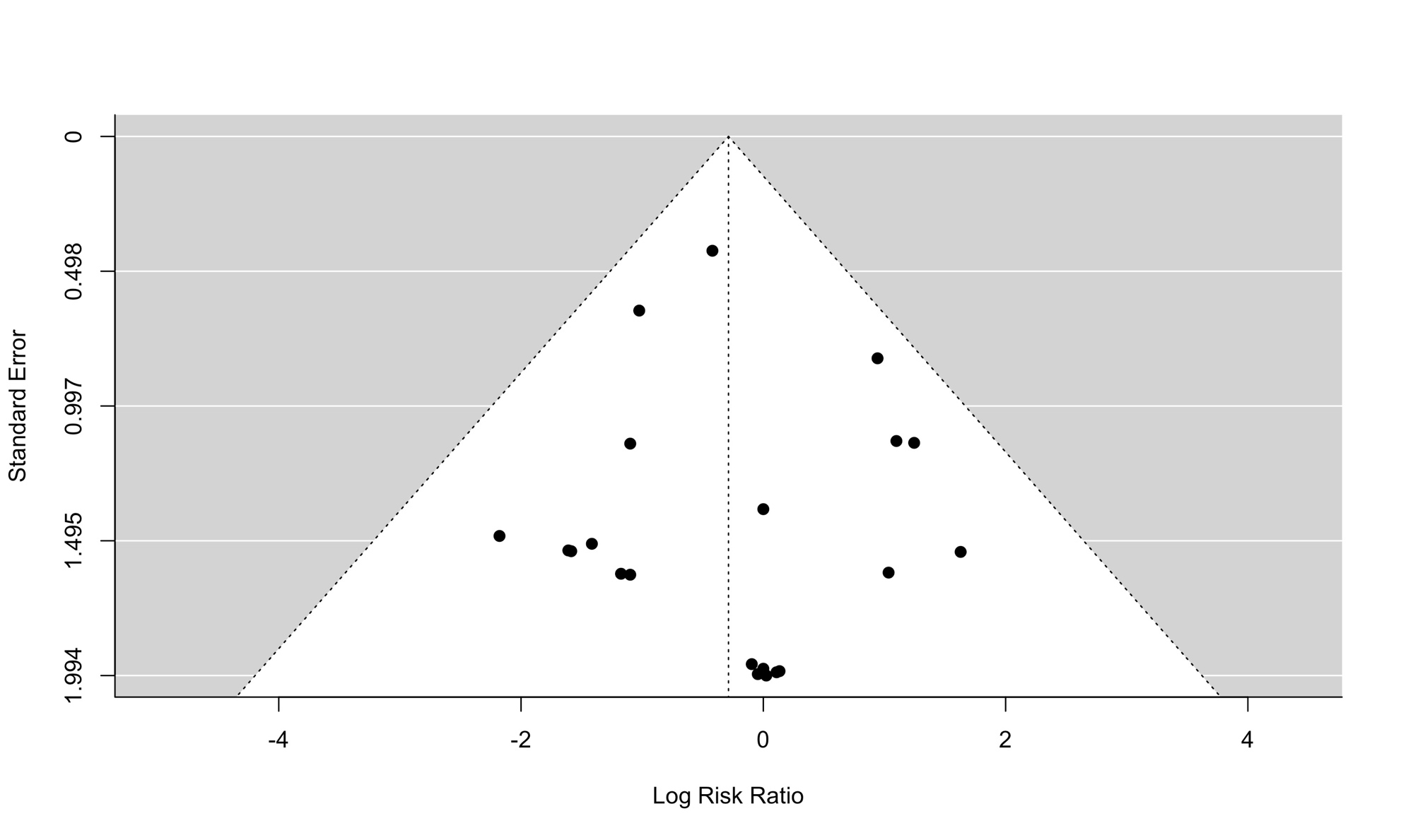


Figure S17. Funnel plot of desmopressin to placebo or usual care examining the outcome of myocardial infarction.


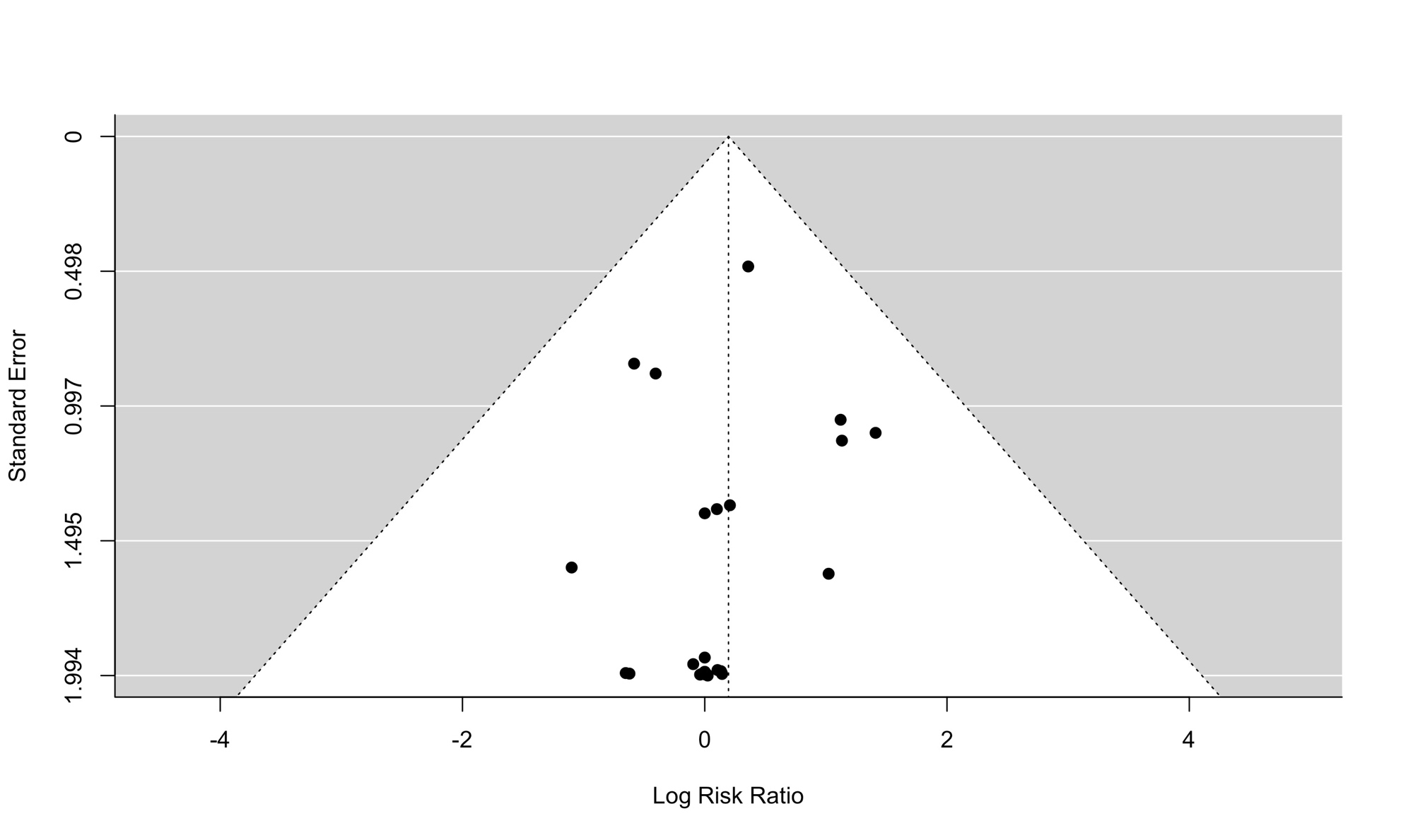


## Figure S18. Funnel plot of desmopressin to placebo or usual care examining the outcome of stroke.


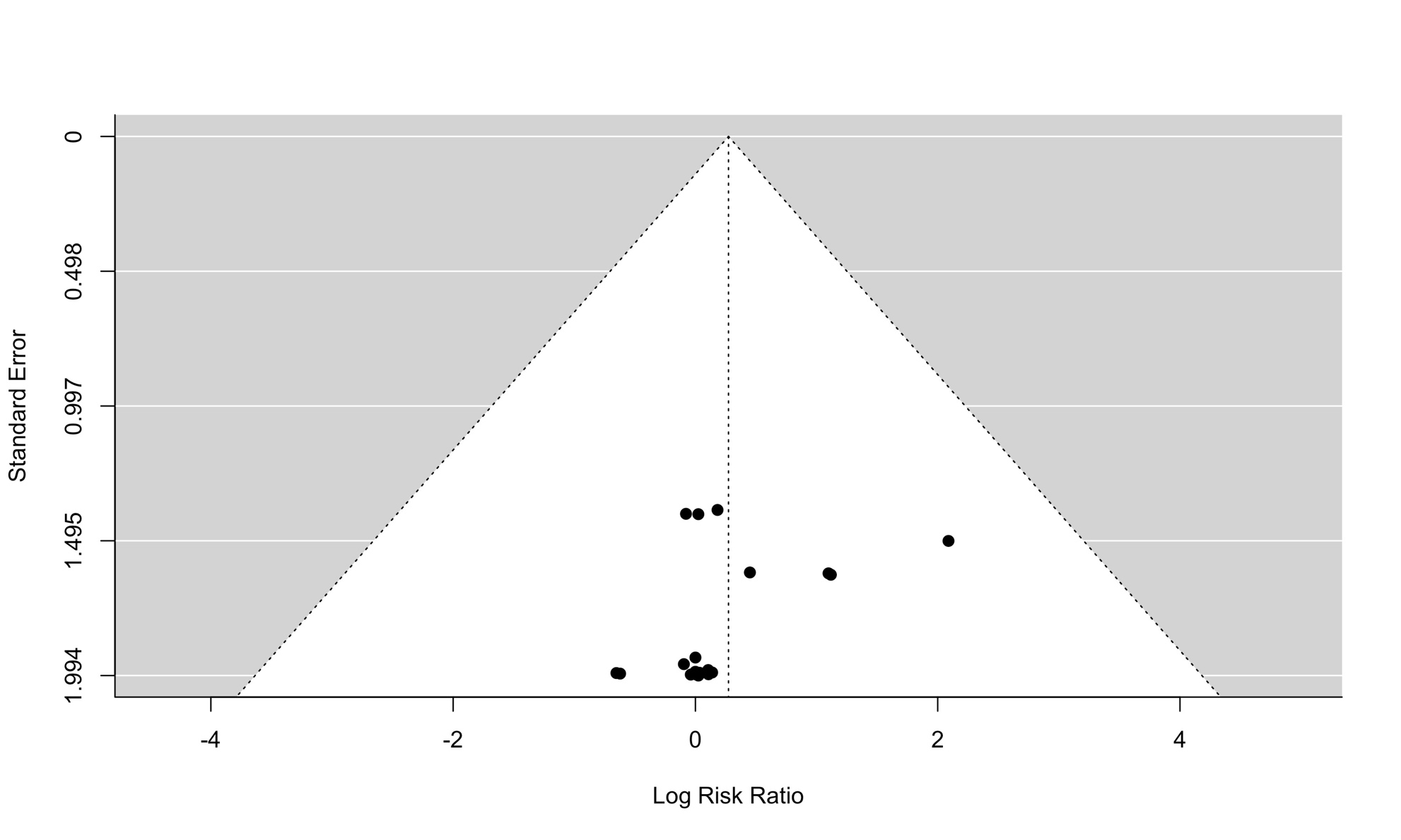


## Figure S19. Funnel plot of desmopressin to placebo or usual care examining the outcome of clinically important hypotension.


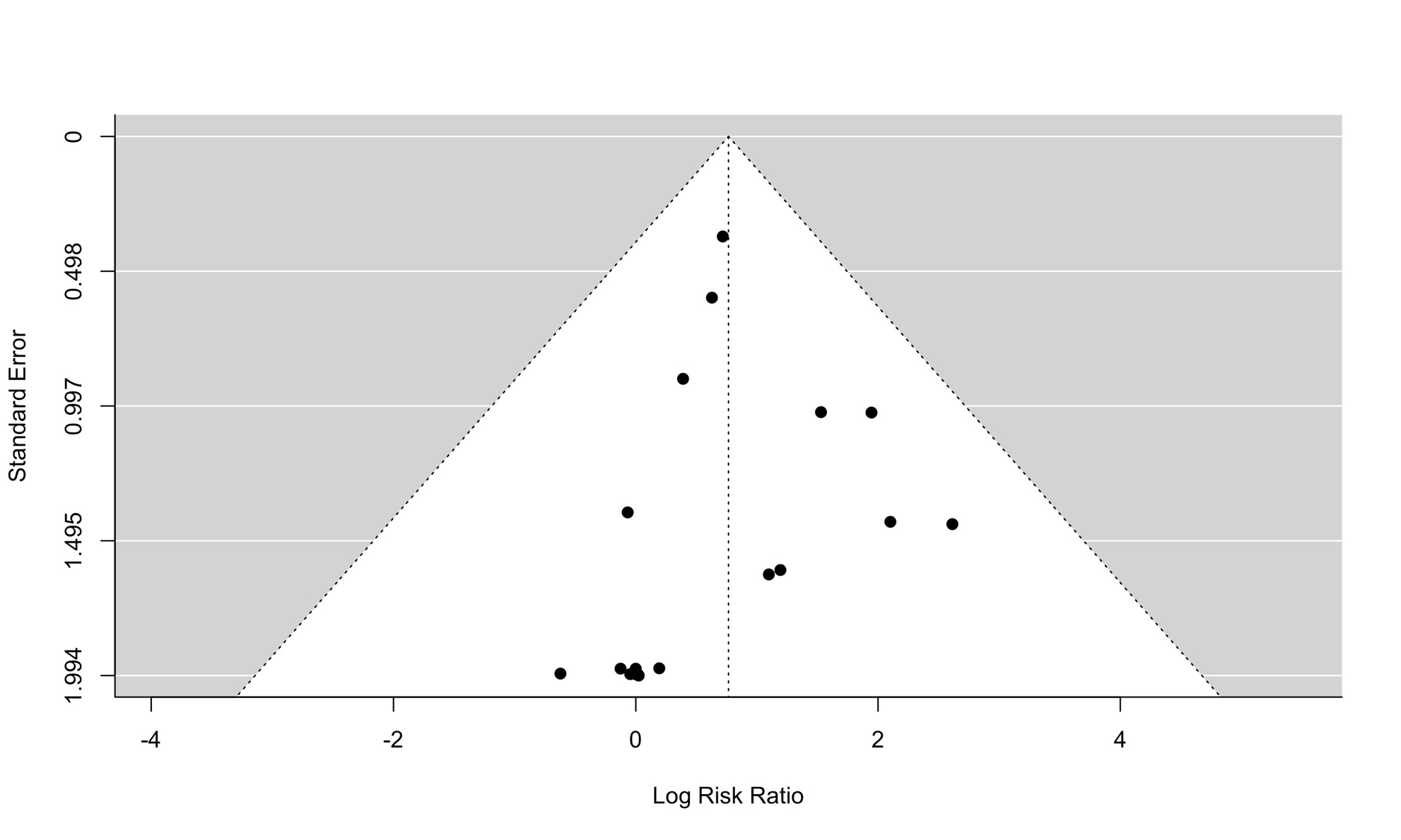


## Figure S20. Funnel plot of desmopressin to placebo or usual care examining the outcome of venous thromboembolism.


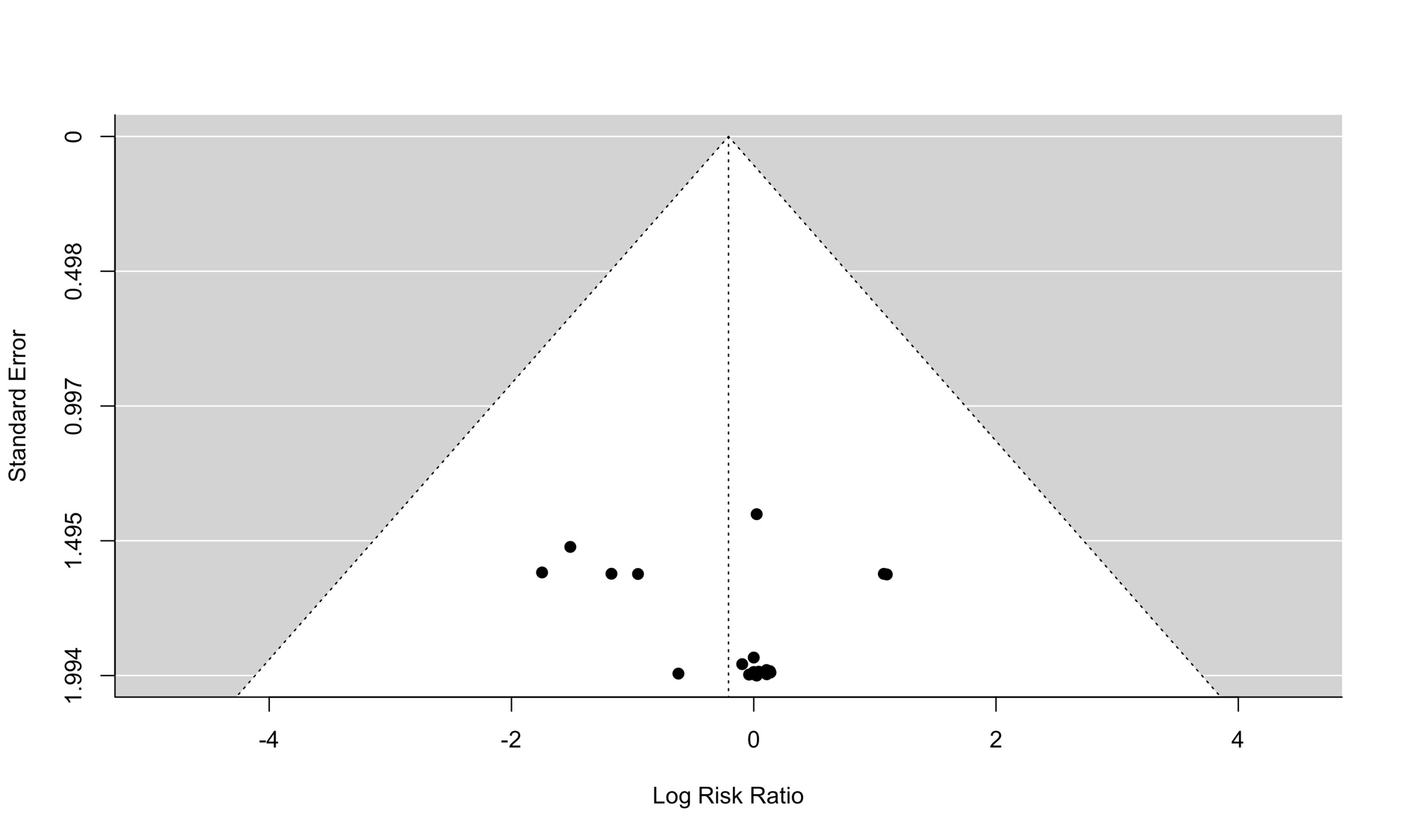


## Figure S21. Funnel plot of desmopressin to placebo or usual care examining the outcome of hyponatremia (dichotomous).


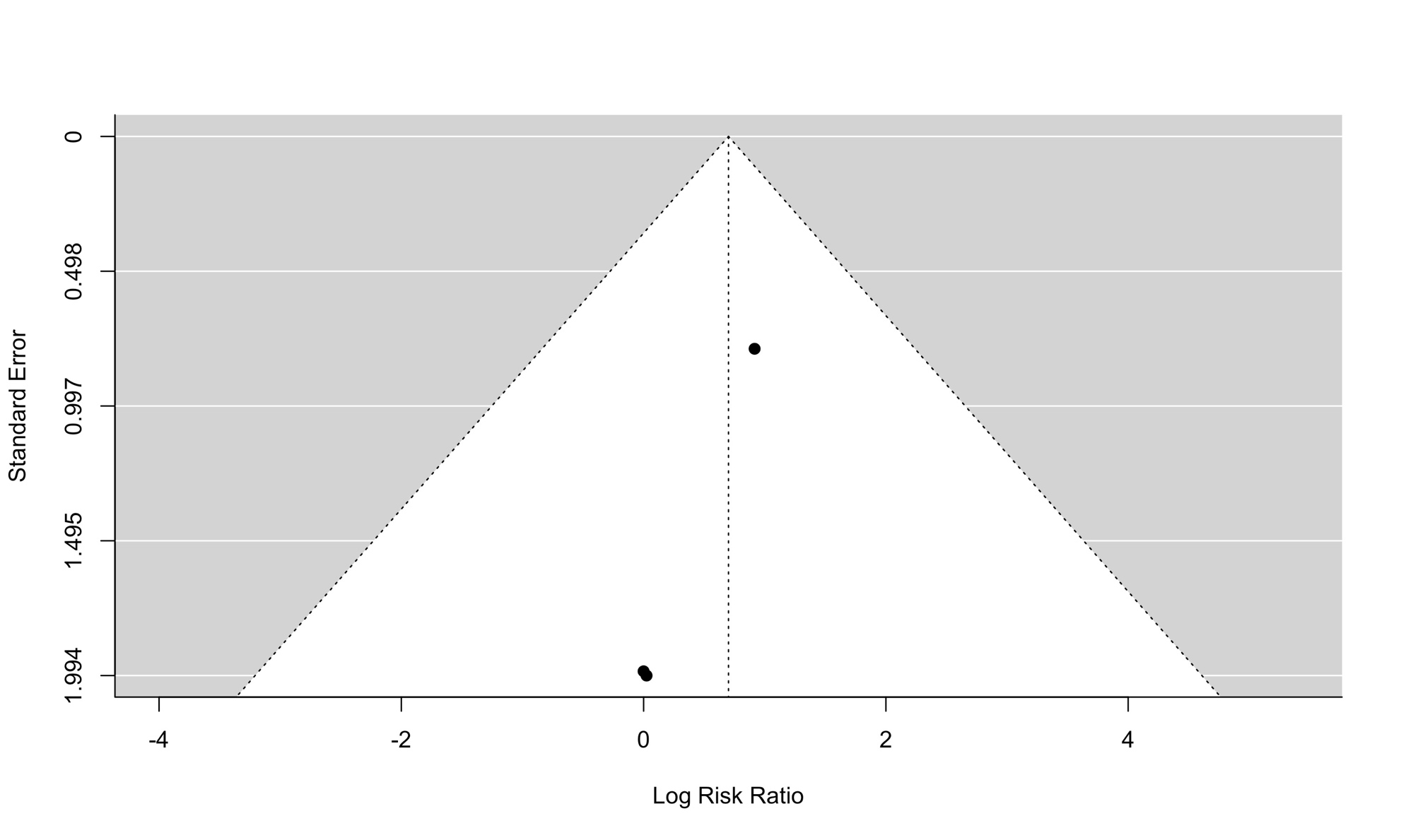


## Figure S22. Funnel plot of desmopressin to placebo or usual care examining the outcome of post-procedural serum sodium.


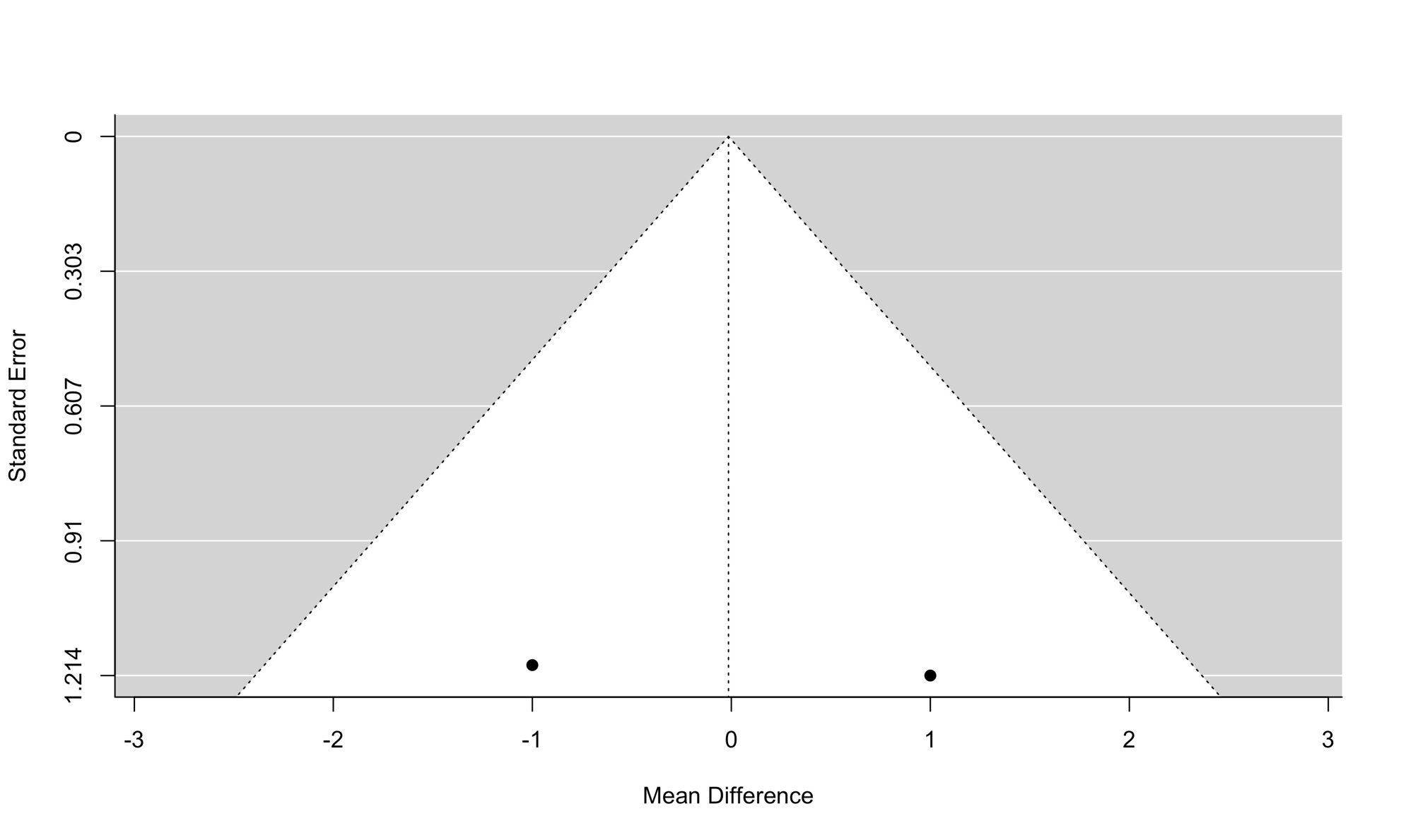


## Figure S23. Funnel plot of desmopressin to tranexamic acid for outcome of number of participants who received a red cell transfusion amongst participants.


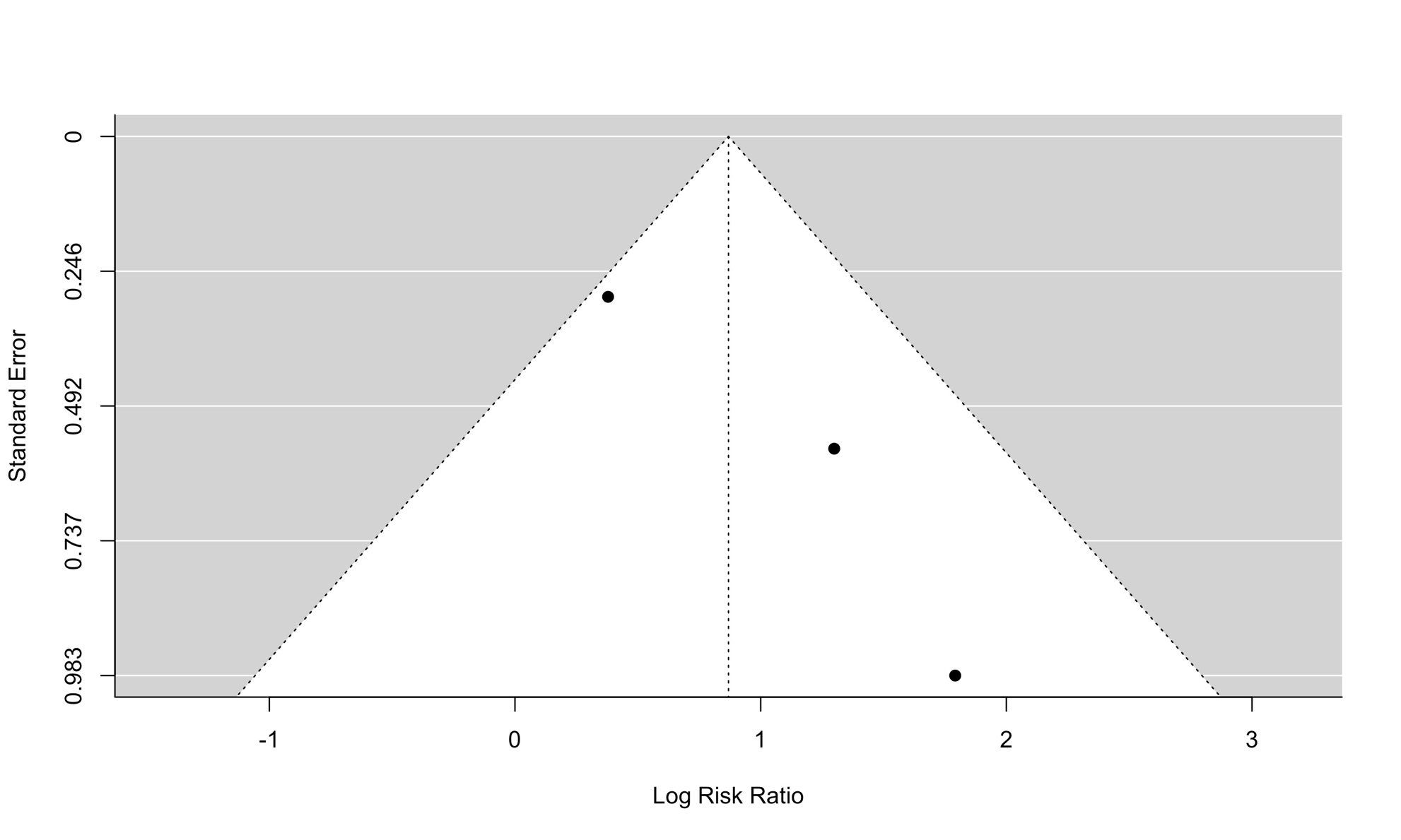


## Figure S24. Funnel plot of desmopressin to tranexamic acid for outcome of total volume of blood loss.


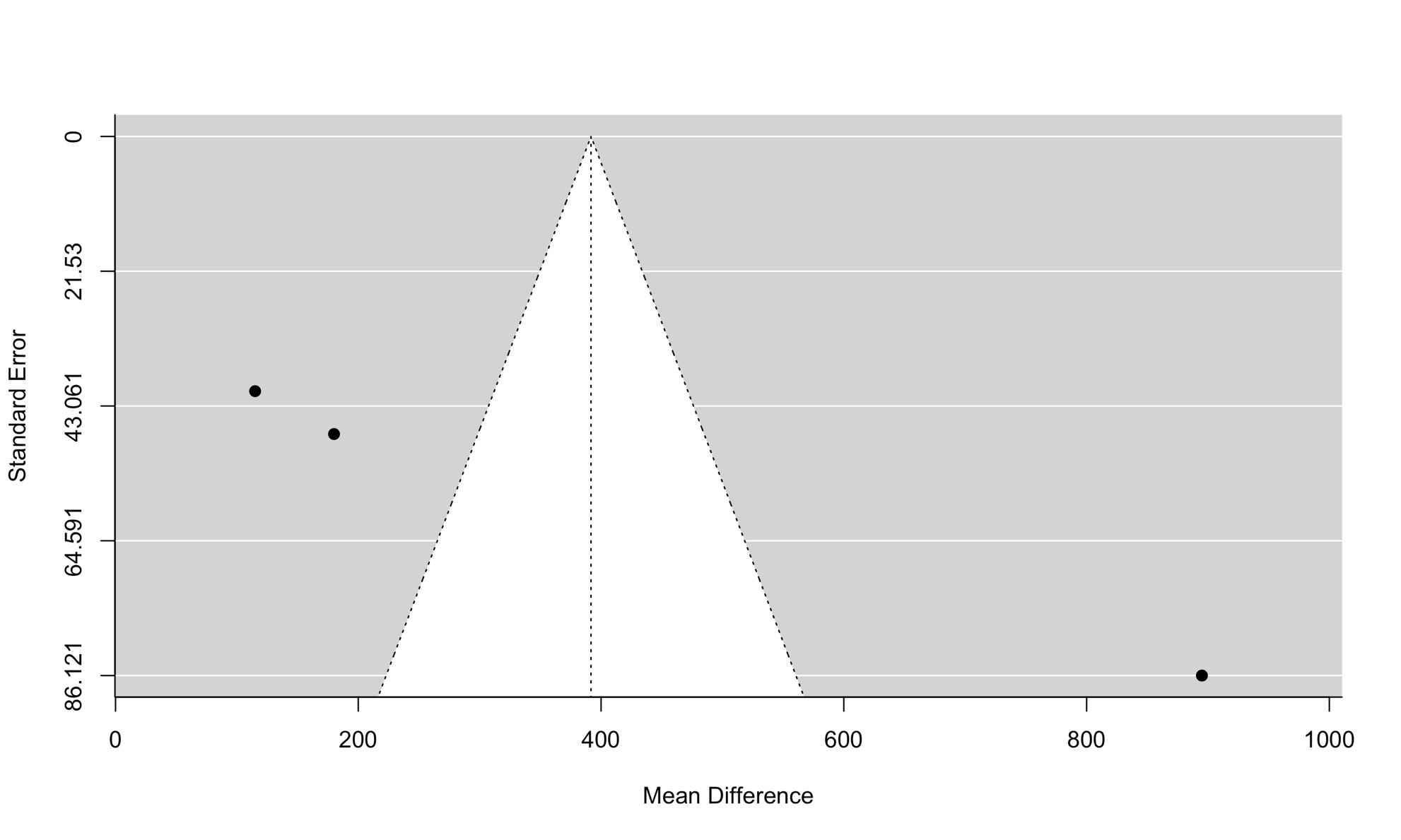


## Figure S25. Funnel plot of desmopressin to tranexamic acid examining the outcome of units of red blood cell transfusion.


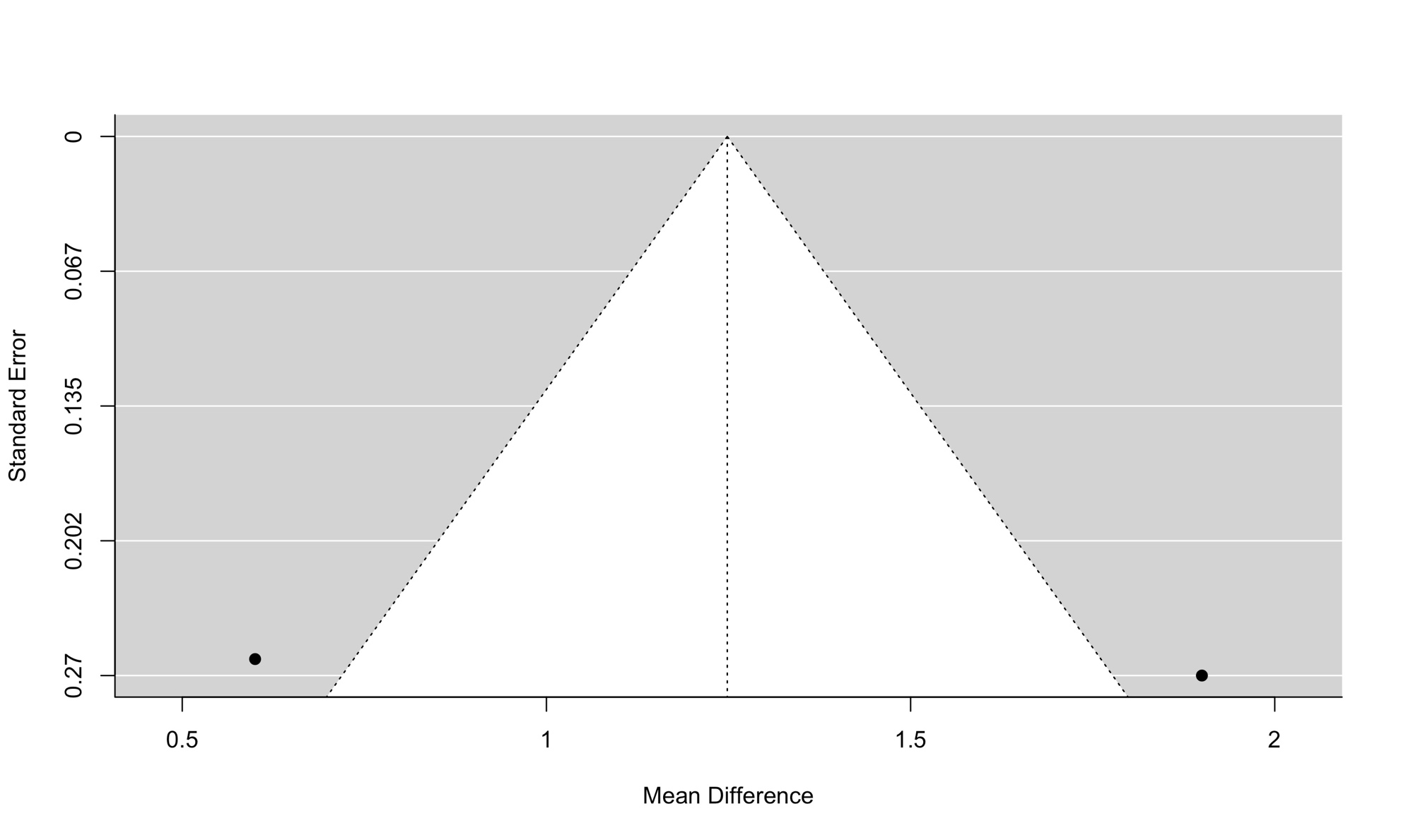


## Figure S26. Funnel plot of desmopressin to aprotinin examining the outcome of reoperation due to bleeding.


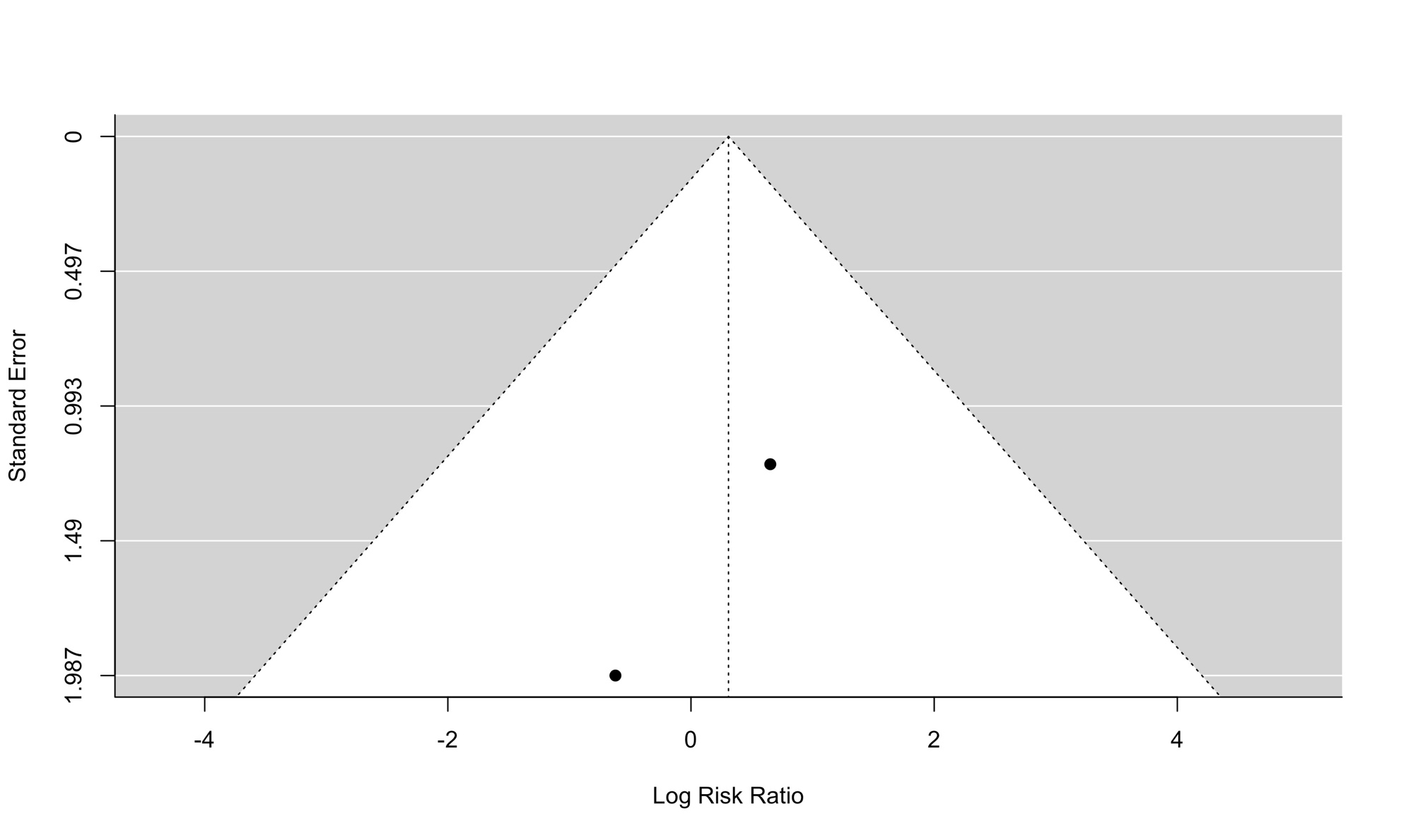


## Figure S27. Funnel plot of desmopressin to aprotinin examining the outcome of myocardial infarction.


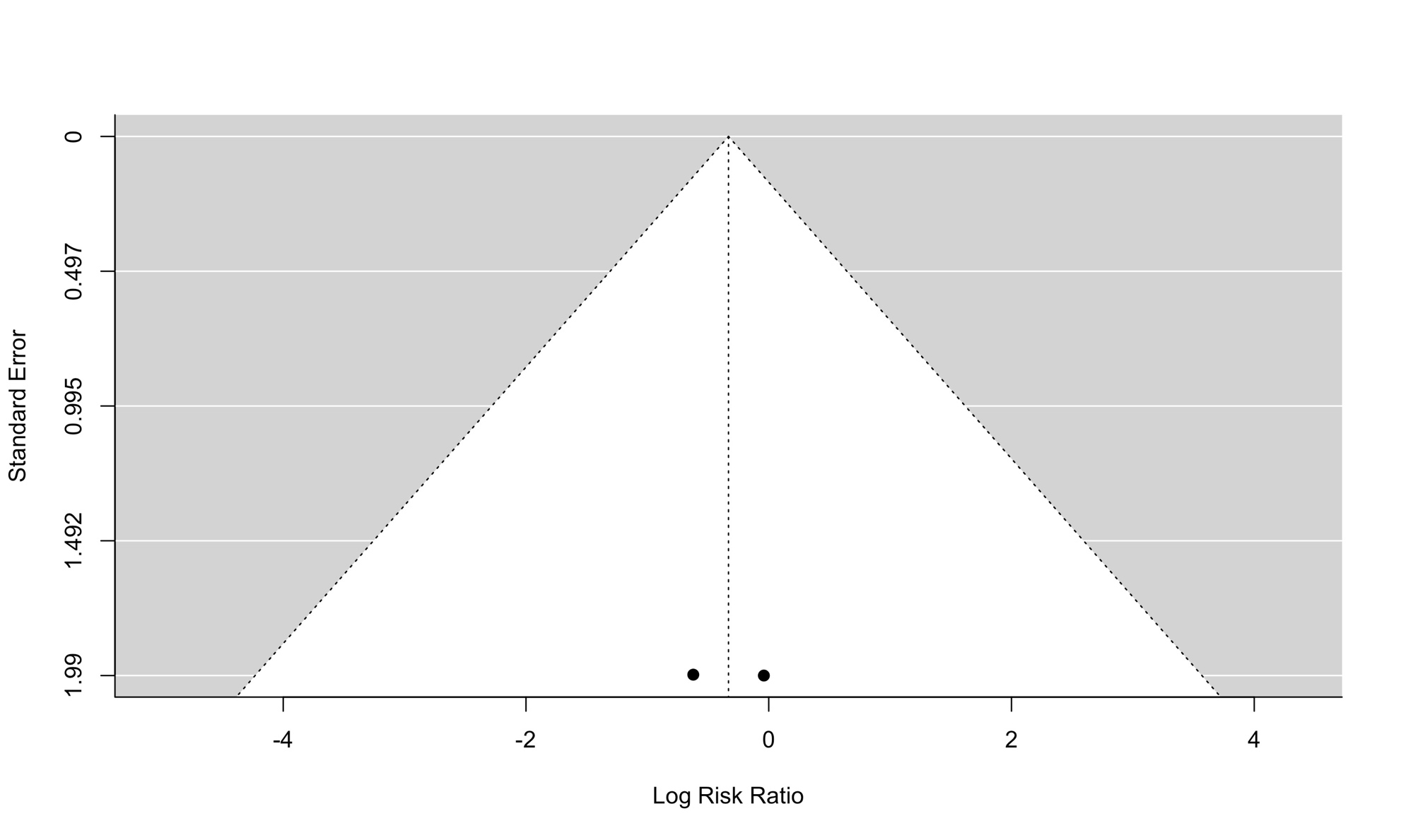


## Figure S28. Funnel plot of desmopressin to aprotinin examining the outcome of stroke.


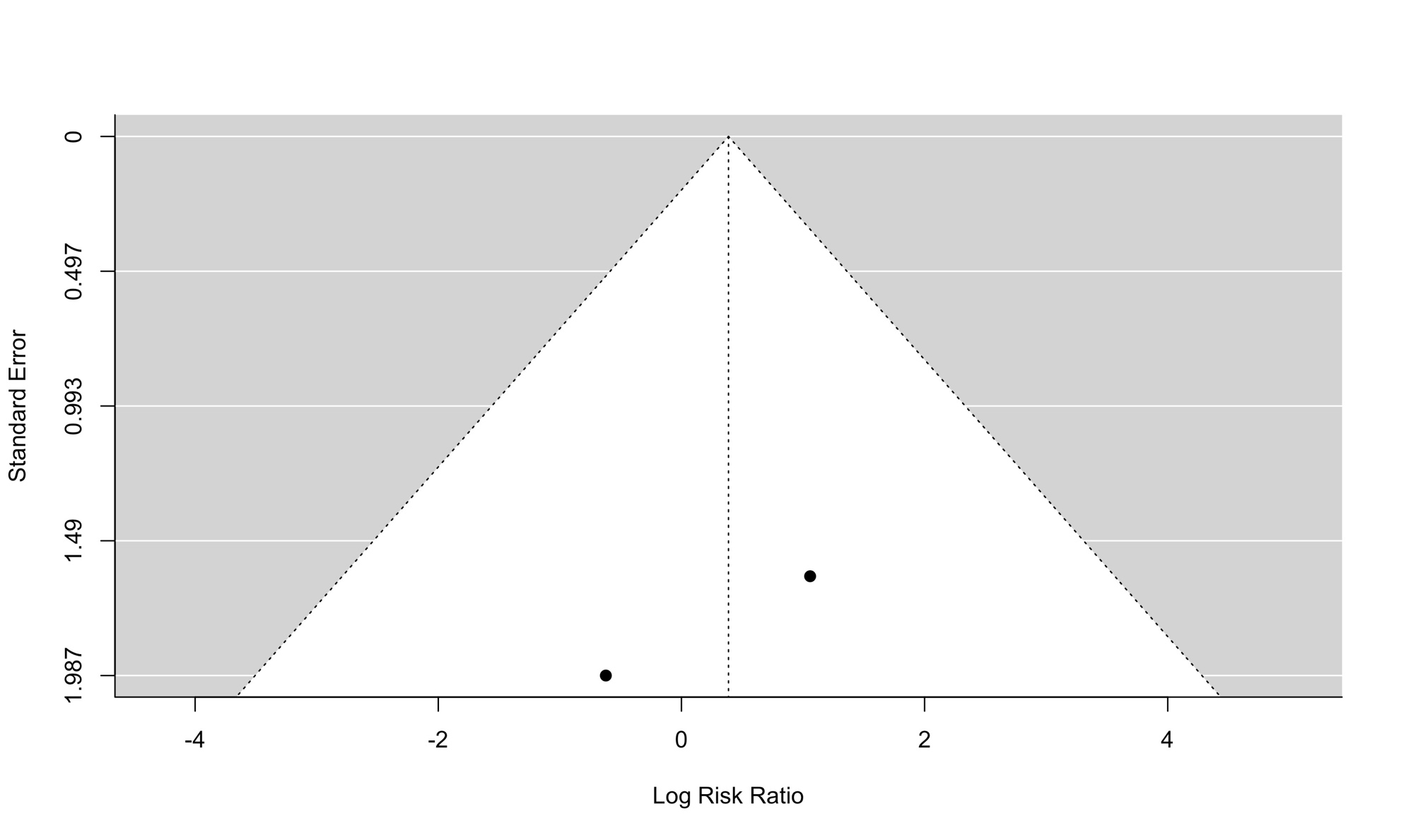


## Figure S29. Funnel plot of desmopressin to aprotinin examining the outcome of venous thromboembolism.


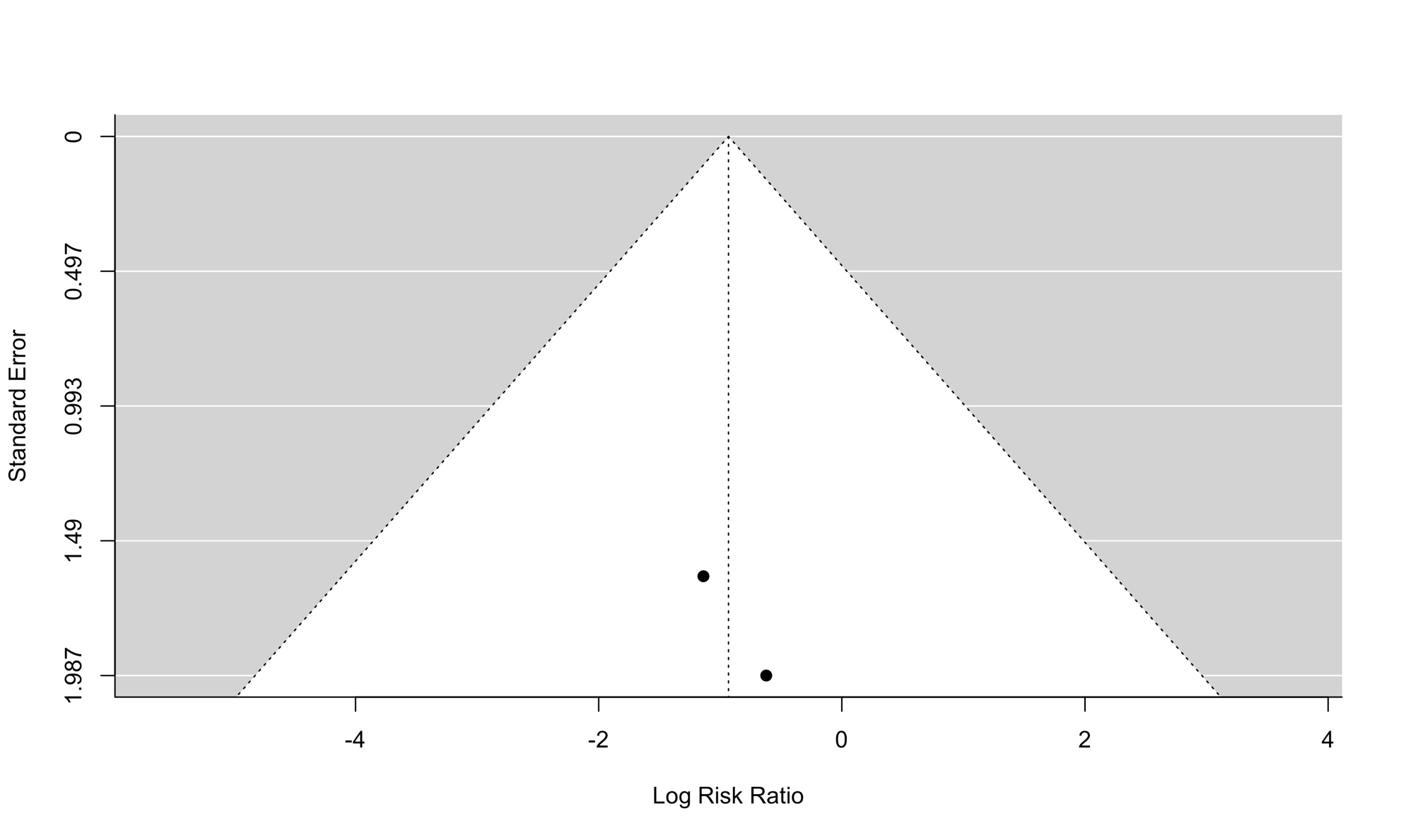


# Summary of characteristics of 15 studies that meet eligibility criteria but were available in the form of trial registries or abstracts that did not provide relevant data.

| Trial name or title | Transdermal Estrogen Provides Comparable Effectiveness with Desmopressin in Preventing Uremic Bleeding in CKD Patients Undergoing Kidney Biopsy: A Pilot Randomized, Double-Blinded Controlled Trial  Authors: Wipattanakitcharoen, A; Susantitaphong, P; Katavetin, P; Praditpornsilpa, K; Eiam-Ong, S; Takkavatakarn, K.  Year: 2022 |
| --- | --- |
| Methods | Type of Study: single-centre, parallel-group, 2-arm RCT  Country where study is being performed: Thailand |
| Participants | Inclusion criteria: patients with eGFR of less than 60 mL/min/1.73m2 undergoing kidney biopsy  Exclusion criteria: not reported |
| Interventions | Intervention arm: transdermal estrogen patches (50 mcg/day) for 7 days  Comparator arm: intravenous desmopressin (0.4 mcg/kg) |
| Outcomes | Primary outcome: Incidence of post-biopsy bleeding complications (hematoma or gross hematuria)  Secondary outcomes: Hematoma size, changes in hemoglobin levels, platelet function assay by PFA-200, and adverse events |
| Notes | No response from authors after 3 attempts. |

| Trial name or title | Severe Aortic Stenosis and Acquired Von Willebrand’s Disease: The Impact of Desmopressin in Valve-Replacement Surgery  Trial registry: NCT01994330 (<https://clinicaltrials.gov/ct2/show/NCT01994330>) |
| --- | --- |
| Methods | Type of study: single-centre, parallel-group, 2-arm RCT  Country where study is being performed: Chile |
| Participants | Inclusion criteria: severe aortic stenosis defined as mean transvalvular gradient greater or equal to 40 mmHg or transvalvular area less than 1 cm^2^; scheduled for aortic valve replacement surgery  Exclusion criteria: combined surgery (plus coronary artery bypass graft or other valve replacement/plasty); infective endocarditis; previously known haemostatic disorder; previous treatment with oral anticoagulants or IIb-IIIa inhibitors (we did not exclude those on acetyl-salicylic acid) |
| Interventions | Intervention arm: 0.3 mcg per kilogram of desmopressin in 100 ml of saline, labeled as "study drug" and administered in 30 minutes a half hour before surgical incision  Comparator arm: 100 saline bottles labeled as "study drug" administered in 30 minutes a half hour before surgical incision |
| Outcomes | Primary outcome: Blood loss [ Time Frame: once patient arrives to post anesthesia care unit (approximately 6 hours after drug administration] Secondary outcomes:  - Postoperative hematocrit [ Time Frame: the morning after surgery (18-24 hours after drug administration)], hematocrit and hemoglobin in time frame mentioned  - Need of transfusion [ Time Frame: 48 hours post administration], transfusion of packaged red cells units until 48 hours after administration of study drug  - Incidence of hyponatremia [ Time Frame: 18-24 hours post administration of study drug], blood sampling for plasma sodium in specified time frame  - Von Willebrand study and protein electrophoresis [ Time Frame: the day of surgery, half hour previous to administration of study drug ], blood sampling for von Willebrand study: collagen binding activity, ristocetin factor test, coagulation factor VIII activity, von Willebrand factor antigen, ristocetin cofactor test/von Willebrand factor antigen ratio and protein electrophoresis of von Willebrand multimers |
| Notes | Received response from principal investigator, study was completed, pending data sharing. |

| Trial name or title | Desmopressin as Treatment for Postoperative Bleeding After Cardiac Surgery  Trial registry: NCT00885924 (<https://clinicaltrials.gov/ct2/show/NCT00885924>) |
| --- | --- |
| Methods | Type of study: single-centre, parallel-group, 2-arm RCT  Country where study is being performed: Norway |
| Participants | Inclusion criteria: patients above 18 years of age scheduled for cardiac surgery; excessive postoperative bleeding, more than 250 ml for one hour, or more than 150ml for two hours during the first four hours  Exclusion criteria: patients younger than 18 years of age; patients with a medical condition known to influence the hemostatic system; patients treated with clopidogrel or systemic steroids during the last week before surgery; patients with INR above 1.5; patients who are not able to give written informed consent; unstable patients who need other transfusion limits than in this study |
| Interventions | Intervention arm: DDAVP 0.3 microgram/kg  Comparator arm: placebo (NaCl 0.9%) |
| Outcomes | Primary outcome: Transfusion of blood components [ Time Frame: During postoperative stay]  Secondary outcomes:  - Postoperative hemorrhage [ Time Frame: First 16 hours postoperatively]  - Platelet activation [ Time Frame: 20 hours postoperatively]  - Activation of coagulation [ Time Frame: 20 hours postoperatively |
| Notes | This trial was terminated (difficulties in recruitment)  Study Completion Date: February 2012.  No response from authors after 3 attempts. |

| Trial name or title | Study of DDAVP Combined with TXA on the Blood loss and Transfusion Need During and After Scoliosis Correction Surgery  Trial registry: NCT02084342 (https://clinicaltrials.gov/ct2/show/NCT02084342) |
| --- | --- |
| Methods | Type of study: single-centre, parallel-group, 2-arm RCT  Country where study is being performed: China |
| Participants | Inclusion criteria: idiopathic scoliosis patients undergoing posterior scoliosis correction surgery; American Society of Anesthesiologists (ASA) classification: I-II; patients who agreed to participate in this study and has signed the informed consent  Exclusion criteria: blood disease, such as anaemia, idiopathic thrombocytopenic purpura (ITP); history of bleeding or ecchymosis; disorders of laboratory examination on platelets (PLT), prothrombin time (PT), activated partial thromboplastin Time (aPTT), Fibrinogen-dimers; hypertension; cardiac disease, such as unstable angina, myocardial infarction in recent six months, cardiac disfunction, congenital heart disease, pulmonary heart disease; cerebral ischemia; administering with anticoagulants or nonsteroidal anti-inflammatory drug(NSAID); hepatic or renal disease or dysfunction; blood transfusion in recent one month |
| Interventions | Intervention arm: tranexamic acid and sodium chloride injection at 10mg/kg, IV (in the vein) for 30min, before incision, then at 1mg/kg/h, IV pump, until the surgery is over; desmopressin acetate injection at 0.3μg/kg dissolved in 100ml NS, IV for 20min, before incision  Comparator arm: tranexamic acid and sodium chloride injection at 10mg/kg, IV (in the vein) for 30min, before incision, then at 1mg/kg/h, IV pump, until the surgery is over; normal saline (NS) 100ml IV for 20min, before incision. |
| Outcomes | Primary outcome: blood loss [ Time Frame: during and 3 days after the surgery]  Secondary outcomes:  - blood transfusion [ Time Frame: during and 3 days after the surgery], the blood transfusion includes all the product needed during and in 3 days after the surgery.  - postoperative complications [ Time Frame: up to 24 weeks after the surgery] |
| Notes | Recruitment status: Unknown.  Expected completion date: June 2014.  No response from authors after 3 attempts. |

| Trial name or title | Safety Outcomes Post Kidney Biopsy – Randomized Clinical Evaluation of Efficacy of Desmopressin (STOP-BLEED)  Trial registry: NCT05467033 (<https://www.clinicaltrials.gov/ct2/show/NCT05467033>) |
| --- | --- |
| Methods | Type of Study: single-centre, parallel-group, 2-arm RCT  Country where study is being performed: Poland |
| Participants | Inclusion criteria: ≥ 18 years old; ability to provide informed consent; qualification by nephrologist to kidney biopsy in accordance with current standards; initial haemoglobin concentration > 8g/dl and PLT count >100 x103/μL; normal range of APTT and INR; blood pressure control defined as SBP<160mmHg; permitted anti-platelet/antithrombotic drugs: acetylsalicylic acid and heparin; no inflammation at the point of biopsy needle insertion  Exclusion criteria: Initial sodium concentration <130mmol/l;  pregnancy and breastfeeding; anaphylactic shock after  desmopressin administration (medical history); necessity of  administration other anti-platelet / anti thrombotic drugs other  than acetylsalicylic acid, non-fractionated heparin or low  molecular weight heparin; decompensated heart failure; von  Willebrand disease (VWD) type II B; as per investigator opinion a  medical situation which may lead to increased intracranial  pressure (ICP); hydronephrosis of the biopsied kidney; usage of  any prohibited drug before screening: ASA in dosage > 75mg per  day; Vitamin K antagonist (VKA); direct oral anticoagulants  (DOAC); low-molecular-weight heparin (LMWH); unfractionated  heparin (UFH); except situation when dosage of listed above drugs  will be adjusted in accordance to protocol |
| Interventions | Intervention arm: desmopressin 0.3ug/kg in 100ml 0,9% NaCl  managed as intravenous infusion  Comparator arm: placebo managed as intravenous infusion (0,9%  NaCl) |
| Outcomes | Primary outcome: Bleeding events in 24 hours after the procedure  [ Time Frame: 24 hours after kidney biopsy]; minor bleeding  events: macroscopic hematuria; clinically silent hematoma in  ultrasound performed 24h after biopsy, decrease in  hemoglobin concentration >20% of baseline; major bleeding  events: erythrocyte transfusion, embolization, nephrectomy,  death related directly or indirectly to bleeding  Secondary outcomes: Bleeding events in 48 hours after the procedure  [ Time Frame: 48 hours after kidney biopsy]; minor bleeding  events: macroscopic hematuria; clinically silent hematoma in  ultrasound performed 24h after biopsy, decrease in  hemoglobin concentration >20% of baseline; major bleeding  events: erythrocyte transfusion, embolization, nephrectomy,  death related directly or indirectly to bleeding |
| Notes | Expected completion date: August 31, 2026  Received response from Dr. Rydzewska-Rosolowska: study has completed 10% recruitment, no data available to share. |

| Trial name or title | Comparison of the effect of tranexamic acid and intravenous  desmopressin on reducing bleeding during septorhinoplasty  Trial registry: IRCT20200124046241N1 (<https://en.irct.ir>) |
| --- | --- |
| Methods | Type of study: single-centre, parallel-group, 3-arm RCT  Country where study is being performed: Iran |
| Participants | Inclusion criteria: Perform septorhinoplasty, no coagulation  disorders, 18 years until 50 years, ASA-1  Exclusion criteria: contraindications for the administration of  tranexamic acid and desmopressin, dissatisfaction with research,  drug addiction, being a smoker |
| Interventions | Intervention arm I: tranexamic acid injected half an hour before  surgery at a dose of 5 mg/kg  Intervention arm II: DDAVP injected at 4 μg / ml half an hour  before surgery  Comparator arm: same amount of placebo given with similar  characteristics |
| Outcomes | Primary outcome: Bleeding during surgery, determined by the volume of the suction and the number of blood gases  Secondary outcomes:  - Blood pressure during surgery, determined with barometer  - Heart rate during surgery determined with pulse oximeter |
| Notes | Recruitment status: Completed: August 21, 2019  No response from authors after 3 attempts. |

| Trial name or title | The effect of intravenous desmopressin in coagulation profile of  patients undergoing valve surgery: a randomized clinical study  Authors: Santana, G; Hajjar, L; Camara, L; Piccioni, M; Galas, L; Galas, F.  Year: 2018. |
| --- | --- |
| Methods | Type of study: single-centre, parallel-group, 2-arm RCT  Country where study is being performed: Brazil |
| Participants | Inclusion criteria: not reported  Exclusion criteria: not reported |
| Interventions | Intervention arm: DDAVP (0.3 μg/kg)  Comparator arm: not reported |
| Outcomes | Primary outcome:  - Post-operative bleeding  - Number of transfused units |
| Notes | No response from authors after 3 attempts. |

| Trial name or title | The Effect Of Desmopressin Intraoperatively On Hemorrhage During The Rhinoplasty  Trial registry: IRCT20170120032069N7 (<https://trialsearch-who-int.proxy1.lib.uwo.ca/Trial2.aspx?TrialID=IRCT20170120032069N7>) |
| --- | --- |
| Methods | Type of study: Single-center, parallel-group, 2-arm RCT  Country where study is being performed: Iran |
| Participants | Inclusion criteria: Volunteers with no age limit who undergo the Rhinoplasty  Exclusion criteria: Cardiovascular disease; Bleeding disorders; High blood pressure; Allergy to Desmopressin; History of cerebrovascular attack; Seizure |
| Interventions | Intervention arm: Thirty minutes before the surgery, 500 ml of normal saline containing 0.1µgr / kg of Desmopressin is injected (N=35)  Comparator arm: Thirty minutes before the surgery, 500 ml of normal saline is injected (N=35) |
| Outcomes | Primary outcome(s):  - The time of surgery. Timepoint: Measuring The time of surgery at the end of surgery. Method of measurement: Duration of surgery from the beginning to the end of the surgery.  - Bleeding during rhinoplasty surgery. Timepoint: Measuring the amount of bleeding at the end of surgery. Method of measurement: The sucker bottle which is calibrated measures bleeding loss.  - View of surgeon on operating field. Timepoint: Measuring surgeon satisfaction at the end of surgery. Method of measurement: based on the FROMME-BOEZAART Scale.  Secondary outcome(s): Bleeding will be measured by the surgeon based on the FROMME-BOEZAART Scale. Timepoint: During the surgery. Method of measurement: Based on this scale, there are five grades in which the surgeon during the surgery scores the bleeding. Grade 0: No bleeding (cadaveric conditions); grade 1: Slight bleeding, no suctioning required; grade 2: Slight bleeding, occasional suctioning required; grade 3: Slight bleeding, frequent suctioning required; bleeding threatens surgical field a few seconds after suction is removed; grade 4:Moderate bleeding, frequent suctioning required, and bleeding threatens surgical field directly after suction is removed; grade 5:Severe bleeding, constant suctioning required; bleeding appears faster than can be removed by suction; surgical field severely threatened and surgery usually not possible. |
| Notes | Expected completion date: NA  No response from authors after 3 attempts. |

| Trial name or title | The effect of desmopressin on reducing blood loss in children with congenital cyanotic heart disease undergoing cardiopulmonary bypass  Trial registry: PACTR202004798675261 (<https://trialsearch-who-int.proxy1.lib.uwo.ca/Trial2.aspx?TrialID=PACTR202004798675261>) |
| --- | --- |
| Methods | Type of study: Single-center, parallel-group, 2-arm RCT  Country where study is being performed: Egypt |
| Participants | Inclusion criteria: Patients with CCHD undergoing CPB of either sex with their age ranging from 3 to 72 month  Exclusion criteria: Patients will be excluded from the current study in case of refusal of their guardians; redo cardiac surgery; pre existing coagulopathy; hepatic or renal dysfunction and history of allergy to desmopressin |
| Interventions | Intervention arm: DDAVP 0.3 ug /kg in 20 mL of normal saline over 30minutes after induction of anesthesia through a central venous catheter. This dose will be repeated every 6 hours for 24 hours. (N=26)  Comparator arm: Patients will receive a 20mL placebo over 30 minutes after induction of anesthesia through a central venous catheter. This dose will be repeated every 6 hours for 24 hours. (N=26) |
| Outcomes | Primary outcome(s): The total amount of blood loss drained through mediastinal and pleural drainage tubes during the first 24 postoperative hours  Secondary outcome(s):  - Rate RBCS transfusion  - Hours of mechanical ventilation  - Duration of intensive care unit, and hospital stay |
| Notes | Expected completion date: NA  No response from authors after 3 attempts. |

| Trial name or title | DDAVP for bleeding prophylaxis in patients undergoing kidney biopsy procedures: A randomized controlled trial  Trial registry: TCTR20220321004 (<https://trialsearch-who-int.proxy1.lib.uwo.ca/Trial2.aspx?TrialID=TCTR20220321004>) |
| --- | --- |
| Methods | Type of study: Single-center, 1-arm, open label study  Country where study is being performed: Thailand |
| Participants | Inclusion criteria: Native kidney biopsy, GFR> 30 ml/min/1.73m2 (Thai CKD-EPI)  Exclusion criteria: Kidney Transplant; Single kidney; hydronephrosis; small kidney < 8 cm; multiple cystic kidney; Bleeding tendency (plt<120,000/u, INR > 1.5, patients who do not withdraw antiplatelet or anticoagulant); Hyponatremia; BMI > 35 kg/ m2; Pregnancy; Patient who did not agree |
| Interventions | Intervention arm: DDAVP 0.3 mcg/kg/dose intravenous prior to kidney biopsy add on standard bleeding prevention (N=160)  Comparator arm: NA |
| Outcomes | Primary outcome(s):  - Bleeding composite outcome 1 day after kidney biopsy Hb drop more than 1 g/dl  - Hematoma size more than 2 cm  - Persistent gross hematuria or urinary tract obstruction  - Major bleeding outcome  Secondary outcome(s):  - 1 day size of perinephric hematoma  - AKI  - Hospital stay  - Adverse outcome of DDAVP  - Hyponatremia  - Thrombotic event  - Composite bleeding outcome in GFR more than 60 ml/min/1.73m2 |
| Notes | Expected completion date: March 30, 2023  No response from authors after 3 attempts. |

| Trial name or title | The efficacy of Desmopressin Acetate in reduction of post kidney biopsy bleeding among renal insufficiency patients: A randomized controlled trial.  Trial registry: TCTR20150203001 (<https://trialsearch-who-int.proxy1.lib.uwo.ca/Trial2.aspx?TrialID=TCTR20150203001>) |
| --- | --- |
| Methods | Type of study: Parallel-group, 2-arm RCT  Country where study is being performed: Thailand |
| Participants | Inclusion criteria: Age >/= 18 years old; Native kidney; Creatinine >/= 1.5 to < 4 mg/dL; BP < 160/80 mmHg before kidney biopsy; Normal platelet count and coagulogram  Exclusion criteria: Single kidney; Kidney cancer; Hydronephrosis or pyelonephritis; BMI > 35 kg/m2; Dialysis free time < 2 weeks in case indicated dialysis; Different kidney size more than 2 cm; The patient who takes antiplatelets or anticoagulants; Allergy to DDAVP |
| Interventions | Intervention arm: DDAVP 0.3µg/kg in NSS 50 ml iv. 30 minutes before kidney biopsy (N not available)  Comparator arm: NSS 50 ml iv. 30 minutes before kidney biopsy (N not available) |
| Outcomes | Primary outcome(s):  - Minor bleeding complication: bleeding without blood transfusion nor intervention 6 hours, 24 hours after intervention hemoglobin, hematoma size by ultrasound  - Major bleeding complication: bleeding with requirement of blood transfusion or intervention 6 hours, 24 hours after intervention hemoglobin, hematoma size by ultrasound, blood transfusion, angiogram with embolization  Secondary outcome(s):  - Hematoma size between 2 groups 6 hours ultrasound  - The different vWF Ag, RCO, CBA levels between 2 groups 1 hour after DDAVP injection measurement of vWF Ag, RCO, CBA level  - The difference between platelet aggregation test between 2 groups 1 hour after DDAVP injection platelet aggregation test  - Side effect of DDAVP 6 hours after DDAVP injection plasma sodium |
| Notes | Expected completion date: September 30, 2015  No response from authors after 3 attempts. |

| Trial name or title | Desmopressin, as a hemostatic agent, should always be given with tranexamic acid? A pilot study  Authors: Spyridakis, E; Fotopoulou, G; Pappa, E; Kalakonas, S  Year: 2018 |
| --- | --- |
| Methods | Type of study: Single-center, parallel-group, 2-arm RCT  Country where study is being performed: Greece |
| Participants | Inclusion criteria: Patients undergoing primary and isolated on-pump CABG surgery admitted to the cardiovascular intensive care unit between December 2017 and March 2018  Exclusion criteria: NA |
| Interventions | Intervention arm: DDAVP 0,6 γ/kg, divided in two doses, at the beginning and at the end of surgery (N=27)  Comparator arm: TXA 20 mg/kg at induction of anesthesia,10 mg/kg during CPB and 30 mg/kg with protamine injection, as well as DDAVP 0,6 γ/kg, divided in two doses, at the beginning and at the end of surgery (N=28) |
| Outcomes | Primary outcome(s):   - Pre and post-operative levels of D-dimers as well as visible blood loss defined as intra- and postoperative bleeding during the first 24 hours   Secondary outcome(s):   - NA |
| Notes | Expected completion date: NA  No response from authors after 3 attempts. |

| Trial name or title | Effects of Prophylactic Desmopressin on Blood Coagulation Parameters in Heart Valve Surgery  Trial registry: NCT03343418 (https://clinicaltrials.gov/ct2/show/study/NCT03343418) |
| --- | --- |
| Methods | Type of study: Parallel-group, 2-arm RCT  Country where study is being performed: Brazil |
| Participants | Inclusion criteria: Valve cardiac surgery with cardiopulmonary bypass; Written informed consent; age 18 to 80 years  Exclusion criteria: Reoperative valve surgery; Hematocrit < 35%; Ventricular dysfunction (EF < 40%); Infection; Body mass index > 35; Renal impairment (Creatinin > 2mg/dL); Antiplatelet administration within 10 days preceding study surgery; Participation in another interventional clinical study within 30 days; Known or suspected hypersensitivity to the desmopressin; Coagulopathy (INR > 1.5) |
| Interventions | Intervention arm: Desmopressin is to be administered as an intravenous infusion after discontinuation of cardiopulmonary bypass and administration of protamine. Subjects are to be given desmopressin 0,3 microgram.kg-1. After desmopressin infusion, bleeding treatment will follow the predefined standardized treatment regimen (N not available)  Comparator arm: 0.9% saline is to be administered as an intravenous infusion after discontinuation of cardiopulmonary bypass and administration of protamine. Patients randomized to the control group will receive the infusion of 100 milliliters (mL) 0.9% saline (SF0,9%). After 0.9% saline infusion, bleeding treatment will follow the predefined standardized treatment regimen (N not available) |
| Outcomes | Primary outcome(s):  - Change in coagulation parameters [ Time Frame: Coagulation parameters will be performed: T0: preoperative period; T1 - 2 hours after desmopressin or placebo administration; T2 - 24 hours after desmopressin or placebo administration ] Laboratory variables: Activated partial thromboplastin time [(aPTT) seconds], prothrombin time [(PT) seconds], haematocrit (%), haemoglobin(g/dl), fibrinogen(mg/dL), D dimer (ng/L), factor VIII(%), Von Willebrand factor(%), ROTEM [(INTEM, EXTEM, FIBTEM) Clotting Time (CT) and Clot Formation Time (CFT) seconds, alpha angle (α º), Maximum Clot Firmness (MCF) mm ] and platelets aggregation (%)  - Postoperative blood loss [ Time Frame: 48 hours ] Total chest tube drainage (mL) starting immediately after closure of the chest in the operating theater until 48 hours  Secondary outcome(s):  - Blood transfusion [ Time Frame: 30 days ] Number of units of red blood cells, fresh frozen plasma, platelets, cryoprecipitate in the operating theater and ICU  - Reoperation for bleeding [ Time Frame: 30 days ]  - Duration of Mechanical ventilation [ Time Frame: 30 days ]  - Length of vasoactive drugs [ Time Frame: 30 days ]  - Length of ICU stay [ Time Frame: 30 days ]  - Clinical complications - renal failure, infection, myocardial ischemia, stroke [ Time Frame: 30 days ]  - Length of hospital stay [ Time Frame: 30 days ]  - Mortality [ Time Frame: 30 days ] |
| Notes | Expected completion date: November 1, 2017  No response from authors after 3 attempts. |

| Trial name or title | Intervention pilot with fibrinogen concentrate and the combination of fibrinogen concentrate with FFP in patients with massive post/peri-operative bleeding problems  Trial registry: NTR1218 (<https://trialsearch-who-int.proxy1.lib.uwo.ca/Trial2.aspx?TrialID=NTR1218>) |
| --- | --- |
| Methods | Type of study: Single-center, parallel-group RCT  Country where study is being performed: Netherlands |
| Participants | Inclusion criteria: General surgery patients with massive peri-operative blood loss, with an indication of receiving 4 units of FFP according to current guidelines; Cardiothoracic surgery patients after heart valve replacement who met the criteria of increased blood loss and indication for DDAVP  Exclusion criteria: No other therapeutical anti-thrombotics are given, other than platelet-aggregation inhibitors; Patients with active HIV-infection; Patients who received FFP or fibrinogen just before the surgery; Patients who are hypothermia (< 30C) or with a circulatory arrest; Patients with a fibrinogen level > 2 g/L |
| Interventions | Comparing FFP transfusion in massive bleeding patients with the combination of fibrinogen and FFP/DDAVP |
| Outcomes | Primary outcome(s):  - Stop of bleeding after the therapy. The clinical situtation after the treatment will be compared with conventional coagulation tests (aPTT, PT, fibrinogen level)  Secondary outcome(s):  - Thrombin generation and thromboelastography measurements, measured with the CAT en TEG method |
| Notes | Expected completion date: NA  No response from authors after 3 attempts. |

| Trial name or title | The Effect of Desmopressin on The Post Operative Bleeding in Patients Undergoing Open Heart Surgery  Trial registry: IRCT20200424047185N1 (http:en.irct.ir) |
| --- | --- |
| Methods | Type of study: Single-center, parallel-group, 2-arm RCT  Country where study is being performed: Iran |
| Participants | Inclusion criteria: All patients are candidates for open heart surgery subject to informed consent Conscious dissatisfaction  Exclusion criteria: Blood coagulation disease |
| Interventions | Intervention arm: The intervention group will receive 0.3 micrograms per kilogram of desmopressin half an hour before the start of surgery (N not available)  Comparator arm: The control group will receive 0.3 micrograms per kilogram of 0.9 sodium chloride half an hour before the start of surgery (N not available) |
| Outcomes | Primary outcome(s):  - Postoperative bleeding, Timepoint: Measure the patient's bleeding rate two, six, eight, twelve, and twenty-four hours after surgery, Method of measurement: The amount of blood in the Chest Bottle is in milliliters  Secondary outcome(s): NA |
| Notes | Expected completion date: NA  No response from authors after 3 attempts. |
